# Osteopontin depletion in macrophages perturbs proteostasis *via* regulating UCHL1-UPS axis and mitochondria-mediated apoptosis

**DOI:** 10.3389/fimmu.2023.1155935

**Published:** 2023-05-30

**Authors:** Altan Rentsendorj, Koen Raedschelders, Dieu-Trang Fuchs, Julia Sheyn, Vineet Vaibhav, Rebecca A. Porritt, Haoshen Shi, Jargalsaikhan Dagvadorj, Juliana de Freitas Germano, Yosef Koronyo, Moshe Arditi, Keith L. Black, Bhakta Prasad Gaire, Jennifer E. Van Eyk, Maya Koronyo-Hamaoui

**Affiliations:** ^1^ Department of Neurosurgery, Maxine Dunitz Neurosurgical Research Institute, Cedars-Sinai Medical Center, Los Angeles, CA, United States; ^2^ Smidt Heart Institute, Cedars-Sinai Medical Center, Los Angeles, CA, United States; ^3^ Department of Biomedical Sciences, Cedars-Sinai Medical Center, Los Angeles, CA, United States; ^4^ Department of Pediatrics, Cedars-Sinai Medical Center, Los Angeles, CA, United States; ^5^ Department of Medicine, Cedars-Sinai Medical Center, Los Angeles, CA, United States; ^6^ Department of Neurology, Cedars-Sinai Medical Center, Los Angeles, CA, United States

**Keywords:** secreted phosphoprotein 1, early T-lymphocyte activation (ETA-1), bone/sialoprotein I (BSP-1 or BNSP), monocytes, innate immunity, mitochondrial dysfunction, reactive oxygen species

## Abstract

**Introduction:**

Osteopontin (OPN; also known as SPP1), an immunomodulatory cytokine highly expressed in bone marrow-derived macrophages (BMMΦ), is known to regulate diverse cellular and molecular immune responses. We previously revealed that glatiramer acetate (GA) stimulation of BMMΦ upregulates OPN expression, promoting an anti-inflammatory, pro-healing phenotype, whereas OPN inhibition triggers a pro-inflammatory phenotype. However, the precise role of OPN in macrophage activation state is unknown.

**Methods:**

Here, we applied global proteome profiling via mass spectrometry (MS) analysis to gain a mechanistic understanding of OPN suppression versus induction in primary macrophage cultures. We analyzed protein networks and immune-related functional pathways in BMMΦ either with OPN knockout (OPN*^KO^*) or GA-mediated OPN induction compared with wild type (WT) macrophages. The most significant differentially expressed proteins (DEPs) were validated using immunocytochemistry, western blot, and immunoprecipitation assays.

**Results and discussion:**

We identified 631 DEPs in OPN*^KO^* or GA-stimulated macrophages as compared to WT macrophages. The two topmost downregulated DEPs in OPN*^KO^* macrophages were ubiquitin C-terminal hydrolase L1 (UCHL1), a crucial component of the ubiquitin-proteasome system (UPS), and the anti-inflammatory Heme oxygenase 1 (HMOX-1), whereas GA stimulation upregulated their expression. We found that UCHL1, previously described as a neuron-specific protein, is expressed by BMMΦ and its regulation in macrophages was OPN-dependent. Moreover, UCHL1 interacted with OPN in a protein complex. The effects of GA activation on inducing UCHL1 and anti-inflammatory macrophage profiles were mediated by OPN. Functional pathway analyses revealed two inversely regulated pathways in OPN-deficient macrophages: activated oxidative stress and lysosome-mitochondria-mediated apoptosis (*e.g*., ROS, Lamp1-2, ATP-synthase subunits, cathepsins, and cytochrome C and B subunits) and inhibited translation and proteolytic pathways (*e.g*., 60S and 40S ribosomal subunits and UPS proteins). In agreement with the proteome-bioinformatics data, western blot and immunocytochemical analyses revealed that OPN deficiency perturbs protein homeostasis in macrophages—inhibiting translation and protein turnover and inducing apoptosis—whereas OPN induction by GA restores cellular proteostasis. Taken together, OPN is essential for macrophage homeostatic balance via the regulation of protein synthesis, UCHL1-UPS axis, and mitochondria-mediated apoptotic processes, indicating its potential application in immune-based therapies.

## 1 Introduction

Osteopontin (OPN), encoded by the *secreted phosphorylated protein 1* (*Spp1*) gene, is a multifaceted matricellular glycoprotein secreted by various immune cells, such as macrophages and T cells (1–3). OPN is an immunomodulatory cytokine that is highly expressed by bone marrow-derived macrophages (BMMΦ) and regulates diverse cellular immune responses, including migration, communication, and immunological responses (4–6). Notably, OPN has dual roles in peripheral immune cells, influencing both inflammatory and anti-inflammatory responses depending on the acute or chronic inflammatory microenvironment. Although previous studies have reported that OPN is associated with type 1 pro-inflammatory macrophage and T cell polarization (4, 7), other reports demonstrate that OPN is associated with macrophage and T cell polarization towards anti-inflammatory phenotypes (6, 8–10). In addition, macrophage-derived OPN induces an anti-inflammatory immune response during *Cryptococcus neoformans* infection (11). Furthermore, a recent report revealed that OPN can reduce inflammation, tissue injury, and bacterial loads during concurrent pneumococcal infection in a murine model (10). These studies suggest dual roles for OPN in regulating peripheral immune functions, and strict modulation of OPN function is crucial to accomplishing optimal immune responses.

In the context of the central nervous system (CNS), OPN has been shown to be a critical regulator of neuroprotection in several neurological disorders, such as brain ischemia, stroke, and traumatic brain injury (12–14). Notably, macrophages are the key regulators of immune responses due to their rapid and diverse activities. Macrophages exhibit high plasticity and adaptability to various adverse conditions, which promote repair and restore tissue homeostasis (15–18). Hence, regulating macrophage OPN could have a significant impact on various peripheral and CNS inflammatory conditions.

We recently reported that stimulating BMMΦ with glatiramer acetate (GA; generic name Copaxone^®^), an FDA-approved drug for the treatment of the neurological autoimmune disease, relapsing-remitting multiple sclerosis, upregulates OPN expression, promoting an anti-inflammatory response (6). Our *in vivo* and *in vitro* studies indicated that GA-stimulation of macrophages substantially induces their ability to clear pathogenic forms of amyloid β-protein (Aβ), including Aβ_40_ and Aβ_42_ fibrils. Moreover, GA treatment protected both synapses and cognitive function in transgenic murine models of Alzheimer’s disease (AD) (19, 20). We found that GA immunomodulation can lead to increased cerebral infiltration of monocytes, which are directly involved in Aβ plaque clearance (6, 19–22). Furthermore, in BMMΦ cultures, GA exposure improved phagocytosis of fibrillar Aβ *via* increased expression of surface scavenger receptors (i.e., CD36, Scara-1). These receptors recognize and bind Aβ fibrils to facilitate their uptake (19, 20). Importantly, GA promoted a shift into anti-inflammatory microglia and monocyte/macrophage phenotypes, as evidenced by marked increases in anti-inflammatory interleukin (IL)-10, insulin-like growth factor-1 (IGF-1), matrix metalloproteinase-9 (MMP-9), and OPN production, as well as decreases in pro-inflammatory cytokines such as tumor necrosis factor-alpha (TNF-α) and IL-12 release (6, 19, 23, 24).

Recent proteomic studies on microglia and macrophages have identified distinct protein expression profiles of pro-inflammatory versus anti-inflammatory-type macrophages related to cell metabolism and signaling (25–29). Additionally, a few whole proteome analyses have been conducted to decipher GA’s effects and mechanisms of action (30, 31). In the current study, we applied quantitative mass spectrometry (MS)-based proteomics profiling to analyze the effects of OPN deficiency vs. GA immune modulation on macrophage protein networks and functional pathways. Top up and down-regulated proteins were defined as differentially expressed proteins (DEPs), whereas protein expression networks were examined using functional clustering analysis. Then, proteins were enriched for canonical pathways to identify specific signaling pathways. Upstream regulator analysis was performed to identify molecules that can influence the changes in downstream signaling. We comprehensively identified and quantified proteins from OPN-deficient and GA-stimulated BMMΦ relative to untreated macrophages through proteomics. In addition, key identified DEPs were validated *in vitro* by employing immunocytochemistry, immunoprecipitation, Western blot, and mitochondrial tracing analyses. Findings from this study provide new insights into the immune mechanisms of OPN deficiency in macrophages, affecting cellular homeostasis and survival *via* reduced ubiquitin-proteosome system (UPS) functions.

## 2 Materials and methods

### 2.1 Mice

OPN knock-out (OPN*^KO^*) mice from the B6.129S6 (Cg)-*Spp1^tm1Blh^*/J strain (Jackson laboratories stock #004936|OPN*^KO^*) and aged-matched wild type (WT) controls (Jackson laboratories) were used for *in vitro* experiments. Animal experiments were performed in accordance with Cedars-Sinai Medical Center Institutional Animal Care and Use Committee (IACUC) guidelines under an approved protocol.

### 2.2 Primary cultures of bone marrow-derived macrophages

Bone marrow-derived macrophages (BMMФ) were collected from 8- to 16-week-old WT or OPN*^KO^* mice and cultured as previously described (6). On the sixth day of culture, cells were either plated in a 24-well plate on glass coverslips for immunocytochemistry (1x10^5^ cells per well; 3-6 wells per condition) or a 6-well plate for protein assays (3x10^5^ cells per well; 3 wells per condition). Next, the wells were treated with either 30 µg/mL GA (TEVA; Glatopa), 50 ng/mL recombinant OPN (Peprotech #120-35), 10 µM minocycline (Millipore sigma #M9511), or stealth OPN siRNA (Invitrogen) for 24 hours. The control groups were the untreated WT and the GA-treated OPN*^KO^* cells. Each experiment was repeated 2-4 times. Cells were washed three times with 1X PBS before fixation with 4% paraformaldehyde (15 min at room temperature) or ice-cold methanol at -20°C. Then, the cells were rinsed 3 times with 1X PBS and stored at 4°C until further analysis.

### 2.3 Immunocytochemistry

Cells plated on glass coverslips were blocked with serum-free protein blocking (Dako Cytomation) for 30 min at room temperature, then hybridized with primary antibodies overnight at 4°C (see **Table S1**). On the following day, cells were incubated with appropriate secondary polyclonal antibodies for 1 hour at room temperature. Coverslips were then mounted using ProLong Gold with DAPI (Molecular Probes, Life Technologies). Negative controls were processed using the same protocol with the omission of the primary antibody to assess non-specific labeling (**Figure S4A**).

In cases where cells were stained in parallel with Dihydroethidium (DHE; Sigma-Aldrich #D7008), fluorescent dye was added after the secondary antibody for 30 min at 37°C (10 μM final concentration in PBS) before mounting.

### 2.4 Mitotracker

Cells treated with Mitotracker™ (Thermofisher #M7512) were incubated for 15-30 min with 250 μl of the fluorescent dye at 37°C, then washed three times with 1X PBS before fixation with 4% PFA (15 min at room temperature). Cells were rinsed three times with 1X PBS and mounted for microscopy analysis.

### 2.5 Microscopy and quantification

Representative images were captured with a Carl Zeiss Axio Imager Z1 fluorescence microscope equipped with ApoTome, AxioCam MRm, and AxioCam HRc cameras (Carl Zeiss MicroImaging, Inc.) using the same exposure time for each channel across all images. Images were analyzed using ImageJ software (NIH), converted to grayscale, and processed with the same post-acquisition threshold for analysis. Five to ten images from each well (n=3-5 wells), covering an average of 100 cells per image, were analyzed.

### 2.6 Western blot

Cells plated on 6-well plates were lifted with 2 mM EDTA-PBS, collected in tubes, and pelleted (1000 rpm for 5 min). Cell pellets were lysed in RIPA lysis buffer (Thermofisher Scientific, #89900) supplemented with 1% protease inhibitor (Calbiochem #539131) and stored at -80°C until further analysis. Protein concentration was determined using a BCA Protein Assay Kit (Thermofisher Scientific, #23225). Equal amounts of total protein samples were electrophoretically separated onto 4% to 20% Tris-glycine gels (Invitrogen, #XP04205BOX), then transferred to nitrocellulose membranes, blocked for 1 hour at room temperature in Tris-buffered saline with Tween 20 (TBST; 10 mmol/L Tris-HCl buffer, pH 8.0, 150 mmol/L NaCl, and 0.1% Tween 20) containing 5% BSA, and hybridized with appropriate primary antibodies overnight at 4°C. After four washes in TBST, membranes were incubated with HRP-conjugated secondary antibody for 1 hour at room temperature, then washed again four times in TBST prior to development with a chemiluminescence substrate kit (Thermofisher Scientific, #34580). Images were taken on an iBright imaging system (iBright imaging system; Thermofisher Scientific). Protein expressions were analyzed using ImageJ software and normalized to β-Actin or GAPDH. Two to eight experimental repeats were performed for each experiment. Representative blots are shown for each protein.

### 2.7 Immunoprecipitation

Cells were lysed in lysis buffer (Pierce™ IP Lysis Buffer, Cat. 87787) on ice for 20 min, and lysates were centrifuged at 8000 rpm for 10 min. The supernatant was collected and incubated with 50 ml of IP beads (TrueBlot Anti-Rabbit) for 30 min on a rocking platform at 4°C. After incubation, the supernatant was collected by centrifugation and added 5 mg of the primary antibody and incubated on a rocking platform overnight at 4°C. After overnight incubation, 50 ml of IP beads was added to the supernatant, and after 1 hour the beads were collected by centrifugation and washed with lysis buffer. Beads were then loaded with 2X loading buffer and run on sodium dodecyl-sulfate polyacrylamide gel electrophoresis.

### 2.8 LC-MS/MS analysis

#### 2.8.1 Sample preparation for mass spectrometry

Cell pellet processing for MS as outlined in Parker et al. (32). Briefly, proteins were reduced with 5 mm TCEP, alkylated with iodoacetamide, digested with sequencing grade modified trypsin (Promega, Madison, WI) at a 1:100 ratio of enzyme/substrate, and the digestion was stopped by the addition of formic acid. Tryptic peptides were desalted using reverse phase cartridges Sep-Pak C18 (Waters, Milford, MA) according to the following procedure. The tryptic peptides were dried using a vacuum centrifuge and then resolubilized in 0.1% formic acid with synthetic iRT calibration peptides (Biognosys, Schlieren, Switzerland) at a 1:20 v/v ratio.

#### 2.8.2 MS including assay library generation and individual sample analysis by data independent acquisition-MS

The tryptic peptides were separated using a ChromXP column ((3 um particle size, 150 um x 10 cm) Eksigent technologies) from 3–35% acetonitrile over 60 min gradient using an Eksigent NanoLC Ultra 2D Plus HPLC system coupled to a 5600 TripleTOF mass spectrometer (AB Sciex, Framingham, MA). To build the sample-specific peptide library, the tryptic peptides data was generated using data-dependent acquisition as outlined in Parker et al. (32). Briefly, the top 20 most intense MS1 precursors (collected between 360 and 1460 *m*/*z* for 250 msec) with charge states between 2 and 5 were selected for MS2 fragmentation, with a 15 sec exclusion window. Fragment MS2 ions were collected for 100 ms across a 50–2000 *m*/*z* range. The raw MS spectra were converted to mzML using AB Sciex converter (v1.3) and subsequently converted to mzXML using msconvert (ProteoWizard, v3.04.238). They were then searched parallelly using OMSSA and X!Tandem algorithms Swiss-Prot–reviewed canonical mouse FASTA database (mouse database, 2013) appended with Biognosys iRT peptides for retention time alignment (Biognosys, Schlieren, Switzerland) and randomized decoy sequences. Search engine results were then converted to pepXML format using omssa2pepXML (v2.1.9) and Tandem2XML (v4.6.0). Peptide spectral match probability scoring was modeled in PeptideProphet (v4.6.0), and the resulting interact.pepXML files of the two search engines were combined in iProphet (v4.6.0). A Peptide assay library was generated with SpectraST (v4.0) from the identified peptides with a Peptide Prophet probability > 0.95. The resulting spectrast.splib file was submitted as input to the custom *spectrast2spectrast_irt.py* converter script that was used to align RT using iRT peptides. The spectral libraries were then formatted for OpenSWATH first using the custom script *spectrast2tsv.py*, followed by the OpenSWATH tool *ConvertTSVtoTraML*. Finally, *OpenSwathDecoyGenerator* was used to append shuffled decoys to the full assay library.

Each individual sample was analyzed by DIA-MS on the tripleTOF 5600 mass spectrometer in data-independent mode as outlined in Parker et al. (32), and Howlenski et al. (33). In DIA-MS, MS1 was collected with 100 *m*/*z* windows across a 400–1200 *m*/*z* range, with 0.5 *m*/*z* overlap at either end of a given window. Data were analyzed using OpenSWATH workflow (REF). Briefly, raw intensity data for peptide fragments were extracted from the DIA files using the open source openSWATH workflow against the custom-generated library. Target and decoy peptides were then extracted, scored, and analyzed using the mProphet algorithm to determine scoring cut-offs consistent with 1% FDR. The peak group extraction data from each DIA file was combined using the “featurealignment” script, which performed data alignment and modeling analysis across the experimental data set. The normalized transition-level data was then processed using the mapDIA software to perform pairwise comparisons between groups at the peptide level.

### 2.9 Functional network and computational analysis

Detectable protein hierarchies displayed as heatmaps and principal component analysis (PCA) were created by using ClustVis (https://biit.cs.ut.ee/clustvis/). Volcano plots were created using Prizm 9. Pie chart of Protein annotation through evolutionary relationship (PANTHER) protein classification analysis was created using http://pantherdb.org/geneListAnalysis.do. Data was analyzed using IPA (Ingenuity Pathway Analysis, Qiagen (https://digitalinsights.qiagen.com)). Differentially expressed genes (with corresponding fold-changes and *p* values) were incorporated in canonical pathways and upstream regulators analyses and were used to generate diagrams.

The 1.2-fold change difference and p ≤ 0.05 were selected as the cutoff since modest numbers of DEPs were observed between the different experimental groups.

Data have been deposited to the MassIVE repository under number MSV000091221 (https://urldefense.com/v3/:http://massive.ucsd.edu/ProteoSAFe/status.jsp?task=05165f2ddf9f4eecbf5465d94e0c3607:;!!KOmnBZxC8_2BBQ!xzewe2MHK3WeGKFzdYro-c2tOjsxpz26J1s-qZmQbIwJkyhY4DBmbuUHbC2NQ0bPQL3yuRBxBpYSM-lz_ervsQaZsDw$). Proteome Exchange ID: PXD039950.

### 2.10 Statistical analysis

Experimental data were analyzed using GraphPad Prism (GraphPad Software). One-way ANOVA with Tukey’s multiple comparison test was performed in case of three group comparisons. Two-tailed unpaired Student’s t-tests were used for two-group comparisons. The results are shown as means ± standard errors of the mean (SEM). Degree of significance between groups is represented as follows: *p<0.05, **p<0.01, ***p<0.001, and ****p<0.0001. A p-value lower than 0.05 was considered significant.

## 3 Results

### 3.1 Proteome signatures of OPN-deficient and GA-stimulated macrophages

**Figure 1:**
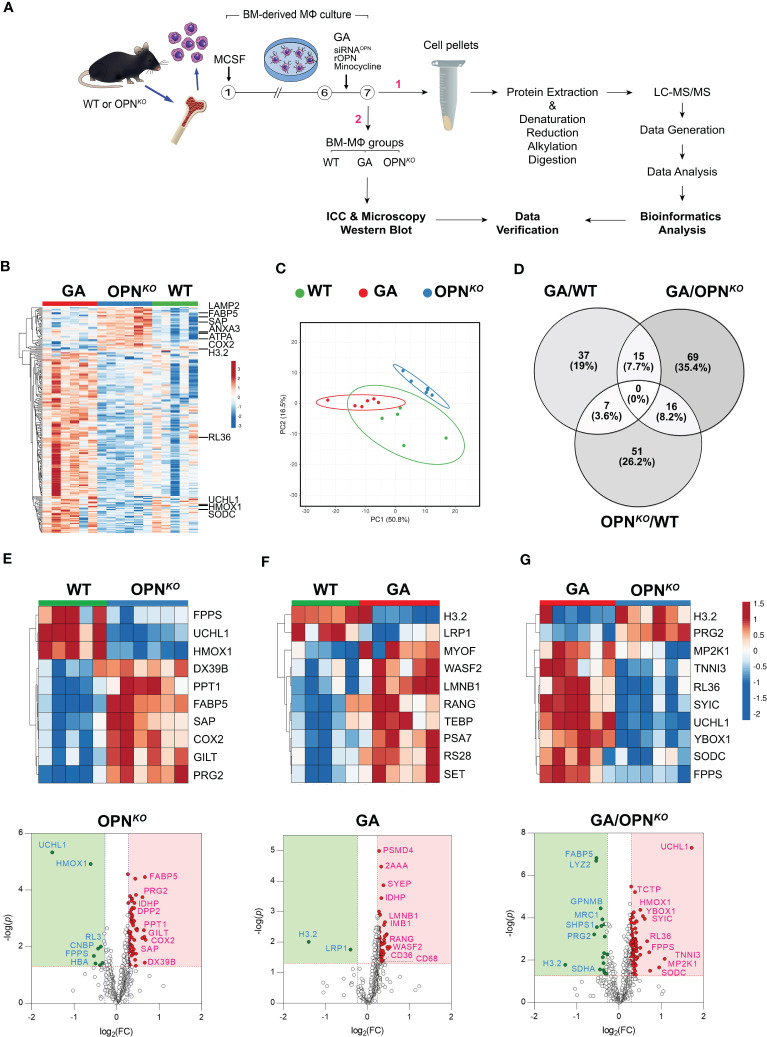
Proteome signatures of OPN-deficient and GA-stimulated macrophages. **(A)** Schematic illustration of experimental workflow for the proteomics profiling and validation studies. **(B)** Heatmap with hierarchical clustering analysis of the most abundant up (red)- and down (blue)-regulated DEPs across three experimental groups. **(C)** Hierarchical clustering of the top up and downregulated 225 DEPs by principal component analysis (PCA performed with ClustVis). Unit variance scaling is applied to rows; SVD with imputation is used to calculate principal components. Prediction ellipses are such that with probability of 0.95, a new observation from the same group will fall inside the ellipse. **(D)** Venn diagram showing the number and percentage (%) of overlapping DEPs according to statistical significance (*p*<0.05) and 1.2-fold change threshold criteria in the three analyzed groups. The number of common DEPs between pair groups is also shown. **(E–G)** Heatmaps and volcano plots of the top 10 most DEPs (FDR < 0.05, ranked by FC) generated by differential analysis of the proteome profiles between **(E)** OPN*^KO^* and **(F)** GA versus WT macrophages, and **(G)** GA versus OPN*^KO^* macrophages. Significantly upregulated proteins are shown as red dots and down-regulated proteins as green dots. The proteins with fold change >1.2 and p value <0.05 were considered significantly differentially expressed.

We previously found that GA stimulation upregulates OPN expression in BMMΦ, promoting an anti-inflammatory macrophage phenotype, whereas OPN deletion in BMMΦs results in pro-inflammatory phenotypes (6). We explored *in vitro* molecular network effects of OPN deficiency versus GA-mediated OPN induction in BMMΦ using applied MS-based quantitative proteomics profiling on protein extracts from OPN*^KO^* versus GA-stimulated BMMΦ compared to untreated wild type (WT) control BMMΦ. The scheme of the experimental procedure, which encompasses biochemical and MS analysis, data curation, and profiling followed by data validation, is outlined in **Figure 1A**. In brief, bone marrow was isolated from WT or OPN*^KO^* mice and cultured for 7 days in MCSF-enriched media to differentiate into macrophages. On day 6, one group of WT cells underwent overnight treatment with GA. On day 7, the cells were collected, pelleted, and processed for quantitative proteomic and bioinformatics analyses, and followed by immunocytochemical (ICC) and Western blot (WB) validation experiments.

Mass spectrometry analysis identified 2505 peptides corresponding to 631 DEPs amongst the three BMMΦ cell groups (**Table S2**). Of these, 249 proteins were significantly altered exceeding our 1.2-fold change cutoff (**Table 1**). In OPN-deficient (OPN*^KO^*) versus WT BMMΦ, 66 proteins were upregulated, while 16 were downregulated. In GA-treated compared to WT BMMΦ, 56 proteins were upregulated, and only 2 proteins were significantly downregulated. DEPs numbers were further enriched in comparing between GA-stimulated and OPN*^KO^* BMMΦ groups, in which 84 proteins were upregulated and 25 were downregulated (**Table 1**). A heatmap with hierarchical clustering analysis of the most abundant up (red)- and down (blue)-regulated DEPs across three experimental groups exhibited distinct cluster profiles for each group (**Figure 1B**). Prediction ellipses by principal component and Venn diagram analyses showed that each group has a discrete protein expression profile, and notably the OPN*^KO^* group had the least overlapping profile compared to the GA-treated BMMΦ group (**Figures 1C, D**).

**Table 1:** Mass spectrometry data showing significantly differentially expressed proteins (*P*<0.05).

OPN*^KO^*/WT			GA/WT			GA/OPN*^KO^*		
Symbol	FC	p-value	Symbol	FC	p-value	Symbol	FC	p-value
**SAP**	**1.60**	**0.005**	**RANG**	**1.45**	**0.014**	**UCHL1**	**3.28**	**4.94E-08**
**FABP5**	**1.59**	**0.000034**	**WASF2**	**1.40**	**0.016**	**TNNI3**	**2.11**	**0.0086**
**DX39B**	**1.58**	**0.036**	**MYOF**	**1.39**	**0.014**	**MP2K1**	**1.93**	**0.022**
**GILT**	**1.57**	**0.0045**	**PSA7**	**1.38**	**0.0054**	**SODC**	**1.67**	**0.031**
**PPT1**	**1.56**	**0.0026**	**RS28**	**1.37**	**0.0058**	**FPPS**	**1.64**	**0.0042**
**PRG2**	**1.52**	**0.00017**	**LMNB1**	**1.34**	**0.0022**	**RL36**	**1.59**	**0.0012**
**COX2**	**1.52**	**0.0049**	**SET**	**1.33**	**0.0064**	**SYIC**	**1.52**	**0.00011**
GRN	1.39	0.0026	**TEBP**	**1.33**	**0.0105**	**YBOX1**	**1.48**	**0.000083**
CATB	1.39	0.0023	GLRX1	1.32	0.0083	**ITA5**	**1.42**	**0.0026**
ASAH1	1.37	0.0024	IMB1	1.32	0.0027	**HMOX1**	**1.42**	**0.000042**
GPNMB	1.37	0.00014	SYRC	1.31	0.023	CNBP	1.42	0.0055
ECHA	1.37	0.017	SYEP	1.30	0.00013	RS2	1.35	0.0072
GALNS	1.37	0.047	COPD	1.29	0.026	PRP19	1.35	0.0074
LYZ2	1.36	0.00004	AN32B	1.29	0.039	PRS7	1.34	0.00015
M2OM	1.35	0.029	SSRD	1.29	0.019	RL3	1.33	0.0024
IDHP	1.34	0.00028	HMGA1	1.29	0.011	RL39	1.33	0.0037
ARF6	1.33	0.0057	TXD17	1.27	0.011	BACH	1.33	0.00014
HEXA	1.33	0.0012	PEBP1	1.27	0.03	RL27	1.33	0.0087
DPP2	1.33	0.00043	PKHO2	1.26	0.011	IF4B	1.32	0.0013
3HIDH	1.32	0.012	IDHP	1.26	0.00036	LMNB1	1.32	0.0005
CX6B1	1.31	0.0017	2AAA	1.26	0.000033	FUS	1.32	0.0029
ECHM	1.31	0.016	ATPD	1.25	0.045	PA2G4	1.32	0.0018
SHPS1	1.30	0.0015	CD36	1.25	0.037	RL21	1.32	0.049
ADT1	1.30	0.019	AL9A1	1.25	0.023	PRS10	1.32	0.016
ODPB	1.29	0.00042	MIF	1.24	0.012	RL13A	1.31	0.017
TWF1	1.29	0.015	ELAV1	1.24	0.034	DDX5	1.31	0.00098
EHD4	1.29	0.00084	MDHM	1.24	0.01	RS3A	1.31	0.00031
LAMP1	1.29	0.0096	SNAA	1.24	0.018	PTMA	1.30	0.032
GSTM1	1.28	0.00021	ITA5	1.24	0.031	RS25	1.30	0.0011
ANXA3	1.28	0.0011	ACTN4	1.23	0.019	TCTP	1.30	6.11E-06
ALDH2	1.28	0.0002	PSB3	1.23	0.0012	TCPE	1.30	0.0012
MDHM	1.28	0.0047	RINI	1.23	0.0075	HMGA2	1.29	0.014
CREG1	1.28	0.00072	GDIR2	1.23	0.0013	TBB6	1.29	0.0064
FUMH	1.28	0.00024	VATB2	1.22	0.01	ELAV1	1.29	0.0086
ARC1B	1.27	0.02043	VIME	1.22	0.0046	RS14	1.29	0.007
GNS	1.27	0.0013	TAGL2	1.22	0.006	MVP	1.29	0.00099
PPGB	1.27	0.0013	ECHM	1.22	0.049	RLA2	1.28	0.00017
SPB6	1.27	0.00873	FUMH	1.22	0.025	RS23	1.28	0.025
IDHC	1.27	0.00049	RLA0	1.22	0.0066	RLA1	1.28	0.0031
COTL1	1.27	0.00098	LSP1	1.22	0.0083	NLTP	1.28	0.0036
LEG1	1.25	0.0095	PSMD4	1.21	0.00001	RS17	1.28	0.0013
PKHO2	1.25	0.01	YBOX1	1.21	0.0072	PCNA	1.27	0.000054
VDAC1	1.25	0.003	CD68	1.21	0.0033	RS30	1.27	0.0041
CATZ	1.25	0.00062	SIAS	1.21	0.023	EIF3C	1.27	0.00097
HEXB	1.24	0.00016	ALDOA	1.21	0.00099	IMB1	1.27	0.002
EFTU	1.24	0.0037	NUCL	1.21	0.015	PCBP2	1.27	0.018
ETFA	1.24	0.0031	TCPZ	1.21	0.0039	PSB4	1.27	0.042
COPD	1.24	0.042	GARS	1.21	0.0306	SNAA	1.26	0.0043
ARL8B	1.24	0.014	1433G	1.21	0.013	RL31	1.26	0.012
ACON	1.23	0.013	DYHC1	1.21	0.027	ERF1	1.26	0.0018
ADT2	1.23	0.0041	ROA2	1.21	0.0043	GLU2B	1.26	0.0054
CAPZB	1.23	0.0089	PSA1	1.20	0.0068	GARS	1.26	0.046
PTGR1	1.23	0.0077	EFTU	1.20	0.0063	RS26	1.26	0.00009
ECHB	1.23	0.0057	PSA3	1.20	0.0134	SYAC	1.26	0.0061
ROA3	1.22	0.041	SYNC	1.20	0.013	RL23A	1.25	0.0021
ANXA6	1.22	0.034	TALDO	1.20	0.042	IF4A1	1.25	0.0014
ATPK	1.22	0.0038	**H3.2**	**-2.64**	**0.0097**	SH3L3	1.25	0.0018
SCOT1	1.22	0.041	**LRP1**	**-1.32**	**0.017**	RS12	1.24	0.00069
OPN*^KO^*/WT			GA/WT			GA/OPN*^KO^*		
Symbol	FC	p-value	Symbol	FC	p-value	Symbol	FC	p-value
RHOG	1.21	0.046				RL5	1.24	0.00035
LAMP2	1.21	0.0036				RS21	1.24	0.0029
G6PD1	1.21	0.032				PUR9	1.24	0.00051
CATD	1.21	0.00028				MBB1A	1.24	0.046
MTPN	1.21	0.025				RS18	1.23	0.013
HXK1	1.21	0.016				PCBP1	1.23	0.0011
ARPC3	1.20	0.0027				NUCL	1.23	0.00061
ETFB	1.20	0.000027				PLEC	1.23	0.000068
**UCHL1**	**-2.86**	**4.72E-06**				TCPZ	1.23	0.00017
**HMOX1**	**-1.53**	**0.000012**				RS8	1.22	0.000078
**FPPS**	**-1.45**	**0.021**				HMGA1	1.22	0.0059
HBA	-1.41	0.039				RSSA	1.22	3.36E-06
CNBP	-1.35	0.012				RL13	1.21	0.0039
AP2A2	-1.31	0.045				RS19	1.21	0.005
RL3	-1.30	0.0099				EF1B	1.21	0.0016
FAS	-1.26	0.037				EF2	1.21	0.00024
IF4B	-1.25	0.03				IF2A	1.21	0.0021
RL39	-1.23	0.039				SYEP	1.21	0.011
RL7A	-1.23	0.0064				LSP1	1.21	0.003
RL13A	-1.22	0.05				RACK1	1.21	0.0001
RL14	-1.22	0.035				TCPQ	1.21	0.0023
YBOX1	-1.22	0.0034				DYHC1	1.20	0.017
RL31	-1.21	0.008				RAN	1.20	0.0044
RL8	-1.19	0.035				FKB1A	1.20	0.0067
						EF1A1	1.20	0.00027
						SET	1.20	0.0045
						**H3.2**	**-2.41**	**0.016**
						**PRG2**	**-1.50**	**0.00061**
						**LYZ2**	**-1.45**	**2.0473E-07**
						**FABP5**	**-1.44**	**1.5067E-07**
						SHPS1	-1.43	0.00027
						**SDHA**	**-1.36**	**0.027**
						GPNMB	-1.35	0.000035
						GRN	-1.33	0.00024
						MRC1	-1.31	0.00011
						SAP	-1.30	0.0072
						BGLR	-1.29	0.013
						6PGL	-1.28	0.029
						GILT	-1.28	0.0046
						ASAH1	-1.27	0.0002
						PPT1	-1.27	0.038
						PPGB	-1.26	0.00076
						**ANXA6**	-1.24	0.043
						LRP1	-1.24	0.046
						GSTM1	-1.23	0.023
						HEXA	-1.22	0.00045
						ATP5J	-1.22	0.046
						DPP2	-1.21	0.0052
						SCOT1	-1.21	0.02
						**LAMP2**	-1.20	0.017
						COX6B1	-1.20	0.007

Bold values refer to the top up and down regulated DEPs by the |FC| values, described in the heatmaps and volcano plots of **Figures 1E–G**.

FC, Fold change.

### 3.2 Top DEPs in OPN-deficient macrophages

Among the top 10 DEPs in OPN*^KO^* vs. WT BMMΦ, we focused on the two most significantly downregulated proteins (**Figure 1E**). These include the ubiquitin carboxyl-terminal hydrolase isozyme 1 (UCHL1; 2.86-fold downregulation; **Table 1**), a deubiquitinating enzyme of the UPS system with an important role in maintaining ubiquitin homeostasis and protein synthesis turnover (34); Heme oxygenase 1 (HMOX1; 1.53-fold downregulation), a heme degrading enzyme that produces antioxidant and anti-inflammatory compounds (35). The Volcano plot (**Figure 1E**) highlights additional downregulated proteins that include the 60S ribosomal proteins RL3, cellular nucleic acid-binding protein (CNBP), and Farnesyl pyrophosphate synthase (FPPS). These data indicate that macrophages lacking OPN exhibit deficiencies in proteins involved in ubiquitin-proteosome system and translation.

Conversely, among the top 10 DEPs in OPN*^KO^* vs. WT BMMΦ, we detected seven upregulated proteins: including signaling lymphocytic activation molecule-associated protein (SAP), fatty acid-binding protein 5 (FABP5), DExD-Box Helicase 39B (DX39B), gamma-interferon-inducible lysosomal thiol reductase (GILT), palmitoyl-protein thioesterase 1 (PPT1), proteoglycan 2 (PRG2), and cyclooxygenase (COX2) (**Figure 1E**). These findings implicate the involvement of OPN in lysosomal organization, mitochondrial functions, oxidative stress, as well as metabolic and proteolytic responses in BMMΦ.

### 3.3 Top DEPs in GA-stimulated versus WT and OPN*^KO^* macrophages

In GA-treated macrophages, the top 10 DEPs highlight the histone H3.2 variant as markedly downregulated by 2.64- and 2.41-folds in GA-stimulated macrophages compared to WT and OPN*^KO^* macrophages, respectively (**Figures 1F, G** heatmaps, **Table 1**). The H3.2 histone protein is a DNA replication-dependent nucleosome assembly protein and associated with replication and/or cell division (36, 37). Volcano plot analysis further shows that the low-density lipoprotein receptor-related protein 1 (LRP1), which is involved in the positive regulation of lysosomal protein catabolic process and cell death (38, 39), was downregulated by GA treatment vs. WT controls (1.32-folds; **Table 1**, **Figure 1F**). These results suggest that GA can reverse detrimental lysosomal catabolism and cell death-mediated signaling. Additionally, two other proteins, Lyz2 (involved in cytolysis) and SDHA (a mitochondrial electron transport protein) were downregulated following GA stimulation compared to OPN*^KO^* BMMΦ (**Figure 1G**).

Notably, as described in **Table 1** and **Figure 1F, G** volcano plots, other important proteins were upregulated in macrophages by GA stimulation, including the scavenger receptors CD36 and CD68, which are involved in facilitating uptake and clearance of Aβ and were also previously shown to increase expression following GA stimulation (6, 19, 20).

Furthermore, several anti-inflammatory and antioxidant proteins were upregulated by GA treatments. These include UCHL1, superoxide dismutase C (SODC), HMOX1, troponin I3 (TNNI3), Y-box binding protein 1 (YBox1), which negatively regulates of apoptotic processes and promotes cell proliferation, and ribosomal proteins like RL36 (**Figures 1F, G**, **Table 1**). Importantly, the GA-influenced topmost upregulated proteins were anti-inflammatory and antioxidant-related proteins (**Table 1**). Taken together, GA-immunomodulation downregulated lysosomal and apoptotic cell death-related proteins while upregulating anti-inflammatory and antioxidant proteins, at least partially through increased OPN production in macrophages.

### 3.4 Functional and canonical pathways in OPN-deficient and GA-stimulated macrophages

**Figure 2:**
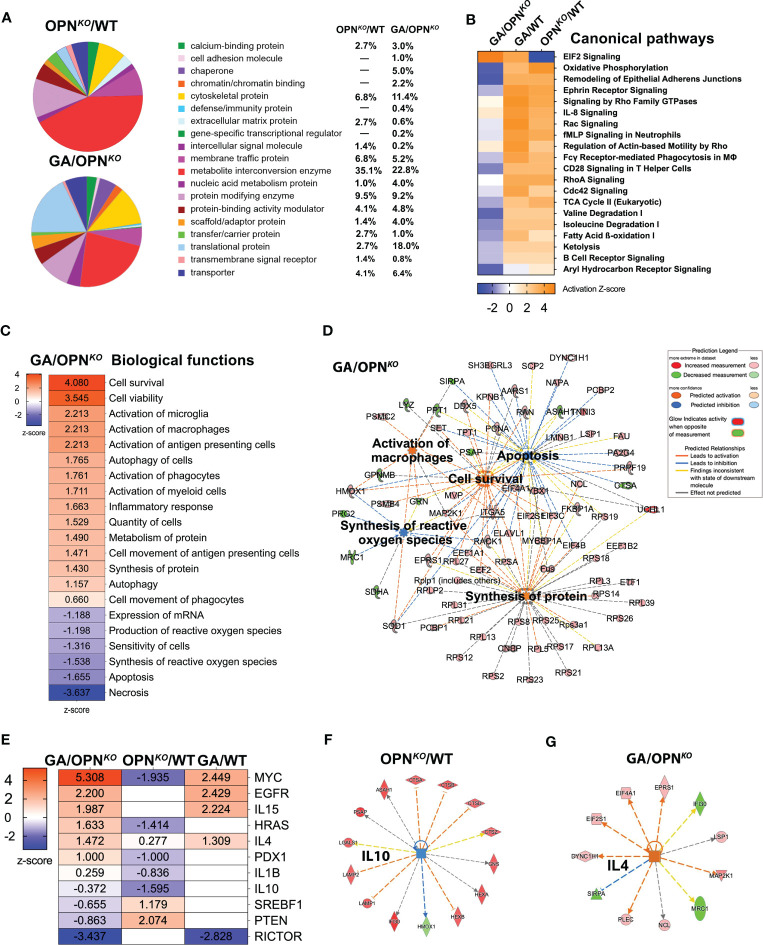
Functional and canonical pathways in OPN-deficient and GA-stimulated macrophages. **(A)** PANTHER analysis Pie chart by Protein Class distribution in percentages showing fraction and percentage of significant DEPs (up- or downregulated proteins) grouped by protein class category in the OPN*^KO^*/WT and GA/OPN*^KO^*. **(B)** IPA was performed to identify canonical pathways, most differentially regulated pathways in all three groups. **(C)** IPA of top up and down-regulated biological functions in GA/OPN*^KO^* group and **(D)** network displaying five important biological functions and their interactions with each other. **(E)** IPA of upstream regulator analysis in all three groups and network displays of upstream regulators for **(F)** IL-10 in OPN*^KO^*/WT and **(G)** IL4 in GA/OPN*^KO^*.

What are the leading biological pathways that OPN deficiency triggers in macrophages? To address this question, we studied the relative distribution of functionally classified DEPs in OPN-deficient and GA-immunomodulated BMMΦ. Pie charts based on the PANTHER functional classification, display the proportions between the 19 PANTHER clusters for each OPN*^KO^* vs. WT as well as the GA vs. OPN*^KO^* BMMΦ (**Figure 2A**). When we compared these 2 pie charts, the most substantial difference occurred in the ‘translational protein’ class with 6.6-fold differences, upregulated from 2.7% in OPN*^KO^* to 18% in GA-treated BMMΦ. While the most notable protein cluster changes in OPN*^KO^* BMMΦ were related to metabolic and protein modifying enzymes, in GA-treated BMMΦ the prominent clusters were related to metabolites, translational and cytoskeletal proteins. Further comparison between the pie clusters of GA vs. OPN*^KO^* BMMΦ revealed more clusters related to defense immunity, chromatin binding, and chaperone and cell adhesion class proteins; while fewer clusters were related to intercellular signaling, extracellular matrix, and transmembrane signal receptor following GA treatment in macrophages (**Figure 2A**).

Next, ingenuity pathway analysis (IPA) identified the principal canonical pathways and downstream signaling in OPN-depleted and GA-stimulated macrophages (**Figure 2B**). A top altered pathway was inhibited in OPN*^KO^* BMMΦ while activated in GA-stimulated, the EIF2 signaling (**Figures 2B**, **S1**); which is essential for most forms of translation initiation and protein synthesis (40–42). Another top altered pathway that was activated in OPN*^KO^* and conversely inhibited in GA-stimulated BMMΦ was related to oxidative phosphorylation (**Figures 2B**, **S2**). Oxidative phosphorylation is a metabolic pathway in mitochondria that is associated with cellular proliferation (43). Protein Interaction Network Extractor (PINE) analysis (**Figure S3**) displays the top five canonical ontology networks that were identified across the experimental macrophage groups. In alignment with our aforementioned results, the most significantly altered signaling pathways were related to the innate immune system, translation, and translation initiation.

To gain a deeper understanding of the biological pathways that are altered following GA stimulation in macrophages, we conducted IPA for GA vs. OPN*^KO^* BMMΦ (**Figures 2C, D**). The top activated biological pathways were related to cell survival, cell viability (Z scores = 3.54–4.08), and microglia/macrophage activation, cell movement, and protein synthesis (Z scores = 2.21–1.40). In contrast, the top inhibited functions were necrosis (Z-score = -3.637), apoptosis (Z-score = - 1.655), and synthesis of reactive oxygen species (ROS; Z score = -1.538) (**Figure 2C**). The top interconnecting molecular networks are displayed in **Figure 2D**. Opposite IPA functional directions of inhibited cell survival, viability, and macrophage activation and activation of necrotic, apoptotic, and ROS markers were detected for OPN*^KO^* vs. GA-treated BMMΦ (not shown). These results were further validated by IPA and gene ontology (GO) analyses (**Tables 2**, **3**). Next, we identified the specific upstream regulators across all three comparison groups (**Figures 2E–G**). Previous literature supports IL-4 as one of the top upstream activated regulators in GA-treated BMMΦ (Z-score = 1.472), promoting an anti-inflammatory phenotype in macrophages (24). IL-10 is another anti-inflammatory cytokine that was a top inhibited upstream regulator of OPN*^KO^* BMMΦ (Z-score = -1.595), which supports a pro-inflammatory phenotype for OPN-deficient macrophages.

**Table 2:** Disease or Functions by IPA analysis.

Disease or Functions: OPN*^KO^*/WT	p-Value	Activation State	Activation z-score	# of Proteins
Movement Disorders	9.31E-28	Decreased	-2.717	130
Lysosomal storage disease	1.29E-11	Decreased	-2.169	20
Cell death	1.47E-36	Increased	3.237	88
Endocytosis	4.21E-32	Increased	2.482	91
Polymerization of protein	2.41E-14	Increased	2.128	53
Chemotaxis	6.11E-10	Increased	2.153	55
Clathrin mediated endocytosis	1.38E-07	Increased	2.105	13
Disease or Functions: GA/WT	p-Value	Activation State	Activation z-score	# of Proteins
Necrosis	2.10E-63	Decreased	-5.979	298
Apoptosis	2.6E-36	Decreased	-4.074	248
Cell death	7.67E-28	Decreased	-4.255	165
Translation of mRNA	3.52E-17	Decreased	-2.41	35
Reactive oxygen species	0.00000123	Decreased	-2.62	22
Metabolism of protein	4.87E-64	Increased	3.052	186
Synthesis of protein	1.25E-60	Increased	3.61	119
Endocytosis	3.26E-32	Increased	4.679	91
Engulfment of cells	1.07E-27	Increased	4.435	76
Cell movement	4.4E-24	Increased	6.46	201
Phagocytosis	7.72E-21	Increased	3.459	55
Cell survival	2.89E-19	Increased	5.592	139
Migration of cells	5.32E-18	Increased	6.314	171
Transport of molecule	4.69E-17	Increased	2.164	141
Cell viability	5.47E-16	Increased	5.247	126
Organization of cytoskeleton	1.92E-15	Increased	4.294	125
Cellular homeostasis	1.71E-09	Increased	3.413	119
Proliferation of immune cells	8.12E-08	Increased	3.843	64
Disease or Functions: GA/OPN*^KO^*	p-Value	Activation State	Activation z-score	# of Proteins
Necrosis	2.1E-63	Decreased	-5.014	298
Apoptosis	2.6E-36	Decreased	-2.71	248
Cell death	7.67E-28	Decreased	-3.706	165
Reactive oxygen species	0.00000123	Decreased	-2.268	22
Translation of mRNA	3.52E-17	Decreased	-2.41	35
Cell survival	2.89E-19	Increased	4.776	139
Cell viability	5.47E-16	Increased	4.579	126
Synthesis of protein	1.25E-60	Increased	2.193	119
Endocytosis	3.26E-32	Increased	2.934	91
Phagocytosis	7.72E-21	Increased	2.965	55
Migration of cells	5.32E-18	Increased	2.14	171
Transport of molecule	4.69E-17	Increased	3.085	141

IPA, Ingenuity pathway analysis.

**Table 3:** Top 10 significant GO Biological processes identified for proteins differentially expressed (FDR<0.05, FC>1.2).

GO Biological Process OPN*^KO^*/WT	P-Value	Count
Metabolic process	6.90E-07	14
Translation	9.90E-07	13
Oxidation-reduction process	1.70E-06	16
ATP synthesis coupled proton transport	5.40E-06	5
Tricarboxylic acid cycle	1.40E-05	5
Lipid metabolic process	1.20E-04	11
Proton transport	2.20E-04	5
Glycosaminoglycan metabolic process	3.90E-04	3
Isocitrate metabolic process	3.90E-04	3
Lysosome organization	1.10E-03	4
GO Biological Process: GA/WT	P-Value	Count
Cell-cell adhesion	2.30E-07	9
Actin cytoskeleton organization	1.20E-04	6
Protein targeting	2.80E-04	4
Sequestering of actin monomers	7.20E-04	3
Antigen processing and presentation of exogenous peptide antigen *via* MHC class I, TAP-dependent	4.60E-03	3
Cytoskeleton organization	5.20E-03	4
Nucleosome assembly	5.20E-03	4
tRNA aminoacylation for protein translation	6.60E-03	3
Substantia nigra development	7.30E-03	3
Protein heterotetramerization	1.10E-02	3
GO Biological Process GA/OPN*^KO^*	P-Value	Count
Translation	1.20E-37	40
Ribosomal small subunit assembly	1.10E-07	6
Cytoplasmic translation	1.50E-06	6
Cell-cell adhesion	1.60E-06	10
Translational elongation	1.60E-04	5
Ribosomal small subunit biogenesis	1.70E-04	4
Cellular response to interleukin-4	5.00E-04	4
Maturation of SSU-rRNA from tricistronic rRNA transcript (SSU-rRNA, 5.8S rRNA, LSU-rRNA)	5.60E-04	4
Regulation of translation	7.00E-04	6
rRNA processing	8.40E-04	6

GO, Gene ontology; FDR, False discovery rate; FC, Fold change.

Overall, the combined canonical pathway and network analyses suggests that proteins associated with translation, protein synthesis, metabolism, and apoptosis are oppositely regulated in OPN-deficient and GA-stimulated macrophages, emphasizing their role in cellular homeostasis.

### 3.5 OPN interacts with UCHL1 and regulates its expression in BM-derived macrophages

**Figure 3:**
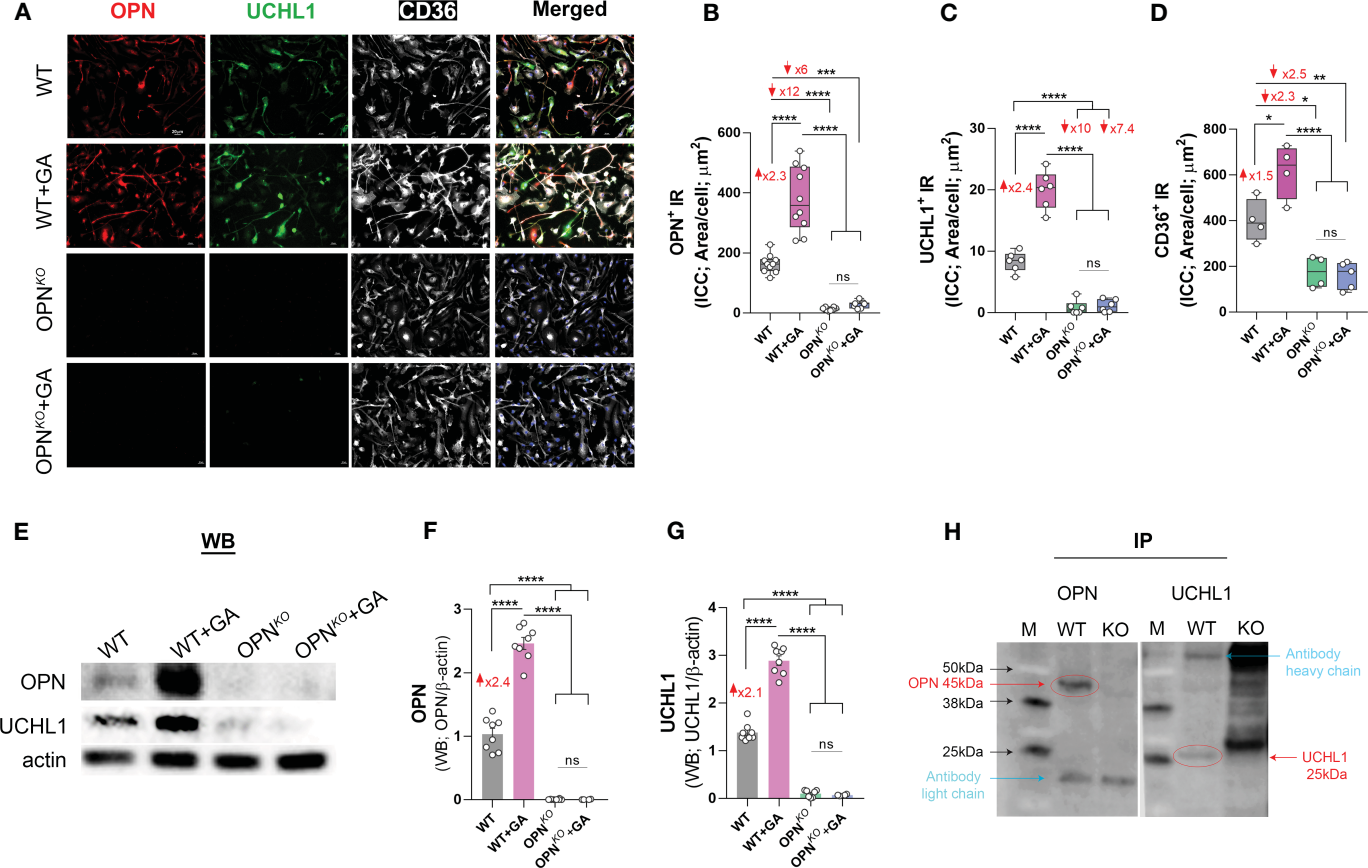
OPN interacts with UCHL1 and regulates its expression in BM-derived macrophages. **(A)** Representative fluorescent micrographs of BMMΦ cells, either GA-treated or untreated OPN*^KO^* or WT, immunostained for OPN (red), UCHL1 (green), CD36 (white) and DAPI (blue for nuclei of cells); scale bar: 20µm. Quantitative analysis of immunoreactive (IR) area of **(B)** OPN, **(C)** UCHL1 and **(D)** CD36 expression in BMMΦs. **(E)** Western blot (WB) analysis of cell lysates 24 hours after treatment, and quantitative analysis of **(F)** OPN and **(G)** UCHL1 bands. **(H)** Immunoprecipitation (IP) of OPN and UCHL1. IP demonstrates that OPN and UCHL1 interact with each other in protein complex. Individual data points, group means, and SEMs are shown. ns means Not Significant, *p < 0.05, **p < 0.01, ***p < 0.001, ****p < 0.0001 by one-way ANOVA with Tukey’s *post-hoc* multiple comparison test.

Our proteomics data identified UCHL1 as the most downregulated protein in OPN-deficient BMMΦ (**Figure 1E**, **Table 1**) and the most upregulated protein upon GA stimulation compared to OPN*^KO^* macrophages (**Figure 1G**, **Table 1**). We confirmed these findings by immunocytochemistry and Western blot analyses (**Figures 3A–G**, **S4A–D**). These studies revealed that UCHL1 was expressed by CD36-positive BMMΦ cells, and concomitant with enhanced OPN expression, UCHL1 was 2.4-fold upregulated following GA stimulation for 24 hours (**Figures 3A–G**). On the other hand, along with OPN deficiency in macrophages, UCHL1 expression was nearly absent in OPN*^KO^* BMMΦ cultures. Notably, GA-stimulation of OPN*^KO^* macrophages did not modify neither OPN nor UCHL1 expression, demonstrating that UCHL1 upregulation by GA is OPN dependent. Additionally, we validated these findings by inhibiting OPN expression using siRNA^OPN^ or minocycline (**Figures S4B–D**), further suggesting that OPN regulates UCHL1 expression in macrophages even after 2 hours of GA stimulation.

To explore a possible interaction between OPN and UCHL1 proteins, we performed an immunoprecipitation (IP) and found that both OPN and UCHL1 were pulled down in the same protein complex (**Figure 3H**). These results suggest that OPN and UCHL1 may directly interact with each other in a protein complex. In conclusion, we identified UCHL1 expression in BMMΦ and found that UCHL1 expression is OPN-dependent.

### 3.6 OPN deficiency upregulates lysosomal and mitochondrial markers in macrophages

**Figure 4:**
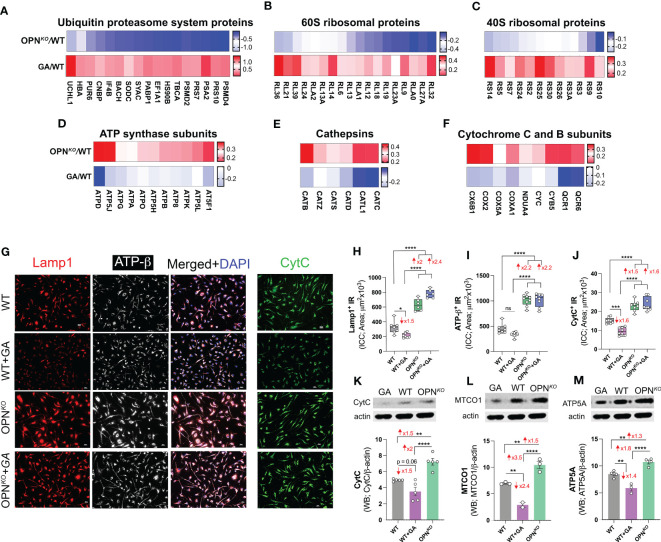
OPN deficiency upregulates lysosomal and mitochondrial markers in macrophages. **(A–F)** Heatmaps of **(A)** UPS proteins, **(B)** 60S ribosomal proteins, **(C)** 40S ribosomal proteins in GA/WT vs. OPN*^KO^*/WT, **(D)** ATP-synthase subunits, **(E)** cathepsins and **(F)** cytochrome C and B subunits in OPN*^KO^*/WT vs. GA/WT. **(G)** Representative fluorescent images of BMMΦ cells, either GA-treated or untreated OPN*^KO^* or WT, immunostained for Lamp1^+^ (red), ATP-β^+^ (white) and Cytochrome C^+^ (green); scale bar: 20µm. **(H–J)** Quantitative analysis of IR of **(H)** Lamp1, **(I)** ATP-β and **(J)** CytC expression in BMMΦs. **(K–M)** Western blot assay and densitometric analysis of cell lysates using CytC and OXPHOS antibody cocktail containing five mouse antibodies against: CI subunit NDUFB8, CII SDHB, CIII-Core protein 2 UQCRC2, CIV subunit I MTCO1 and CV alpha subunit ATP5A. Full gel is displayed in **Figure S4E**. Densitometric analysis of **(K)** CytC, **(L)** MTCO1 and **(M)** ATP5A bands. Individual data points, group means, and SEMs are shown. *p < 0.05, **p < 0.01, ***p < 0.001, ****p < 0.0001 by one-way ANOVA with Tukey’s *post-hoc* multiple comparison test.

Given that the proteomic analyses indicated marked changes in translation, lysosomal, and mitochondrial proteins in OPN*^KO^* and in GA-stimulated BMMΦ (**Figures 4A–F**), we aimed to further verify this data by immunolabeling macrophages with antibodies against late lysosomal marker (Lamp1) and mitochondrial markers cytochrome C (CytC) and ATP-β (**Figure 4G**). ICC quantification in OPN*^KO^* BMMΦ showed significant increases in the expression of Lamp1 (2-fold), ATP-β (2.2-fold), and CytC (1.5-fold; **Figures 4H–J**). Conversely, their expressions were downregulated in GA-treated BMMΦ. These results were further confirmed *via* Western blot assays. Quantitation of WB bands, including those from the oxidative phosphorylation (OXPHOS) mitochondrial-targeted cocktail containing five antibodies, revealed 1.3-1.5-fold increases in CytC, mitochondrial encoded cytochrome c oxidase I (MTCO1), and ATP synthase complex V (ATP5A) expressions in the OPN*^KO^* macrophages, while they were 1.4-2.4-fold reduced in the GA-stimulated macrophages (**Figures 4K–M**; the OXPHOS gel is displayed in **Figure S4E**).

Collectively, OPN deficiency was associated with lysosomal and mitochondrial imbalance in BMMΦ, suggesting a role for OPN-primed UCHL1 in regulating mitochondrial functions and cellular homeostasis. Combined with our bioinformatics analysis (**Figure S2**), we revealed that this dual OPN-UCHL1 deficiency is associated with oxidative phosphorylation and mitochondrial dysfunctions in macrophages.

### 3.7 Anti-inflammatory and antioxidant profile was upregulated with GA and downregulated in OPN-deficient BMMΦ

**Figure 5:**
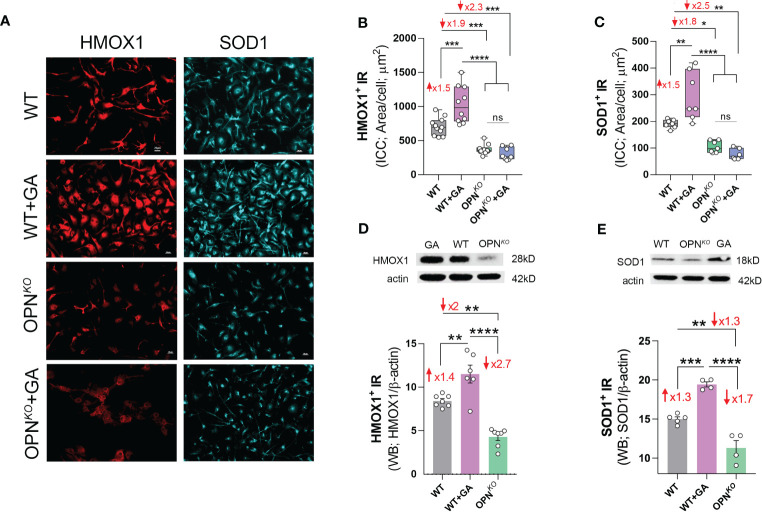
Anti-inflammatory and antioxidant enzymes were downregulated in OPN- deficient BMMФ. **(A)** Representative fluorescent micrographs of BMMΦ cells, either WT or OPN*^KO^* untreated or pretreated with GA, and immunostained for HMOX1^+^ (red), and SOD1^+^ (cyan); scale bar: 20µm. Quantitative analysis of ICC reveals increased level of **(B)** HMOX1 and **(C)** SOD1 following GA treatment, but reduced expression in OPN*^KO^* cells. Western blot and quantitative analysis of **(D)** HMOX1 and **(E)** SOD1 bands from cell pellets. Individual data points, group means, and SEMs are shown. *p < 0.05, **p < 0.01, ***p < 0.001, ****p < 0.0001 by one-way ANOVA with Tukey’s *post-hoc* multiple comparison test.

Given that OPN-primed UCHL1 expression was linked to cell homeostasis and protection, we assessed whether such protective effects in macrophages were, at least in part, associated with the expression of heme oxygenase 1 (HMOX1) and the superoxide dismutase (SODC, encoded by the SOD1 gene). In proteomics, we found that HMOX1 and SOD1 are two top upregulated DEPs in GA-stimulated vs. OPN*^KO^* BMMΦ, which were downregulated in OPN*^KO^* BMMΦ (**Figures 1G, E**, **Table 1**). HMOX1 is a heme degrading enzyme that catalyzes the production of antioxidant and anti-inflammatory compounds and provides beneficial effects for cellular homeostasis and switching macrophages to an anti-inflammatory phenotype (35, 44–46). HMOX1 deficiency renders macrophages sensitive to cell death. Our quantitative ICC analysis showed a substantial 1.9-fold reduction of HMOX1 in OPN*^KO^* vs. WT BMMΦ (**Figures 5A, B**). SOD1 is another potent antioxidant that has pleiotropic biological functions to counter oxidative stress in mitochondria (47, 48). We further reaffirmed the proteomics findings of SOD1 in OPN*^KO^* BMMΦ by employing ICC analysis (**Figures 5A, C**). Likewise, our WB analysis showed that the expression of these two antioxidant enzymes (SOD1 and HMOX1) were upregulated upon GA stimulation (1.3-1.4-fold) and decreased in OPN*^KO^* compared to WT BMMΦ (1.3- and 2-fold, respectively; **Figures 5D, E**).

### 3.8 OPN deficiency in macrophages induces ROS production, mitochondrial membrane damage, and subsequent apoptosis

**Figure 6:**
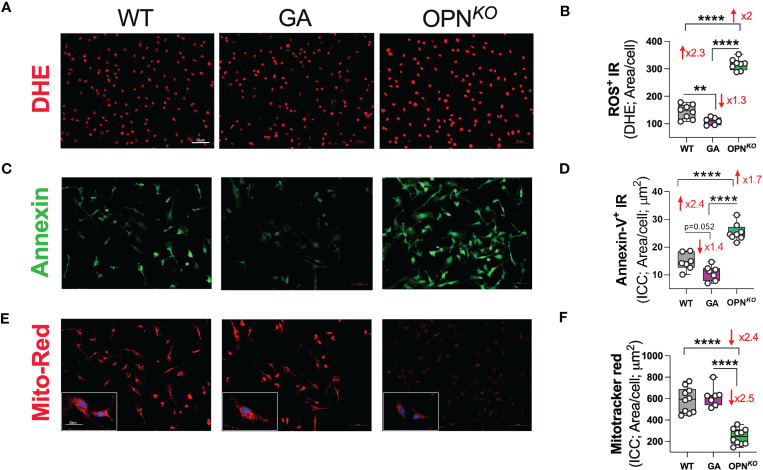
OPN deficiency induces ROS production and subsequent apoptosis in BMMФ. **(A, B)** Representative fluorescent micrographs of BMMΦ cells stained for DHE and quantitative analysis of ROS/DHE IR area in BMMΦ. **(C, D)** Representative fluorescent micrographs of BMMΦ cells immune-stained for Annexin-V and quantitative analysis of Annexin-V IR area in BMMΦ. **(E, F)** Representative fluorescent micrographs of BMMΦ cells immune-stained for Mitotracker-red and quantitative analysis of Mitotracker-red IR area in BMMΦ. Scale bar: 50µm. High power (x40) image inserts are shown. Scale bar: 20µm. Individual data points, group means, and SEMs are shown. **p < 0.01, and ****p < 0.0001 by one-way ANOVA with Tukey’s *post-hoc* multiple comparison test.

We sought to determine whether GA stimulation vs. OPN deficiency in macrophages leads to decreased oxidative stress. Indeed, IPA revealed that GA-stimulated vs. OPN-deficient BMMΦ had inhibited the synthesis and production of ROS as well as reduced necrosis and apoptosis pathways (**Figures 2C, D**). To directly evaluate ROS production, we further employed fluorescence dye-based detection method using dihydroethidium (DHE) staining. As shown in **Figure 6A**, ROS accumulation was much stronger in OPN*^KO^* than in WT BMMΦ. This effect was reversed upon GA treatment. Quantitative analysis of ROS (DHE)-immunoreactive area (**Figure 6B**) showed a 2-fold increase in ROS levels in OPN*^KO^* BMMΦ, whereas GA treatment reduced it by 1.3-fold. Additionally, ICC labeling with annexin-V antibody (an apoptotic marker), demonstrated 1.7-fold increase in OPN-deficient BMMΦ that was 1.4-fold decreased following GA stimulation (**Figures 6C, D**).

Finally, to determine levels of mitochondrial membrane potential (MMP) in live BMMΦ, we employed Mitotracker-red assay. The MMP of healthy cells is relatively high and shows a red fluorescence, while MMP of apoptotic cells is decreased and shows a diminished fluorescence (**Figures 6E, F**). The red fluorescence of GA-treated cells did not significantly change, implying preserved mitochondrial membrane potential after GA treatment. By contrast, in OPN*^KO^* versus WT macrophages, the ratio of red fluorescence was diminished by 2.4-fold, indicating impaired mitochondrial membranes in OPN*^KO^* cells that undergo apoptosis. In conclusion, OPN deficiency in macrophages induces ROS production, mitochondrial membrane potential damage, and subsequent apoptosis. In contrast, GA treatment decreases ROS and preserves MMP integrity.

**Figure 7:**
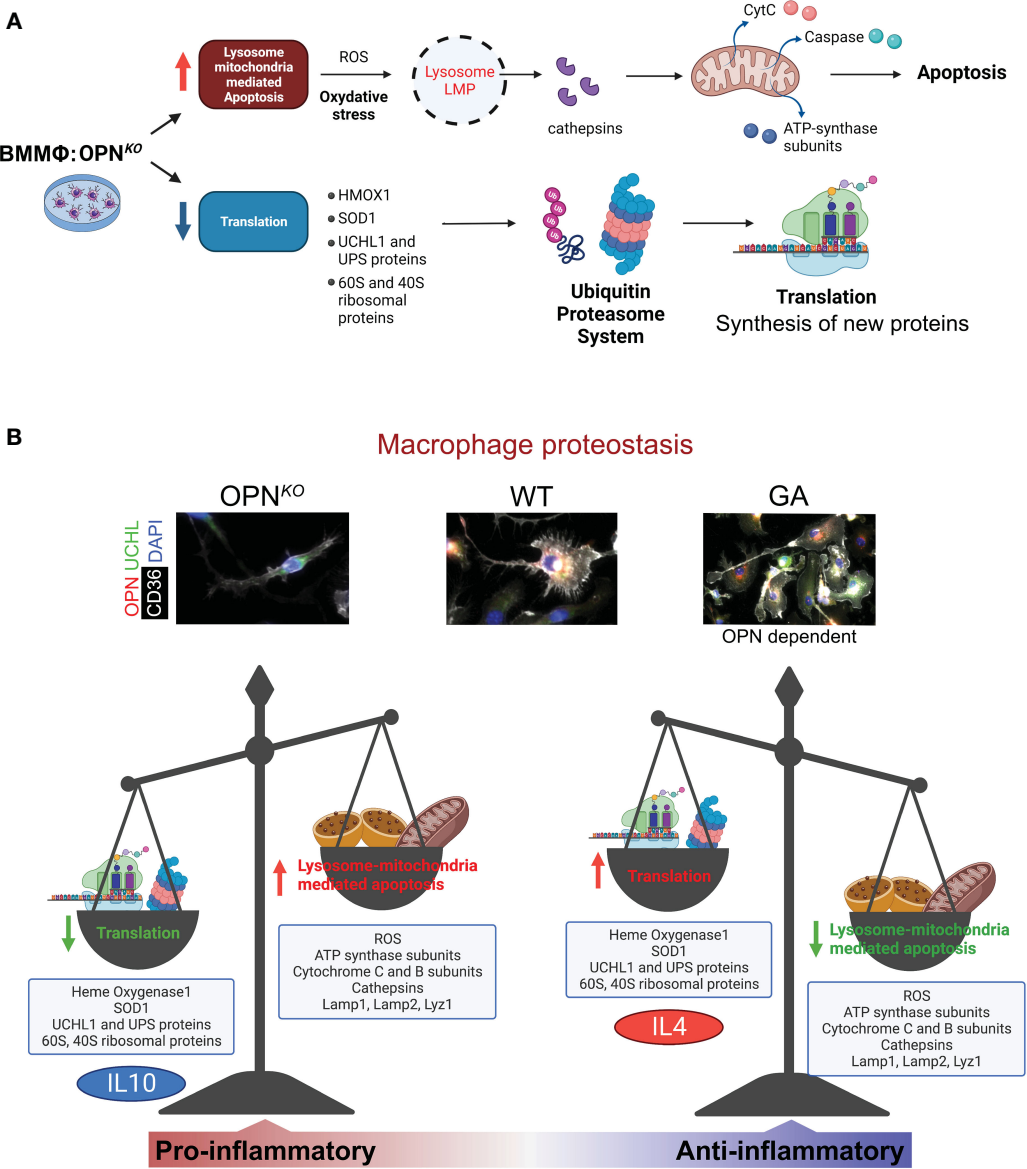
OPN/Spp1 deficiency in BMMФ triggers lysosome-mitochondria-mediated apoptosis accompanied by reduced translation and recycling. **(A)** Illustration summarizing lysosome-mitochondria-mediated apoptosis accompanied by reduced translation and UPS recycling in OPN-deficient macrophages. **(B)** Illustration of macrophage proteostasis emphasizing up- and downregulated proteins/groups and functions in OPN*^KO^* vs GA-stimulated macrophages *in vitro*. Illustration made with BioRender.com.

**Figures 7A, B** summarizes the effects of OPN-deficiency compared to GA-stimulation on the phenotypes of BMMΦ. We found that OPN deficiency perturbs cellular homeostasis in macrophages through increased ROS production, and lysosome-mitochondria-mediated apoptosis, whereas OPN-primed GA immunomodulation restores cellular homeostasis by markedly increasing UCHL1 expression, inducing anti-inflammatory phenotype and cell survival, reducing ROS production, and mitochondrial dysfunction.

## 4 Discussion

In this study, we identified a novel role for OPN in macrophages related to UPS and proteostasis. We employed both OPN-deletion loss-of-function and GA-stimulated OPN gain-of-function *in vitro* paradigms. Proteomics profiling by mass spectrometry analysis detected 630 DEPs when comparing between OPN*^KO^*, GA drug-stimulated, and WT macrophages. OPN deletion in macrophages induced activation of pro-inflammatory, mitochondrial dysfunction, apoptotic, and lysosomal signaling-related pathways, while inhibiting UPS, translation, and antioxidant-related processes, resulting in disrupted cellular homeostasis. Notably, GA stimulation reversed most of these adverse pathways through induction of OPN expression in macrophages, thus restoring an anti-inflammatory phenotype and macrophage proteostasis. These findings were further confirmed *via* Western blot and immunocytochemistry analyses, as well as immunoprecipitation and mitochondrial tracing assays. Overall, this study provides substantial evidence for the essential role of OPN in maintaining macrophage homeostasis.

One of the most notable findings of this study is the direct connection between OPN and UCHL1 in macrophages. UCHL1 was the most significantly downregulated protein in OPN-deficient macrophages. Surprisingly, while UCHL1 is recognized as a neuronal-specific protein (49–51), we found that UCHL1 was expressed by macrophages. Importantly, our finding that UCHL1 was downregulated upon OPN deficiency and upregulated by OPN enhancement through GA stimulation indicates that UCHL1 expression is OPN-dependent. Moreover, we show that OPN interacts with UCHL1 in protein complex by co-immunoprecipitation, further affirming that UCHL1 expression is tightly regulated by OPN. Two recent studies indicating that OPN and UCHL1 were intracellularly localized to mitochondria (52, 53), align with our results, suggesting a potential interaction in the mitochondria to regulate oxidative reactions and maintain mitochondrial function.

UCHL1 is a deubiquitinating enzyme (DUB), a key cellular proteolytic enzyme responsible for the removal of oxidized and/or damaged proteins, and a prominent component of the UPS (54, 55). The UPS plays a key role in preserving cellular homeostasis through the regulation of key cellular processes such as transcription, protein quality control and degradation, cell stress responses, cell cycle progression, and apoptosis (55, 56); such pleotropic cellular functions make UPS a central regulator of diverse cellular processes. A recent study showed that DUBs inhibition in macrophages resulted in acute perturbation of cellular ubiquitin homeostasis and induction of oxidative stress through ROS (57). This indicates that DUBs are responsible for mitigating oxidative damage and maintaining cellular homeostasis in macrophages. Furthermore, crosstalk between the UPS and mitochondrial proteins are reported to play a significant role in cellular homeostasis (58–60).

Indeed, a lower level of UCHL1 has been detected in apoptotic cells with severe loss of the mitochondrial membrane potential (61). Another report found that UCHL1 inhibition increased neuronal and apoptotic cell death (62) and UCHL1 deficiency in skeletal muscle cells resulted in altered mitochondrial oxidative phosphorylation (59). Accordingly, in the current study, we found that UCHL1 deficiency in OPN*^KO^* macrophages was associated with harmful cellular processes. These processes include lower expression of potent antioxidant enzymes, such as SOD1 and HMOX1 (47, 63), along with increased ROS production as well as, elevated lysosomal, mitochondrial, and apoptotic proteins as lysozyme C-2, ATP-synthase subunits, cathepsins, and cytochrome C and B subunits, and annexin-V. Moreover, there was also a severe loss of mitochondrial membrane potential measured in UCHL1-deficient OPN*^KO^* macrophages. Therefore, our data supports a role for the UCHL1-OPN axis in regulation of mitochondrial oxidative stress in macrophages.

A summary of our hypothesis on OPN-deficiency and OPN-induction effects in macrophages is illustrated in **Figure 7**. Following lysosomal membrane permeabilization (LMP), lysosomal proteases, such as cathepsins, are released into the cytosol and mediate cytochrome-C release and caspase activation. This ultimately triggers mitochondria-mediated apoptotic cascade (64). During apoptosis, caspase-dependent cleavage of translation-initiation factors leads to the inhibition of protein synthesis and translational shutdown (65). OPN also influences lysosomal protein degradation and protease activation as it triggers Lamp1 production. The current study reveals that loss of OPN resulted in reduced expression of UCHL1 that ultimately triggered apoptotic markers. As mentioned above, lower UCHL1 levels were detected in apoptotic cells with severe loss of mitochondrial membrane potential (61). The collective data clearly indicates that OPN-UCHL1 signaling can maintain cellular homeostasis by controlling apoptotic processes. We also found that OPN was involved in the regulation of protein synthesis and translation. Since UCHL1 expression in macrophages is found to be regulated by OPN, among other UPS proteins, OPN has a crucial role in protein translation and turnover, and cellular homeostasis. This result is supported by a previous report that found OPN to regulate the homeostasis and function of natural killer cells (66).

Our data also revealed that OPN deficient macrophages were exhibiting a pro-inflammatory phenotype. In fact, our data indicates that OPN may inhibit inducible nitric oxide synthase, thus, inhibiting nitric oxide production in macrophages leading to suppressed oxidative stress and inflammation (67). Indeed, we recently reported the potential anti-inflammatory effects of OPN as it can resolve inflammatory cascades through promoting macrophages polarization towards their anti-inflammatory and highly phagocytic phenotypes (6). Similarly, anti-apoptotic functions of OPN have been previously reported as inhibiting OPN by monoclonal antibody and dramatically promoted the apoptosis of activated T cells (68). These independent studies suggest the antioxidant, anti-inflammatory, and anti-apoptotic effects of OPN in different immune cells.

This study highlights the role of OPN in immune modulation of macrophage phenotype. Previous studies showed that OPN interacts with immune cells and extracellular matrix (ECM) proteins through adhesive integrin binding motifs: the adhesive RGD domain (binding to α_v_-containing integrins and α_5_β_1_ integrins) and the SLAYGLR sequence (binding to α_4_β_1_, α_4_β_7_ and α_9_β_1_ integrins) (69). Notably, our macrophage proteome data indicated that out of 249 identified DEPs only ITA5, an integrin α_5_ coded by the ITGA5 gene (shown in **Table 1**), was significantly upregulated by GA stimulation (1.24-fold change, p = 0.031) and further upregulated as compared with OPN*^KO^* macrophages (1.42-fold change, p = 0.0026). GO analysis showed activation of cell to cell adhesion by GA (**Table 3**). Also, in IPA functional analysis, the top interconnecting molecular networks predicted ITGA5 activation in GA-stimulated versus OPN*^KO^* macrophages with direct involvement in macrophage activation (**Figure 2D**, underlined). OPN has been known to interact with the cell surface adhesion receptor CD44 in RGD in an independent manner (4, 5), however, our proteome profiling showed only marginal downregulation of CD44 in OPN*^KO^* macrophages (**Table s2**). Interestingly, we observed that OPN*^KO^* macrophages have some tendency to detach from the surface by day 7-8 after culturing, possibly due to lack of OPN’s adhesive integrin binding domains. Following human recombinant OPN supplement (data not shown), the OPN*^KO^* macrophages behaved almost like the WT macrophages, restoring their surface adhesive propensity. These findings suggest that OPN impacts macrophage inflammatory phenotype and adhesive properties through its integrin binding sites. Future studies should investigate the molecular mechanisms that governs OPN-dependent effects on macrophage inflammatory and surface adhesion phenotype.

Importantly, our current study also revealed the key OPN-associated functions of GA, as GA treatment not only increased the OPN production in macrophages, but it also reversed the expression of almost all proteins influenced by the OPN deficiency. Furthermore, GA-stimulation of OPN*^KO^* macrophages did not change both OPN and UCHL1 expression, demonstrating that GA effects on macrophage profile is OPN dependent. As an immunomodulator, GA has been reported to influence pleiotropic cellular functions in multiple immune cells such as macrophages, microglia, and T cells both *in vivo* and *in vitro* disease models of neurodegenerative disorders including AD. We previously reported that administration of GA to transgenic murine models of AD enhanced brain recruitment of blood-borne monocytes, which were involved in the clearance and degradation of Aβ plaques, reduced neuroinflammation, and induced production of neurotrophic factors. This resulted in significant neuroprotection and cognitive improvements (19–22, 70–76). In addition, GA-primed neuroprotection against the pathology associated with AD in transgenic mice model was linked with the induction of a phenotypic shift in microglia, which typically expressed IGF-1. Such microglia were involved in the phagocytosis of cerebral Aβ plaques and regulating detrimental chronic inflammation (24). These effects of GA on immune cells leading to neuroprotection against AD pathogenesis is, at least in part, associated with increased production of OPN in immune cells influencing their anti-inflammatory and neuroprotective functions. Furthermore, a recent report demonstrated that OPN promoted neuroprotection by inhibiting NLRP3 inflammasome and inflammatory microglial activation following focal ischemic brain injury in mice as well as LPS-stimulated rat primary microglia (77), further affirming the strong anti-inflammatory potential of OPN.

Although this study has many strengths and provides new insights, we acknowledge a few limitations. The immunoprecipitation assay did not include the GA-stimulated macrophages. Hence, the effects of OPN deficiency and GA stimulation on UCHL1-OPN interaction in a protein complex need to be investigated under diverse macrophage activation states in future studies. Further research is warranted to determine the physiological role of OPN and UCHL1 in macrophages under both healthy and disease conditions

## 5 Conclusion

This study reveals that OPN deficiency in macrophages is associated with homeostatic imbalance by affecting multiple downstream signaling pathways such as UCHL1-UPS downregulation, ROS production, oxidative stress, mitochondrial-related dysfunctions, and subsequent apoptosis. Reciprocally, the induction of apoptosis leads to a substantial inhibition of protein synthesis and translation, which was a feature of OPN*^KO^* macrophages. On the other hand, GA appears to maintain cellular homeostasis through the induction of OPN production, which in turn regulates UPS, protein recycling, and protein synthesis. Our proteomics data in conjunction with bioinformatics analysis offer the molecular insights into phenotypic changes that occur in OPN-deficient and GA-stimulated macrophages. We revealed the importance of OPN modulation in cellular proteostasis and ROS production. On aggregate, this study indicates that targeting of UPS and lysosome-mitochondrial pathways may hold therapeutic potential for enhanced macrophage function and immunomodulation therapies.

## Data availability statement

The datasets presented in this study can be found in online repositories. The names of the repository/repositories and accession number(s) can be found below: MSV000091221(MassIVE) and Proteome Exchange ID: PXD039950.

## Ethics statement

The animal study was reviewed and approved by Cedars-Sinai Medical Center Institutional Animal Care and Use Committee (IACUC).

## Author contributions

AR, MKH: Study conception, design, and supervision. AR, DTF, JS: Major experimental contribution, planning, data acquisition and analysis. KR, VV, RP, AR, JS, JVE: Mass spectrometry and analysis. HS, JD, YK: additional experiments. AR, KR, DTF, JS, VV, RP, HS, JD, JDFG, YK, MA, KB, BPG, MKH: Data interpretation and edits. AR, DTF, BPG, MKH: Figure preparation, manuscript writing and editing, and revision. All authors approved the final version.
