# Supplementary material for: Osteopontin depletion in macrophages perturbs proteostasis via regulating UCHL1-UPS axis and mitochondria-mediated apoptosis
Source: Front Immunol. 2023 May 30;14:1155935. doi: 10.3389/fimmu.2023.1155935 (PMC10266348; doi:10.3389/fimmu.2023.1155935)
Supplement: Supplementary file 1 [file DataSheet_1.pdf]

Supplemental Figure S1. Canonical pathway analysis for EIF2 signaling in GA-stimulated vs OPN^{KO} macrophages.

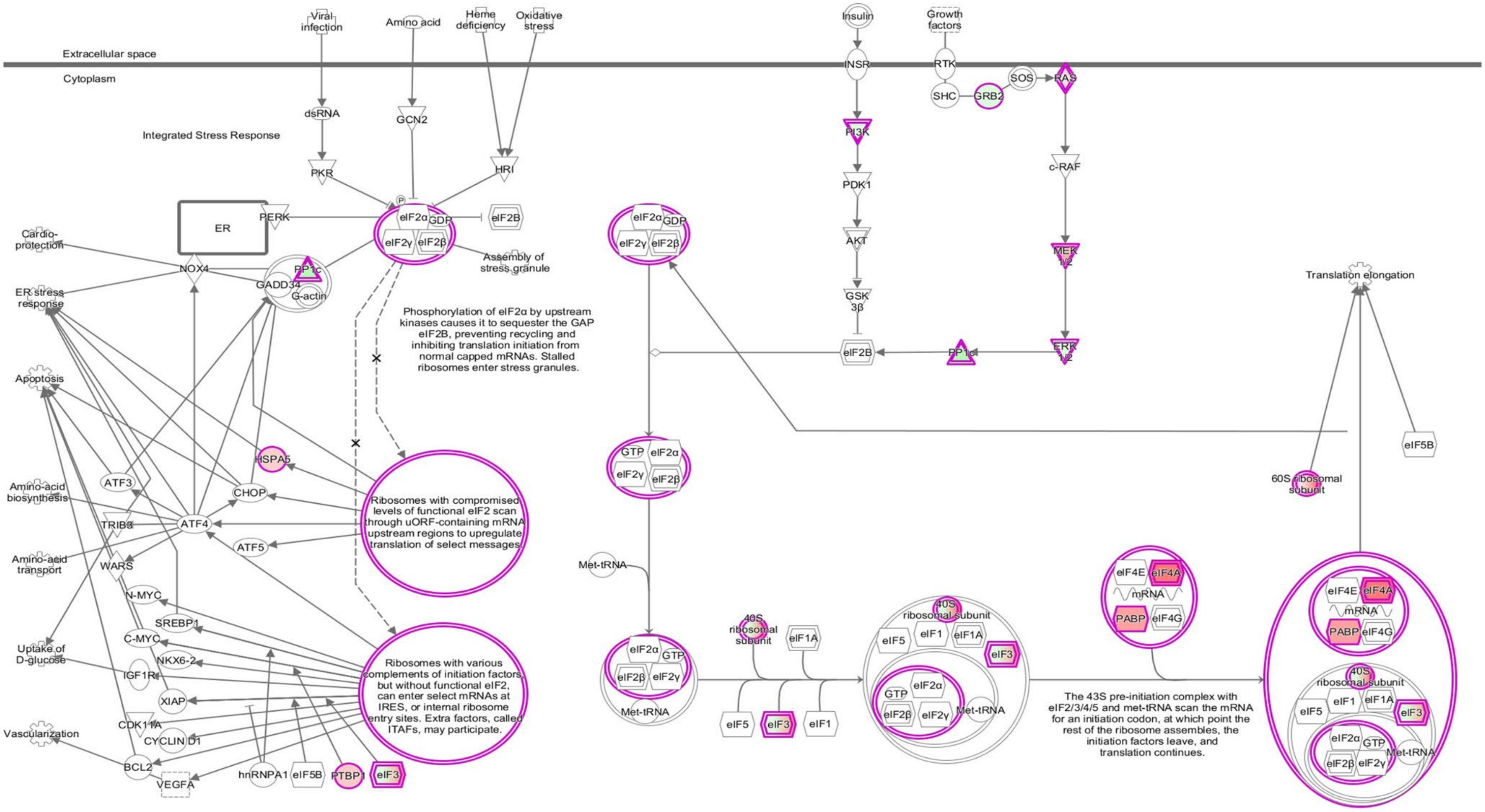

Supplemental Figure S2. Canonical pathway analysis for mitochondrial dysfunction and oxidative phosphorylation in GA-stimulated vs OPN^{KO} macrophages.

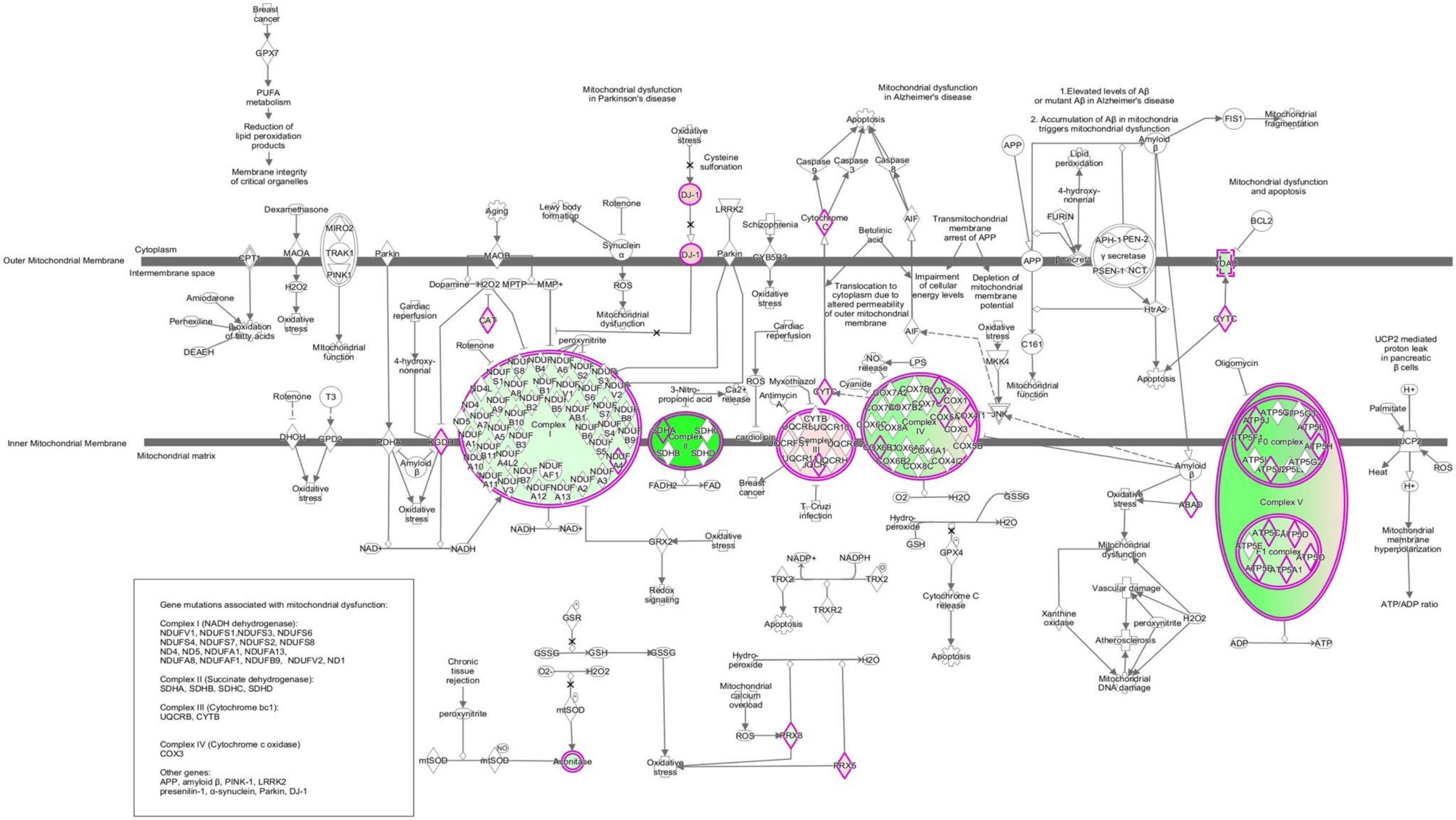

Supplemental Figure S3. Functional pathway analysis of BM-derived OPN-deficient and GA-immunomodulated macrophages.

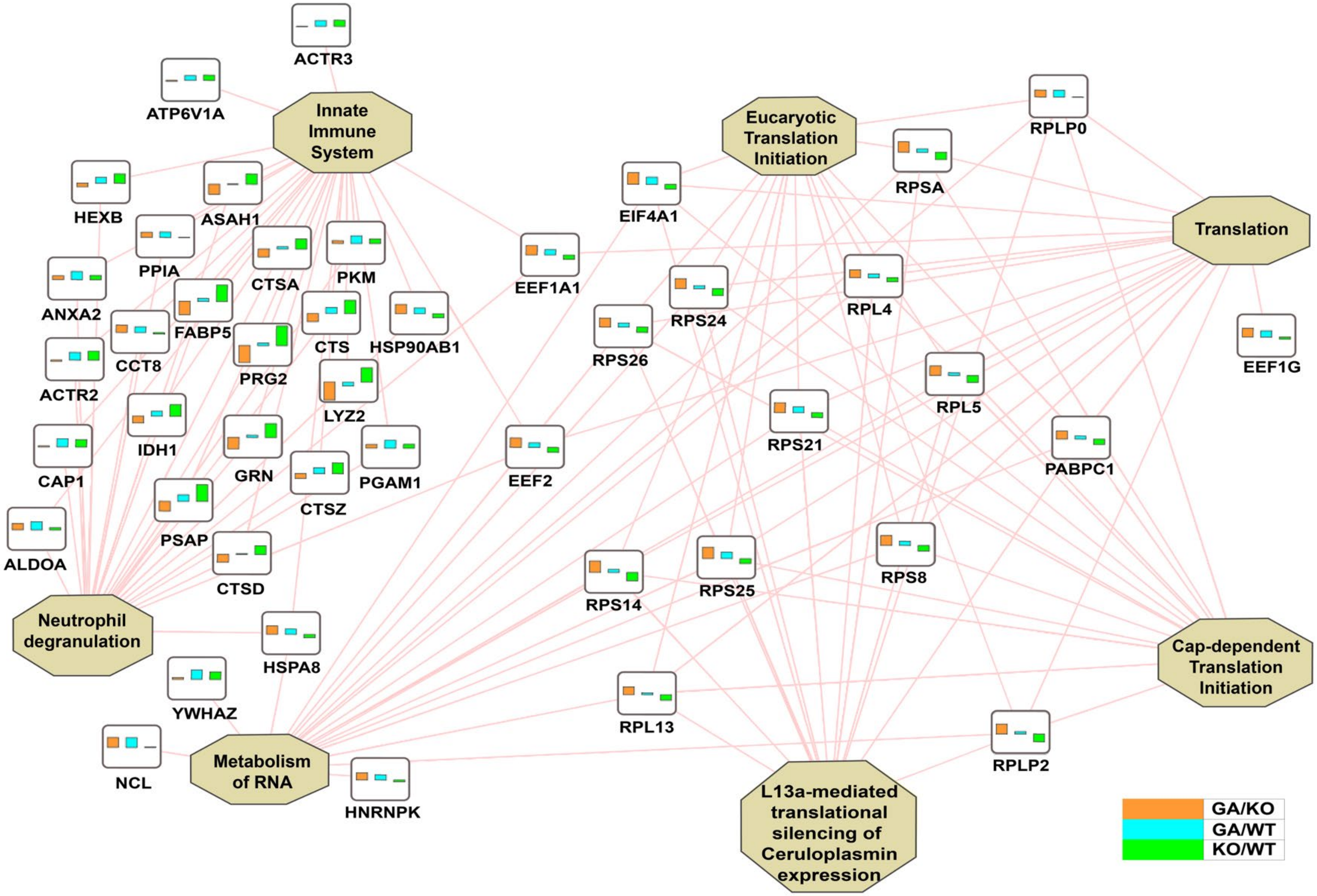

PINE (Protein Interaction Network Extractor) analysis; Top 5 canonical ontology networks were identified using the significant proteins associated with three comparisons. The directions (up/down) of the bars represent the fold change (+/-).

Supplemental Figure S4. Extended data on the effects of OPN deficiency and GA stimulation in BM-derived macrophages.

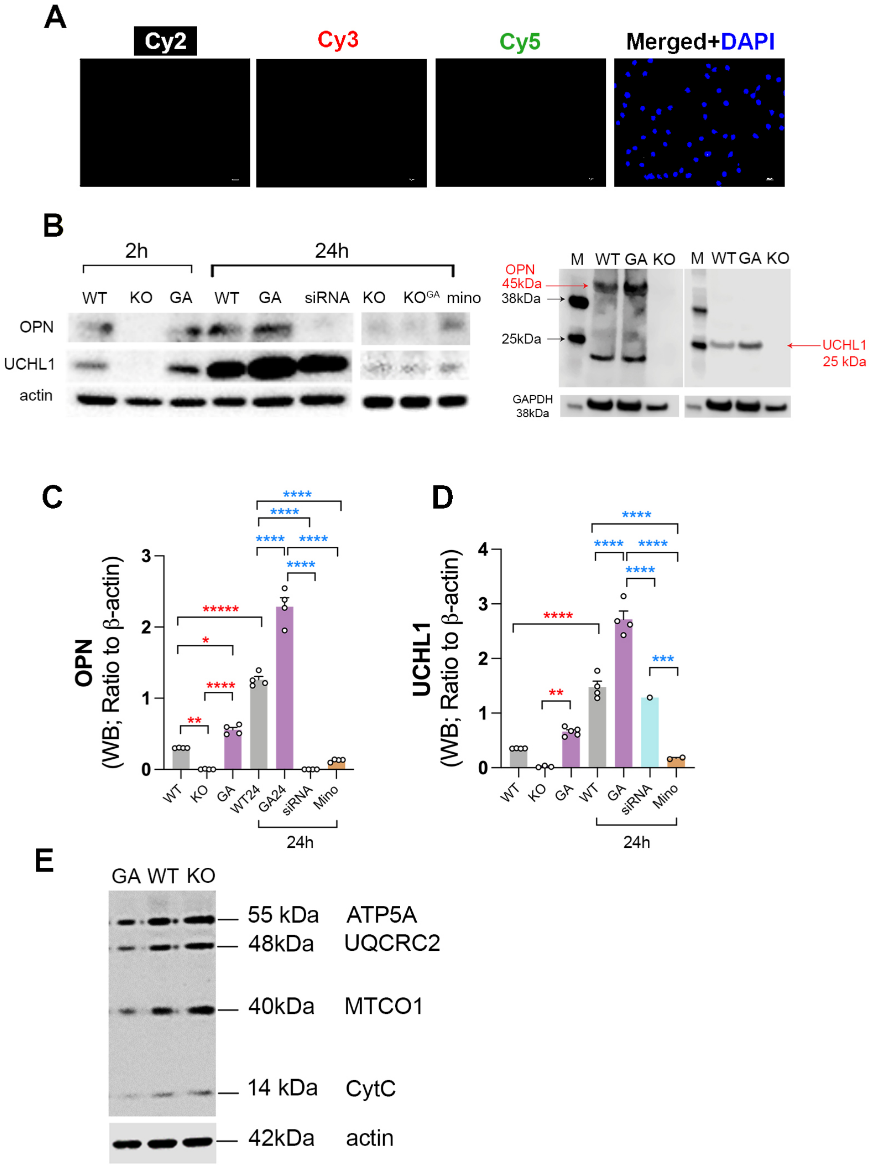

(**A**) Representative fluorescent micrographs of BMMΦ cells with no primary antibodies; scale bar: 20µm. (B) Western blot gels of OPN and UCHL1 bands after 2 and 24 hours of treatment. Quantitative analyses of WB bands for (**C**) OPN and (**D**) UCHL1 normalized to actin. (**E**) WB gel using CytC antibody and OXPHOS antibody cocktail (which contains CI subunit NDUFB8, CII SDHB, CIII-Core protein 2 UQCRC2, CIV subunit I MTCO1 and CV alpha subunit ATP5A). Individual data points, group means, and SEMs are shown. * $p < 0.05$, ** $p < 0.01$, *** $p < 0.001$, **** $p < 0.0001$ by one-way ANOVA with Tukey's post-hoc multiple comparison test. Mino: minocycline; GA: WT+GA; KO: OPN^{KO}; KO^{GA}: OPN^{KO}+GA.

Table S1. List of antibodies used in this study

Primary antibody	host	ICC dilution	WB dilution	Source	catalog#
Annexin V	Rabbit	1:100		Biolegend	671001
ATP-β	Mouse	1:100	1:1000	abcam	ab14748
β-actin	Mouse		1:1000	Santa Cruz biotechnology	sc-47778
CD36	Rat	1:200		abcam	ab80080
CytC	Mouse	1:100	1:1000	Santa Cruz	SC13560
HMOX1	Rabbit	1:500	1:2000	abcam	ab189491
Lamp1	Rat	1:500		abcam	ab25245
MitoTracker^{TM}-Red				Thermofisher	M7512
OPN	Goat	1:100	1:2000	R&D systems	AF808
OXPHOS Cocktail: CI subunit NDUFB8, CII SDHB, CIII-core protein 2 UQCRC2, CIV MTCO1 and CV ATP5A	Mouse		1:250	abcam	ab110413
SOD1	Rabbit	1:100	1:50000	abcam	ab51254
UCHL1 (anti PGP9.5)	Rabbit	1:250	1:5000	abcam	ab108986

**Secondary antibody**				
Cy2	Rabbit, Mouse, Rat, Goat, Sheep	1:200		Jackson ImmunoResearch Laboratories
Cy3		1:200		
Cy5		1:200		
Cy7		1:200		

Table S2. Extended mass spectrometry data on differentially expressed proteins (DEPs) in Bone-marrow derived macrophages (BMMΦ).

Protein	Entry name	Protein names	log2FC (GA/OPN^{KO})	log2FC (GA/WT)	log2FC (OPN^{KO}/WT)
Q9R0P9	UCHL1	Ubiquitin carboxyl-terminal hydrolase isozyme L1 (UCH-L1) (EC 3.4.19.12) (Neuron cytoplasmic protein 9.5) (PGP 9.5) (Ubiquitin thioesterase L1)	1.42001	0.186025	-1.22861
P19123	TNNC1	Troponin C, slow skeletal and cardiac muscles (TN-C)	0.807681	0.0172542	-0.620598
P02535	K1C10	Keratin, type I cytoskeletal 10 (56 kDa cytokeratin) (Cytokeratin-10) (CK-10) (Keratin, type I cytoskeletal 59 kDa) (Keratin-10) (K10)	0.650986	0.818266	0.16728
P62960	YBOX1	Nuclease-sensitive element-binding protein 1 (CCAAT-binding transcription factor I subunit A) (CBF-A) (DNA-binding protein B) (DBPB) (Enhancer factor I subunit A) (EFI-A) (Y-box transcription factor) (YB-1)	0.577589	0.282884	-0.260559
Q920E5	FPPS	Farnesyl pyrophosphate synthase (FPP synthase) (FPS) (EC 2.5.1.10) ((2E,6E)-farnesyl diphosphate synthase) (Cholesterol-regulated 39 kDa protein) (CR 39) (Dimethylallyltranstransferase) (EC 2.5.1.1)	0.539217	0.191668	-0.347548
P08228	SODC	Superoxide dismutase [Cu-Zn] (EC 1.15.1.1)	0.534086	0.276391	-0.21349
P47964	RL36	60S ribosomal protein L36	0.504701	0.0331443	-0.471557
Q8VED5	K2C79	Keratin, type II cytoskeletal 79 (Cytokeratin-79) (CK-79) (Keratin-79) (K79)	0.473037	0.586116	0.113079
P53996	CNBP	Cellular nucleic acid-binding protein (CNBP) (Zinc finger protein 9)	0.471547	0.168283	-0.320566
P27659	RL3	60S ribosomal protein L3 (J1 protein)	0.472965	0.0745936	-0.400684
P62892	RL39	60S ribosomal protein L39	0.448544	0.112429	-0.324516
P14901	HMOX1	Heme oxygenase 1 (HO-1) (EC 1.14.14.18) (P32 protein)	0.446877	-0.0492656	-0.497314
P62274	RS29	40S ribosomal protein S29	0.431545	0.0899444	-0.3416
O09167	RL21	60S ribosomal protein L21	0.427927	-0.0168655	-0.447237
P11688	ITA5	Integrin alpha-5 (CD49 antigen-like family member E) (Fibronectin receptor subunit alpha) (Integrin alpha-F) (VLA-5)	0.412617	0.273738	-0.138879

Q8BU30	SYIC	Isoleucine--tRNA ligase, cytoplasmic (EC 6.1.1.5) (Isoleucyl-tRNA synthetase) (IRS) (IleRS)	0.409182	0.390103	-0.0190789
Q9CZN7	GLYM	Serine hydroxymethyltransferase, mitochondrial (SHMT) (EC 2.1.2.1) (Glycine hydroxymethyltransferase) (Serine methylase)	0.394653	0.230661	-0.163992
Q8BGQ7	SYAC	Alanine--tRNA ligase, cytoplasmic (EC 6.1.1.7) (Alanyl-tRNA synthetase) (AlaRS) (Protein sticky) (Sti)	0.384761	0.200994	-0.184614
P60843	IF4A1	Eukaryotic initiation factor 4A-I (eIF-4A-I) (eIF4A-I) (EC 3.6.4.13) (ATP-dependent RNA helicase eIF4A-1)	0.382044	0.236558	-0.152367
Q8BGD9	IF4B	Eukaryotic translation initiation factor 4B (eIF-4B)	0.377413	0.104519	-0.272894
P70372	ELAV1	ELAV-like protein 1 (Elav-like generic protein) (Hu-antigen R) (HuR)	0.376627	0.292613	-0.0835764
P46471	PRS7	26S proteasome regulatory subunit 7 (26S proteasome AAA-ATPase subunit RPT1) (Proteasome 26S subunit ATPase 2)	0.376073	0.246241	-0.129832
Q8VDM4	PSMD2	26S proteasome non-ATPase regulatory subunit 2 (26S proteasome regulatory subunit RPN1, S2, pp7)	0.370541	0.194439	-0.176102
P62264	RS14	40S ribosomal protein S14	0.37403	0.102531	-0.271818
P62852	RS25	40S ribosomal protein S25	0.368464	0.212205	-0.157823
Q61990	PCBP2	Poly(rC)-binding protein 2 (Alpha-CP2) (CTBP) (CBP) (Putative heterogeneous nuclear ribonucleoprotein X) (hnRNP X)	0.362983	0.165224	-0.197759
Q9DCL9	PUR6	Multifunctional protein ADE2 [Includes: Phosphoribosylaminoimidazole-succinocarboxamide synthase (EC 6.3.2.6) (SAICAR synthetase); (AIRC)]	0.36039	-0.0153663	-0.375756
P63028	TCTP	Translationally-controlled tumor protein (TCTP) (21 kDa polypeptide) (p21)	0.352695	0.222377	-0.131568
P19253	RL13A	60S ribosomal protein L13a (Transplantation antigen P198) (Tum-P198 antigen)	0.352332	0.122744	-0.229588
P80316	TCPE	T-complex protein 1 subunit epsilon (TCP-1-epsilon) (CCT-epsilon)	0.349995	0.248173	-0.101822
Q91V12	BACH	Cytosolic acyl coenzyme A thioester hydrolase (EC 3.1.2.2) (Acyl-CoA thioesterase 7) (Brain acyl-CoA hydrolase) (BACH) (CTE-IIa)	0.347064	0.125118	-0.221946

P61358	RL27	60S ribosomal protein L27	0.347031	0.158398	-0.164689
P62267	RS23	40S ribosomal protein S23	0.345916	0.128962	-0.216953
P14733	LMNB1	Lamin-B1	0.345665	0.394409	0.0487443
P14206	RSSA	40S ribosomal protein SA (37 kDa laminin receptor precursor) (37LRP) (37 kDa oncofetal antigen) (37/67 kDa laminin receptor) (LRP/LR) (67 kDa laminin receptor) (67LR) (Laminin receptor 1) (LamR) (LBP/p40)	0.344913	0.106974	-0.236206
Q9EQU5	SET	Protein SET (Phosphatase 2A inhibitor I2PP2A) (I-2PP2A) (Template-activating factor I) (TAF-I)	0.340867	0.32709	-0.0137766
P62862	RS30	40S ribosomal protein S30	0.338078	0.166602	-0.144709
P01942	HBA	Hemoglobin subunit alpha (Alpha-globin) (Hemoglobin alpha chain)	0.337718	-0.21075	-0.548467
P17918	PCNA	Proliferating cell nuclear antigen (PCNA) (Cyclin)	0.336729	0.205661	-0.129509
Q8CGC7	SYEP	Bifunctional glutamate/proline--tRNA ligase (Bifunctional aminoacyl-tRNA synthetase) Glutamate--tRNA ligase (Glutamyl-tRNA synthetase) (GluRS);	0.336242	0.390795	0.0545534
Q6ZWV7	RL35	60S ribosomal protein L35	0.336122	-0.0644733	-0.400849
P47955	RLA1	60S acidic ribosomal protein P1	0.33336	0.188513	-0.144847
Q9CQR2	RS21	40S ribosomal protein S21	0.332877	0.185106	-0.148191
P50580	PA2G4	Proliferation-associated protein 2G4 (IRES-specific cellular trans-acting factor 45 kDa) (ITAF45) (Mpp1) (Protein p38-2G4)	0.331339	0.218689	-0.113736
Q922F4	TBB6	Tubulin beta-6 chain	0.331212	0.237895	-0.0933172
P99027	RLA2	60S acidic ribosomal protein P2	0.330343	0.0841261	-0.246217
Q61490	CD166	CD166 antigen (Activated leukocyte cell adhesion molecule) (BEN) (Protein DM-GRASP) (CD antigen CD166)	0.329348	0.103768	-0.219518
P62849	RS24	40S ribosomal protein S24	0.324828	0.0982284	-0.216558
Q9R1Q7	PLP2	Proteolipid protein 2	0.323528	0.252591	-0.0714836
P63323	RS12	40S ribosomal protein S12	0.321915	0.162393	-0.16281
O08807	PRDX4	Peroxiredoxin-4 (EC 1.11.1.15) (Antioxidant enzyme AOE372) (Peroxiredoxin IV) (Prx-IV) (Thioredoxin peroxidase AO372)	0.319703	-0.0275142	-0.347217

Q99KP6	PRP19	Pre-mRNA-processing factor 19 (EC 2.3.2.27) (Nuclear matrix protein 200) (PRP19/PSO4 homolog) (RING-type E3 ubiquitin transferase PRP19) (Senescence evasion factor)	0.318976	0.222919	-0.0960577
P62242	RS8	40S ribosomal protein S8	0.317116	0.137992	-0.177802
P09405	NUCL	Nucleolin (Protein C23)	0.31566	0.315715	0.0170102
P47962	RL5	60S ribosomal protein L5	0.314622	0.0907441	-0.224337
P32020	NLTP	Non-specific lipid-transfer protein (NSL-TP) (EC 2.3.1.176) (Propanoyl-CoA C-acyltransferase) (SCP-chi) (SCPX) (Sterol carrier protein 2) (SCP-2)	0.314107	0.295221	-0.0188857
P80317	TCPZ	T-complex protein 1 subunit zeta (TCP-1-zeta) (CCT-zeta-1)	0.314035	0.30634	-0.00866062
Q8R1B4	EIF3C	Eukaryotic translation initiation factor 3 subunit C (eIF3c) (Eukaryotic translation initiation factor 3 subunit 8) (eIF3 p110)	0.310941	0.264668	-0.0471196
Q61656	DDX5	Probable ATP-dependent RNA helicase DDX5 (EC 3.6.4.13) (DEAD box RNA helicase DEAD1) (mDEAD1) (DEAD box protein 5) (RNA helicase p68)	0.308833	0.151501	-0.191618
P58252	EF2	Elongation factor 2 (EF-2)	0.308598	0.146926	-0.162949
P10126	EF1A1	Elongation factor 1-alpha 1 (EF-1-alpha-1) (Elongation factor Tu) (EF-Tu) (Eukaryotic elongation factor 1 A-1) (eEF1A-1)	0.307464	0.165162	-0.142257
P62843	RS15	40S ribosomal protein S15 (RIG protein)	0.307053	0.244635	-0.062418
P11499	HS90B	Heat shock protein HSP 90-beta (Heat shock 84 kDa) (HSP 84) (HSP84) (Tumor-specific transplantation 84 kDa antigen) (TSTA)	0.304861	0.178227	-0.125736
P62827	RAN	GTP-binding nuclear protein Ran (GTPase Ran) (Ras-like protein TC4)	0.303851	0.2213	-0.0784094
P25444	RS2	40S ribosomal protein S2 (40S ribosomal S4) (Protein LLRep3)	0.30666	0.132817	-0.181735
P62855	RS26	40S ribosomal protein S26	0.301968	0.136632	-0.1664
Q9QXS1	PLEC	Plectin (PCN) (PLTN) (Plectin-1) (Plectin-6)	0.300082	0.195975	-0.102634

P70168	IMB1	Importin subunit beta-1 (Karyopherin subunit beta-1) (Nuclear factor p97) (Pore targeting complex 97 kDa subunit) (PTAC97) (SCG)	0.296331	0.302245	0.00608378
P63276	RS17	40S ribosomal protein S17	0.296286	0.191906	-0.104838
P62751	RL23A	60S ribosomal protein L23a	0.295175	0.231599	-0.0634288
P97351	RS3A	40S ribosomal protein S3a (Protein TU-11)	0.287884	0.137803	-0.15494
P48787	TNNI3	Troponin I, cardiac muscle (Cardiac troponin I)	0.285472	-1.4562	-1.47703
Q8BWY3	ERF1	Eukaryotic peptide chain release factor subunit 1 (eRF1)	0.283599	0.182274	-0.101325
P62918	RL8	60S ribosomal protein L8	0.283242	0.0372385	-0.245792
Q9CWJ9	PUR9	Bifunctional purine biosynthesis protein PURH [Includes: Phosphoribosylaminoimidazolecarboxamide formyltransferase (EC 2.1.2.3) (5-aminoimidazole-4-carboxamide ribonucleotide formyltransferase) (AICAR transformylase); IMP cyclohydrolase (ATIC) (IMP synthase) (Inosinicase)]	0.281915	0.183297	-0.0980504
P62281	RS11	40S ribosomal protein S11	0.280612	0.100115	-0.175673
P62754	RS6	40S ribosomal protein S6 (Phosphoprotein NP33)	0.278146	0.0427578	-0.243945
P62334	PRS10	26S proteasome regulatory subunit 10B (26S proteasome AAA-ATPase subunit RPT4) (Proteasome 26S subunit ATPase 6, p42)	0.27773	0.216721	-0.0692083
Q9D8N0	EF1G	Elongation factor 1-gamma (EF-1-gamma) (eEF-1B gamma)	0.277147	0.20281	-0.0737564
P99026	PSB4	Proteasome subunit beta type-4 (EC 3.4.25.1) (Low molecular mass protein 3) (Macropain beta chain) (Proteasome chain 3)	0.277013	0.104985	-0.172027
P52927	HMGA2	High mobility group protein HMGI-C (High mobility group AT-hook protein 2)	0.275216	0.219431	-0.0592098
P62900	RL31	60S ribosomal protein L31	0.274957	0.101708	-0.172018
P12970	RL7A	60S ribosomal protein L7a (Surfeit locus protein 3)	0.27431	-0.0149997	-0.291177
P63017	HSP7C	Heat shock cognate 71 kDa protein (Heat shock 70 kDa protein 8)	0.27346	0.166669	-0.107873
P56959	FUS	RNA-binding protein FUS (Protein pigpen)	0.272407	0.287665	0.0157473
Q9CZX8	RS19	40S ribosomal protein S19	0.269657	0.059528	-0.197957

P29595	NEDD8	NEDD8 (Neddylin) (Neural precursor cell expressed developmentally down-regulated protein 8) (NEDD-8) (Ubiquitin-like protein Nedd8)	0.266018	0.0443138	-0.221704
Q60692	PSB6	Proteasome subunit beta type-6 (EC 3.4.25.1) (Low molecular mass protein 19) (Proteasome delta chain) (Proteasome subunit Y)	0.265718	0.125223	-0.128101
Q8CI51	PDLI5	PDZ and LIM domain protein 5 (Enigma homolog) (Enigma-like PDZ and LIM domains protein)	0.264481	0.196712	-0.0677691
Q9CZD3	GARS	Glycine--tRNA ligase (EC 3.6.1.17) (Diadenosine tetraphosphate synthetase) (AP-4-A synthetase) (Glycyl-tRNA synthetase) (GlyRS)	0.263707	0.203626	-0.060081
P68040	RACK1	Receptor of activated protein C kinase 1 (12-3) (Guanine nucleotide-binding protein subunit beta-2-like 1) (Receptor for activated C kinase) (Receptor of activated protein kinase C 1) (p205)	0.26369	0.200976	-0.061855
P29341	PABP1	Polyadenylate-binding protein 1 (PABP-1) (Poly(A)-binding protein 1)	0.263432	0.0969476	-0.165568
P21981	TGM2	Protein-glutamine gamma-glutamyltransferase 2 (EC 2.3.2.13) (Tissue transglutaminase) (Transglutaminase C) (TG(C)) (TGC)	0.262447	0.165081	-0.0738558
P60335	PCBP1	Poly(rC)-binding protein 1 (Alpha-CP1) (Heterogeneous nuclear ribonucleoprotein E1) (hnRNP E1)	0.261396	0.19003	-0.070748
P70349	HINT1	Histidine triad nucleotide-binding protein 1 (EC 3) (Adenosine 5'-monophosphoramidase) (Protein kinase C inhibitor 1) (PKCI-1)	0.261254	0.25689	-0.00436412
P57776	EF1D	Elongation factor 1-delta (EF-1-delta)	0.261002	0.179539	-0.0913114
P62301	RS13	40S ribosomal protein S13	0.260673	0.130282	-0.138543
P60867	RS20	40S ribosomal protein S20	0.259576	0.194892	-0.0640193
P19973	LSP1	Lymphocyte-specific protein 1 (52 kDa phosphoprotein) (pp52) (Lymphocyte-specific antigen WP34) (S37 protein)	0.258828	0.278622	0.020821
P48428	TBCA	Tubulin-specific chaperone A (TCP1-chaperonin cofactor A) (Tubulin-folding cofactor A) (CFA)	0.258228	0.152816	-0.105412

Q9QXT0	CNPY2	Protein canopy homolog 2 (MIR-interacting saposin-like protein) (Putative secreted protein ZSIG9) (Transmembrane protein 4)	0.25737	0.27873	0.0213607
Q9EQK5	MVP	Major vault protein (MVP)	0.255414	0.237701	-0.0170916
Q7TPV4	MBB1A	Myb-binding protein 1A (Myb-binding protein of 160 kDa)	0.254547	0.17388	0.010707
Q62186	SSRD	Translocon-associated protein subunit delta (TRAP-delta) (Signal sequence receptor subunit delta) (SSR-delta)	0.253738	0.305938	0.0522002
P48678	LMNA	Prelamin-A/C [Cleaved into: Lamin-A/C]	0.253112	0.259696	0.00892314
O55222	ILK	Integrin-linked protein kinase (EC 2.7.11.1)	0.250942	0.195069	-0.055873
P47911	RL6	60S ribosomal protein L6 (TAX-responsive enhancer element-binding protein 107) (TAXREB107)	0.250237	0.0272965	-0.219454
P19096	FAS	Fatty acid synthase (EC 2.3.1.85) [Includes: [Acyl-carrier-protein] S-acetyltransferase (EC 2.3.1.38); [Acyl-carrier-protein] S-malonyltransferase	0.249038	-0.0610557	-0.312759
P35980	RL18	60S ribosomal protein L18	0.24868	0.131846	-0.118469
Q9D0I9	SYRC	Arginine--tRNA ligase, cytoplasmic (EC 6.1.1.19) (Arginyl-tRNA synthetase) (ArgRS)	0.248388	0.377668	0.129281
Q9D8E6	RL4	60S ribosomal protein L4	0.248177	0.115987	-0.1336
O35226	PSMD4	26S proteasome non-ATPase regulatory subunit 4 (26S proteasome regulatory subunit RPN10) (26S proteasome regulatory subunit S5A)	0.247842	0.223437	-0.0245184
P47963	RL13	60S ribosomal protein L13 (A52)	0.246755	0.0638071	-0.178308
P62082	RS7	40S ribosomal protein S7	0.246062	0.014085	-0.232872
P15532	NDKA	Nucleoside diphosphate kinase A (NDK A) (NDP kinase A) (EC 2.7.4.6) (Metastasis inhibition factor NM23) (NDPK-A)	0.24606	0.1423	-0.103132
Q69ZN7	MYOF	Myoferlin (Fer-1-like protein 3)	0.245965	0.445157	0.199191
P80315	TCPD	T-complex protein 1 subunit delta (TCP-1-delta) (A45) (CCT-delta)	0.245913	0.231593	-0.0133915
Q9QYB1	CLIC4	Chloride intracellular channel protein 4 (mc3s5/mtCLIC)	0.244906	0.180616	-0.064746

P07091	S10A4	Protein S100-A4 (Metastasin) (Metastatic cell protein) (PEL98) (Placental calcium-binding protein) (Protein 18A2) (S100 calcium-binding protein A4)	0.244352	0.132844	-0.12257
P62702	RS4X	40S ribosomal protein S4, X isoform	0.243703	0.114525	-0.132788
Q9DB05	SNAA	Alpha-soluble NSF attachment protein (SNAP-alpha) (N-ethylmaleimide-sensitive factor attachment protein alpha)	0.242203	0.187143	-0.0560178
P62270	RS18	40S ribosomal protein S18 (Ke-3) (Ke3)	0.24156	0.155996	-0.0847027
Q91VI7	RINI	Ribonuclease inhibitor (Ribonuclease/angiogenin inhibitor 1)	0.240166	0.286041	0.0451989
P17095	HMGA1	High mobility group protein HMG-I/HMG-Y (HMG-I(Y)) (High mobility group AT-hook protein 1) (High mobility group protein A1)	0.239711	0.223706	-0.0160048
O70251	EF1B	Elongation factor 1-beta (EF-1-beta)	0.239264	0.192464	-0.0476734
P09411	PGK1	Phosphoglycerate kinase 1 (EC 2.7.2.3)	0.239156	0.247229	0.00846945
P63325	RS10	40S ribosomal protein S10	0.239591	0.237064	-0.00927722
Q9CXW4	RL11	60S ribosomal protein L11	0.239128	0.224268	-0.0148523
O08795	GLU2B	Glucosidase 2 subunit beta (80K-H protein) (Glucosidase II subunit beta) (Protein kinase C substrate 60.1 kDa protein heavy chain)	0.239067	0.228847	-0.0102198
Q8BP67	RL24	60S ribosomal protein L24	0.238593	-0.0357115	-0.275312
P28656	NP1L1	Nucleosome assembly protein 1-like 1 (Brain protein DN38) (NAP-1-related protein)	0.237429	0.27301	0.03802
P42932	TCPQ	T-complex protein 1 subunit theta (TCP-1-theta) (CCT-theta)	0.237377	0.185334	-0.0525802
P62983	RS27A	Ubiquitin-40S ribosomal protein S27a (Ubiquitin carboxyl extension protein 80) [Cleaved into: Ubiquitin; 40S ribosomal protein S27a]	0.237266	0.251401	0.0141356
P62908	RS3	40S ribosomal protein S3 (EC 4.2.99.18)	0.23573	0.125538	-0.113537
Q01768	NDKB	Nucleoside diphosphate kinase B (NDK B) (NDP kinase B) (EC 2.7.4.6) (Histidine protein kinase NDKB) (EC 2.7.13.3) (P18) (nm23-M2)	0.234491	0.27962	0.0357358

P26883	FKB1A	Peptidyl-prolyl cis-trans isomerase FKBP1A (PPIase FKBP1A) (EC 5.2.1.8) (12 kDa FK506-binding protein) (12 kDa FKBP) (FKBP-12) (Calstabin-1)	0.233356	0.216678	-0.0169412
Q6ZWV3	RL10	60S ribosomal protein L10 (Protein QM homolog) (Ribosomal protein L10)	0.233284	0.192506	-0.0407782
P14869	RLA0	60S acidic ribosomal protein P0 (60S ribosomal protein L10E)	0.232858	0.215545	-0.00712
Q61263	SOAT1	Sterol O-acyltransferase 1 (EC 2.3.1.26) (Acyl-coenzyme A:cholesterol acyltransferase 1) (ACAT-1)	0.232626	-0.0168912	-0.249517
Q61316	HSP74	Heat shock 70 kDa protein 4 (Heat shock 70-related protein APG-2)	0.232552	0.0719468	-0.159949
Q9WV80	SNX1	Sorting nexin-1	0.232373	0.1957	-0.0371826
P26638	SYSC	Serine--tRNA ligase, cytoplasmic (EC 6.1.1.11) (Seryl-tRNA synthetase) (SerRS) (Seryl-tRNA(Ser/Sec) synthetase)	0.230753	0.365658	0.134905
P61979	HNRPK	Heterogeneous nuclear ribonucleoprotein K (hnRNP K)	0.230537	0.173722	-0.0571164
P31938	MP2K1	Dual specificity mitogen-activated protein kinase kinase 1 (MAP kinase kinase 1) (MAPKK 1) (ERK activator kinase 1) (MAPK/ERK kinase 1)	0.230143	0.236897	0.00675399
Q8BTM8	FLNA	Filamin-A (FLN-A) (Actin-binding protein 280) (ABP-280) (Alpha-filamin) (Endothelial actin-binding protein) (Filamin-1)	0.229313	0.189585	-0.0420707
P80314	TCPB	T-complex protein 1 subunit beta (TCP-1-beta) (CCT-beta)	0.228241	0.172366	-0.0508776
P80318	TCPG	T-complex protein 1 subunit gamma (TCP-1-gamma) (CCT-gamma) (Matricin) (mTRiC-P5)	0.228037	0.155261	-0.0716604
Q62159	RHOC	Rho-related GTP-binding protein RhoC (Silica-induced gene 61 protein) (SIG-61)	0.227991	0.127264	-0.100727
P06151	LDHA	L-lactate dehydrogenase A chain (LDH-A) (EC 1.1.1.27) (LDH muscle subunit) (LDH-M)	0.226983	0.273933	0.0548976
P09055	ITB1	Integrin beta-1 (Fibronectin receptor subunit beta) (VLA-4 subunit beta) (CD antigen CD29)	0.226806	0.0882738	-0.138532
P40142	TKT	Transketolase (TK) (EC 2.2.1.1) (P68)	0.225699	0.20532	-0.0168953
Q9WVK4	EHD1	EH domain-containing protein 1 (PAST homolog 1) (mPAST1)	0.224366	0.180218	-0.0442826

Q9JHU4	DYHC1	Cytoplasmic dynein 1 heavy chain 1 (Cytoplasmic dynein heavy chain 1) (Dynein heavy chain, cytosolic)	0.224268	0.275861	0.0515928
P11983	TCPA	T-complex protein 1 subunit alpha (TCP-1-alpha) (CCT-alpha) (Tailless complex polypeptide 1A) (TCP-1-A) (Tailless complex polypeptide 1B) (TCP-1-B)	0.22239	0.170819	-0.0513757
Q99JI4	PSMD6	26S proteasome non-ATPase regulatory subunit 6 (26S proteasome regulatory subunit RPN7) (26S proteasome regulatory suS10) (p42A)	0.223441	0.125315	-0.0981259
Q91VW3	SH3L3	SH3 domain-binding glutamic acid-rich-like protein 3	0.222755	0.121284	-0.101471
Q9Z1Z2	STRAP	Serine-threonine kinase receptor-associated protein (UNR-interacting protein)	0.221815	0.195557	-0.0262579
P99024	TBB5	Tubulin beta-5 chain	0.221666	0.202301	-0.0206526
O70310	NMT1	Glycylpeptide N-tetradecanoyltransferase 1 (EC 2.3.1.97) (Myristoyl-CoA:protein N-myristoyltransferase 1) (NMT 1)	0.221176	0.103285	-0.117892
P15379	CD44	CD44 antigen (Extracellular matrix receptor III) (ECMR-III) (GP90 lymphocyte homing/adhesion receptor) (HUTCH-I) (Lymphocyte antigen 24) (Ly-24) (Phagocytic glycoprotein 1) (PGP-1)	0.220535	0.181472	-0.0421339
Q9R1P4	PSA1	Proteasome subunit alpha type-1 (EC 3.4.25.1) (Multicatalytic endopeptidase complex subunit C2) (Proteasome component C2)	0.219098	0.259071	0.0400035
P67984	RL22	60S ribosomal protein L22 (Heparin-binding protein HBp15)	0.218597	0.148375	-0.0733444
Q02053	UBA1	Ubiquitin-like modifier-activating enzyme 1 (EC 6.2.1.45) (Ubiquitin-activating enzyme E1) (Ubiquitin-activating enzyme E1 X)	0.214458	0.241132	0.0258766
Q9WVA4	TAGL2	Transgelin-2 (SM22-beta)	0.216108	0.22121	0.00730317
P14115	RL27A	60S ribosomal protein L27a (L29)	0.215413	0.156173	-0.0592404
P68372	TBB4B	Tubulin beta-4B chain (Tubulin beta-2C chain)	0.214653	0.225	0.0103477
P63254	CRIP1	Cysteine-rich protein 1 (CRP-1) (Cysteine-rich intestinal protein) (CRIP)	0.211637	0.0927406	-0.118896
Q9R0P3	ESTD	S-formylglutathione hydrolase (FGH) (EC 3.1.2.12) (Esterase 10) (Esterase D) (Sid 478)	0.210909	0.242927	0.0327457

P24668	MPRD	Cation-dependent mannose-6-phosphate receptor (CD Man-6-P receptor) (CD-MPR) (46 kDa mannose 6-phosphate receptor) (MPR 46)	0.210275	0.187214	-0.023063
P62315	SMD1	Small nuclear ribonucleoprotein Sm D1 (Sm-D1) (Sm-D autoantigen) (snRNP core protein D1)	0.210256	0.086072	-0.124184
P05064	ALDOA	Fructose-bisphosphate aldolase A (EC 4.1.2.13) (Aldolase 1) (Muscle-type aldolase)	0.209785	0.274356	0.0656065
P10107	ANXA1	Annexin A1 (Annexin I) (Annexin-1) (Calpactin II) (Calpactin-2) (Chromobindin-9) (Lipocortin I) (Phospholipase A2 inhibitory protein) (p35)	0.207357	0.208832	0.00137192
P35979	RL12	60S ribosomal protein L12	0.205539	0.0770894	-0.126412
P97461	RS5	40S ribosomal protein S5 [Cleaved into: 40S ribosomal protein S5, N-terminally processed]	0.205093	-0.0732576	-0.238361
P41105	RL28	60S ribosomal protein L28	0.204846	-0.0236536	-0.2285
P07901	HS90A	Heat shock protein HSP 90-alpha (Heat shock 86 kDa) (HSP 86) (HSP86) (Tumor-specific transplantation 86 kDa antigen) (TSTA)	0.204413	0.304361	0.0989716
Q6ZWX6	IF2A	Eukaryotic translation initiation factor 2 subunit 1 (Eukaryotic translation initiation factor 2 subunit alpha) (eIF-2-alpha) (eIF-2A) (eIF-2alpha)	0.202728	0.171078	-0.0316677
P17182	ENOA	Alpha-enolase (EC 4.2.1.11) (2-phospho-D-glycerate hydro-lyase) (Enolase 1) (Non-neural enolase) (NNE)	0.202042	0.236642	0.0336817
Q9QUH0	GLRX1	Glutaredoxin-1 (Thioltransferase-1) (TTase-1)	0.200815	0.297731	0.0993147
Q99N69	LPXN	Leupaxin	0.199855	0.171326	-0.0285292
Q76MZ3	2AAA	Serine/threonine-protein phosphatase 2A 65 kDa regulatory subunit A alpha isoform (PP2A subunit A isoform PR65-alpha) (PP2A subunit A isoform R1-alpha)	0.198352	0.407793	0.21266
Q60870	REEP5	Receptor expression-enhancing protein 5 (GP106) (Polyposis locus protein 1 homolog) (Protein TB2 homolog)	0.197493	0.0867302	-0.110763
O08529	CAN2	Calpain-2 catalytic subunit (EC 3.4.22.53) (80 kDa M-calpain subunit) (CALP80) (Calcium-activated neutral proteinase 2) (CANP 2)	0.197272	0.167238	-0.0372826

P26039	TLN1	Talin-1	0.197211	0.169232	-0.0273912
P34022	RANG	Ran-specific GTPase-activating protein (HpaII tiny fragments locus 9a protein) (Ran-binding protein 1) (RANBP1)	0.196572	0.565265	0.368693
O70318	E41L2	Band 4.1-like protein 2 (Generally expressed protein 4.1) (4.1G)	0.196187	0.357881	0.161694
Q8VDN2	AT1A1	Sodium/potassium-transporting ATPase subunit alpha-1 (Na(+)/K(+) ATPase alpha-1 subunit) (EC 3.6.3.9) (Sodium pump subunit alpha-1)	0.194784	-0.169168	-0.363952
Q9R0Q7	TEBP	Prostaglandin E synthase 3 (EC 5.3.99.3) (Cytosolic prostaglandin E2 synthase) (cPGES) (Hsp90 co-chaperone) (Progesterone receptor complex p23) (Sid 3177) (Telomerase-binding protein p23)	0.193643	0.382457	0.188917
P68510	1433F	14-3-3 protein eta	0.193481	0.298155	0.117206
P16110	LEG3	Galectin-3 (Gal-3) (35 kDa lectin) (Carbohydrate-binding protein 35) (CBP 35) (Galactose-specific lectin 3) (IgE-binding protein) (L-34 galactoside-binding lectin) (Laminin-binding protein) (Lectin L-29) (Mac-2 antigen)	0.193035	0.211854	0.0188189
Q8VIJ6	SFPQ	Splicing factor, proline- and glutamine-rich (DNA-binding p52/p100 complex, 100 kDa subunit) (Polypyrimidine tract-binding protein-associated-splicing factor) (PSF) (PTB-associated-splicing factor)	0.191873	0.238764	0.0468906
P84099	RL19	60S ribosomal protein L19	0.190803	0.109354	-0.0815168
P45878	FKBP2	Peptidyl-prolyl cis-trans isomerase FKBP2 (EC 5.2.1.8) (13 kDa FK506-binding protein) (13 kDa FKBP) (FKBP-2) (Immunophilin FKBP13) (Rotamase)	0.191913	0.117859	-0.0740536
P14131	RS16	40S ribosomal protein S16	0.189745	0.0679746	-0.118532
Q9DBS1	TMM43	Transmembrane protein 43 (Protein LUMA)	0.189611	0.125901	-0.0563432
P17751	TPIS	Triosephosphate isomerase (TIM) (EC 5.3.1.1) (Triose-phosphate isomerase)	0.189184	0.218713	0.0295656
Q61937	NPM	Nucleophosmin (NPM) (Nucleolar phosphoprotein B23) (Nucleolar protein NO38) (Numatrin)	0.188251	0.170916	-0.0165788
Q922B2	SYDC	Aspartate--tRNA ligase, cytoplasmic (EC 6.1.1.12) (Aspartyl-tRNA synthetase) (AspRS)	0.186047	0.123629	-0.0624786

E9Q634	MYO1E	Unconventional myosin-Ie (Unconventional myosin 1E)	0.185552	0.126366	-0.0591313
P62196	PRS8	26S proteasome regulatory subunit 8 (26S proteasome AAA-ATPase subunit RPT6) (Proteasome 26S subunit ATPase 5) (Proteasome subunit p45) (p45/SUG) (mSUG1)	0.18516	0.233293	0.0481322
P55302	AMRP	Alpha-2-macroglobulin receptor-associated protein (Alpha-2-MRAP) (Heparin-binding protein 44) (HBP-44) (RAP)	0.185071	0.0595902	-0.125481
Q3U0V1	FUBP2	Far upstream element-binding protein 2 (FUSE-binding protein 2) (KH type-splicing regulatory protein) (KSRP)	0.184448	0.0869048	-0.0981977
Q8VDD5	MYH9	Myosin-9 (Cellular myosin heavy chain, type A) (Myosin heavy chain 9) (Myosin heavy chain, non-muscle IIa)	0.181065	0.153212	-0.0209035
P08113	ENPL	Endoplasmin (94 kDa glucose-regulated protein) (GRP-94) (Endoplasmic reticulum resident protein 99) (ERp99) (Heat shock protein 90 kDa beta member 1) (Tumor rejection antigen gp96)	0.180514	0.108291	-0.0717133
Q9DBC7	KAP0	cAMP-dependent protein kinase type I-alpha regulatory subunit	0.180041	-0.0056845	-0.185725
P11835	ITB2	Integrin beta-2 (Cell surface adhesion glycoproteins LFA-1/CR3/p150,95 subunit beta) (Complement receptor C3 subunit beta) (CD antigen CD18)	0.181889	0.155537	-0.0252691
P08003	PDIA4	Protein disulfide-isomerase A4 (EC 5.3.4.1) (Endoplasmic reticulum resident protein 72) (ER protein 72) (ERp-72) (ERp72)	0.179249	0.236447	0.0181138
Q9QUM9	PSA6	Proteasome subunit alpha type-6 (EC 3.4.25.1) (Macropain iota chain) (Multicatalytic endopeptidase complex iota chain) (Proteasome iota chain)	0.176824	0.130873	-0.0470054
Q9EST5	AN32B	Acidic leucine-rich nuclear phosphoprotein 32 family member B (Proliferation-related acidic leucine-rich protein PAL31)	0.176606	0.209859	0.033253
Q9Z1Q5	CLIC1	Chloride intracellular channel protein 1 (Nuclear chloride ion channel 27) (NCC27)	0.17557	0.215178	0.0424806
P16858	G3P	Glyceraldehyde-3-phosphate dehydrogenase (GAPDH) (EC 1.2.1.12) (Peptidyl-cysteine S-nitrosylase GAPDH)	0.1754	0.192814	0.0158725

O70145	NCF2	Neutrophil cytosol factor 2 (NCF-2) (67 kDa neutrophil oxidase factor) (Neutrophil NADPH oxidase factor 2) (p67-phox)	0.174235	0.206844	0.0326091
P17742	PPIA	Peptidyl-prolyl cis-trans isomerase A (PPIase A) (EC 5.2.1.8) (Cyclophilin A) (Cyclosporin A-binding protein) (Rotamase A) (SP18)	0.17338	0.18103	0.00902836
P62889	RL30	60S ribosomal protein L30	0.173735	0.055003	-0.118685
Q9JKR6	HYOU1	Hypoxia up-regulated protein 1 (GRP-170) (140 kDa Ca(2+)-binding protein) (CBP-140)	0.173699	0.254932	0.0812336
P84104	SRSF3	Serine/arginine-rich splicing factor 3 (Pre-mRNA-splicing factor SRP20) (Protein X16) (Splicing factor, arginine/serine-rich 3)	0.17211	0.210364	0.0382538
P14148	RL7	60S ribosomal protein L7	0.170621	-0.080258	-0.249397
Q62426	CYTB	Cystatin-B (Stefin-B)	0.168527	0.250467	0.0785036
P49710	HCLS1	Hematopoietic lineage cell-specific protein (Hematopoietic cell-specific LYN substrate 1) (LckBP1)	0.167798	0.18018	0.0133557
Q9CZM2	RL15	60S ribosomal protein L15	0.16696	0.101949	-0.0652005
P28063	PSB8	Proteasome subunit beta type-8 (EC 3.4.25.1) (Low molecular mass protein 7) (Proteasome component C13) (Proteasome subunit beta-5i)	0.166736	0.266088	0.0993519
P57759	ERP29	Endoplasmic reticulum resident protein 29 (ERp29)	0.166425	0.161614	0.00248708
Q9R1P1	PSB3	Proteasome subunit beta type-3 (EC 3.4.25.1) (Proteasome chain 13) (Proteasome component C10-II) (Proteasome theta chain)	0.165858	0.324746	0.158939
Q6WVG3	KCD12	BTB/POZ domain-containing protein KCTD12 (Pfetin) (Predominantly fetal expressed T1 domain)	0.164935	0.229557	0.0795686
Q9D358	PPAC	Low molecular weight phosphotyrosine protein phosphatase (LMW-PTP) (LMW-PTPase) (EC 3.1.3.48) (Low molecular weight cytosolic acid phosphatase) (EC 3.1.3.2)	0.164478	0.12533	-0.0391477
P61982	1433G	14-3-3 protein gamma [Cleaved into: 14-3-3 protein gamma, N-terminally processed]	0.163942	0.273477	0.105555
Q9CZ30	OLA1	Obg-like ATPase 1 (GTP-binding protein 9)	0.161804	0.0970077	-0.0648

P26350	PTMA	Prothymosin alpha [Cleaved into: Prothymosin alpha, N-terminally processed; Thymosin alpha]	0.161176	0.228868	0.0676925
Q61792	LASP1	LIM and SH3 domain protein 1 (LASP-1) (Metastatic lymph node gene 50 protein) (MLN 50)	0.160253	0.193527	0.0348269
P17427	AP2A2	AP-2 complex subunit alpha-2 (100 kDa coated vesicle protein C) (Adaptor protein complex AP-2 subunit alpha-2) (Alpha-adaptin C) (Clathrin assembly protein complex 2 alpha-C large chain)	0.159289	0.0602493	-0.0990401
Q9JJI8	RL38	60S ribosomal protein L38	0.158544	0.0385331	-0.121082
P51660	DHB4	Peroxisomal multifunctional enzyme type 2 (MFE-2) (17-beta-hydroxysteroid dehydrogenase 4) (17-beta-HSD 4) (D-bifunctional protein) (DBP)	0.158309	0.125424	-0.0330902
Q9Z2X1	HNRPF	Heterogeneous nuclear ribonucleoprotein F (hnRNP F) [Cleaved into: Heterogeneous nuclear ribonucleoprotein F, N-terminally processed]	0.158298	0.127179	-0.0301249
Q9Z1T1	AP3B1	AP-3 complex subunit beta-1 (Adaptor protein complex AP-3 subunit beta-1) (Clathrin assembly protein complex 3 beta-1 large chain)	0.158224	0.18824	0.0300907
Q6ZWN5	RS9	40S ribosomal protein S9	0.157682	0.077596	-0.0800862
P20152	VIME	Vimentin	0.157335	0.242728	0.0862204
Q9D0E1	HNRPM	Heterogeneous nuclear ribonucleoprotein M (hnRNP M)	0.156891	0.269153	0.113592
P06745	G6PI	Glucose-6-phosphate isomerase (GPI) (EC 5.3.1.9) (Autocrine motility factor) (AMF) (Neuroleukin) (NLK) (Phosphoglucose isomerase) (PGI) (Phosphohexose isomerase) (PHI)	0.1563	0.142032	-0.0145709
Q9JMA1	UBP14	Ubiquitin carboxyl-terminal hydrolase 14 (EC 3.4.19.12) (Deubiquitinating enzyme 14) (Ubiquitin-specific-processing protease 14)	0.156061	0.240824	0.084763
P38647	GRP75	Stress-70 protein, mitochondrial (75 kDa glucose-regulated protein) (GRP-75) (Heat shock 70 kDa protein 9) (Mortalin) (Peptide-binding protein 74)	0.155515	0.228041	0.0725657
P53810	PIPNA	Phosphatidylinositol transfer protein alpha isoform (PI-TP-alpha) (PtdIns transfer protein alpha) (PtdInsTP alpha)	0.155392	0.157563	0.00210111

P62830	RL23	60S ribosomal protein L23	0.154859	0.0937685	-0.0610584
P62320	SMD3	Small nuclear ribonucleoprotein Sm D3 (Sm-D3) (snRNP core protein D3)	0.154469	0.115645	-0.0405633
Q99PT1	GDIR1	Rho GDP-dissociation inhibitor 1 (Rho GDI 1) (GDI-1) (Rho-GDI alpha)	0.153624	0.179874	0.0196032
Q99LR1	ABD12	Monoacylglycerol lipase ABHD12 (EC 3.1.1.23) (2-arachidonoylglycerol hydrolase) (Abhydrolase domain-containing protein 12)	0.153331	0.214905	0.0615749
Q61171	PRDX2	Peroxiredoxin-2 (EC 1.11.1.15) (Thiol-specific antioxidant protein) (TSA) (Thioredoxin peroxidase 1)	0.152708	0.142069	-0.00693782
P17225	PTBP1	Polypyrimidine tract-binding protein 1 (PTB) (Heterogeneous nuclear ribonucleoprotein I) (hnRNP I)	0.151561	0.243798	0.0915915
P68254	1433T	14-3-3 protein theta (14-3-3 protein tau)	0.14863	0.267793	0.111851
Q7TMK9	HNRPQ	Heterogeneous nuclear ribonucleoprotein Q (hnRNP Q) (Glycine- and tyrosine-rich RNA-binding protein) (GRY-RBP) (NS1-associated protein 1) (Synaptotagmin-binding, cytoplasmic RNA-interacting protein) (pp68)	0.148192	0.0331038	-0.115088
Q9D6F9	TBB4A	Tubulin beta-4A chain (Tubulin beta-4 chain)	0.147708	0.106908	-0.0408007
Q8VEK3	HNRPU	Heterogeneous nuclear ribonucleoprotein U (hnRNP U) (Scaffold-attachment factor A) (SAF-A)	0.147208	0.194316	0.0471076
Q922R8	PDIA6	Protein disulfide-isomerase A6 (EC 5.3.4.1) (Thioredoxin domain-containing protein 7)	0.146887	0.115692	-0.0337717
Q9WVE8	PACN2	Protein kinase C and casein kinase substrate in neurons protein 2 (Syndapin-2) (Syndapin-II)	0.146437	0.183471	0.0370344
Q8BH43	WASF2	Wiskott-Aldrich syndrome protein family member 2 (WASP family protein member 2) (Protein WAVE-2)	0.142099	0.355706	0.215293
Q5XJY5	COPD	Coatomer subunit delta (Archain) (Delta-coat protein) (Delta-COP)	0.14204	0.250848	0.108808
P27773	PDIA3	Protein disulfide-isomerase A3 (EC 5.3.4.1) (58 kDa glucose-regulated protein) (58 kDa microsomal protein) (p58) (Disulfide isomerase ER-60) (Endoplasmic reticulum resident protein 57) (ERp57) (ERp60)	0.141363	0.153924	0.00970338
O08553	DPYL2	Dihydropyrimidinase-related protein 2 (DRP-2) (Unc-33-like phosphoprotein 2) (ULIP-2)	0.140935	0.162626	0.022201

O88569	ROA2	Heterogeneous nuclear ribonucleoproteins A2/B1 (hnRNP A2/B1)	0.140049	0.231967	0.100627
P62309	RUXG	Small nuclear ribonucleoprotein G (snRNP-G) (Sm protein G) (Sm-G)	0.139161	0.0888309	-0.0503298
Q9WU78	PDC6I	Programmed cell death 6-interacting protein (ALG-2-interacting protein 1, X) (E2F1-inducible protein) (Eig2)	0.138754	0.174781	0.0311569
Q60668	HNRPD	Heterogeneous nuclear ribonucleoprotein D0 (hnRNP D0) (AU-rich element RNA-binding protein 1)	0.135856	0.242125	0.102602
Q3TW96	UAP1L	UDP-N-acetylhexosamine pyrophosphorylase-like protein 1	0.134974	0.14583	0.0117491
Q78PY7	SND1	Staphylococcal nuclease domain-containing protein 1 (100 kDa coactivator) (p100 co-activator)	0.134877	0.133455	-0.00368401
P20029	BIP	Endoplasmic reticulum chaperone BiP (EC 3.6.4.10) (78 kDa glucose-regulated protein) (GRP-78) (Binding-immunoglobulin protein) (BiP) (HSP70 family protein 5)	0.134609	0.0941434	-0.0396962
P51410	RL9	60S ribosomal protein L9	0.134184	0.0731848	-0.0623725
Q99LX0	PARK7	Protein/nucleic acid deglycase DJ-1 (EC 3.1.2.-) (Parkinson disease protein 7 homolog) (Parkinsonism-associated deglycase) (Protein DJ-1)	0.133982	0.213278	0.0796062
Q9Z2U0	PSA7	Proteasome subunit alpha type-7 (EC 3.4.25.1) (Proteasome subunit RC6-1)	0.133349	0.44928	0.31593
P97372	PSME2	Proteasome activator complex subunit 2 (11S regulator complex subunit beta) (REG-beta) (Proteasome activator 28 subunit beta) (PA28b)	0.133052	0.166843	0.036145
P24452	CAPG	Macrophage-capping protein (Actin regulatory protein CAP-G) (Actin-capping protein GCAP39) (Myc basic motif homolog 1)	0.132841	0.183731	0.0534846
Q61599	GDIR2	Rho GDP-dissociation inhibitor 2 (Rho GDI 2) (D4) (Rho-GDI beta)	0.129472	0.267363	0.134799
P62717	RL18A	60S ribosomal protein L18a	0.12884	0.131114	0.00227402
O70435	PSA3	Proteasome subunit alpha type-3 (EC 3.4.25.1) (Proteasome component C8) (Proteasome subunit K)	0.125399	0.236334	0.110826
Q9D1Q6	ERP44	Endoplasmic reticulum resident protein 44 (ER protein 44) (ERp44) (Thioredoxin domain-containing protein 4)	0.124941	0.320041	0.1951

P34884	MIF	Macrophage migration inhibitory factor (MIF) (EC 5.3.2.1) (Delayed early response protein 6) (DER6) (Glycosylation-inhibiting factor) (GIF)	0.124717	0.156745	0.0353996
Q99020	ROAA	Heterogeneous nuclear ribonucleoprotein A/B (hnRNP A/B) (CArG-binding factor-A) (CBF-A)	0.124115	0.0175127	-0.133366
Q11136	PEPD	Xaa-Pro dipeptidase (X-Pro dipeptidase) (EC 3.4.13.9) (Imidodipeptidase) (Peptidase 4) (Peptidase D) (Proline dipeptidase)	0.123664	0.0744235	-0.0484021
P26041	MOES	Moesin (Membrane-organizing extension spike protein)	0.123456	0.173837	0.0488237
Q9DBJ1	PGAM1	Phosphoglycerate mutase 1 (EC 5.4.2.11) (EC 5.4.2.4) (BPG-dependent PGAM 1) (Phosphoglycerate mutase isozyme B) (PGAM-B)	0.122915	0.256389	0.127495
P07356	ANXA2	Annexin A2 (Annexin II) (Annexin-2) (Calpactin I heavy chain) (Calpactin-1 heavy chain) (Chromobindin-8) (Lipocortin II) (PAP-IV) (Protein I) (p36)	0.122392	0.266206	0.144492
Q01853	TERA	Transitional endoplasmic reticulum ATPase (TER ATPase) (EC 3.6.4.6) (15S Mg (2+)-ATPase p97 subunit) (Valosin-containing protein) (VCP)	0.122185	0.169218	0.0459627
Q9JII6	AK1A1	Alcohol dehydrogenase [NADP (+)] (EC 1.1.1.2) (Aldehyde reductase) (Aldo-keto reductase family 1 member A1)	0.121045	0.203285	0.0742131
Q8CAQ8	MIC60	MICOS complex subunit Mic60 (Mitochondrial inner membrane protein)	0.120905	0.43829	0.302571
Q9WUA3	PFKAP	ATP-dependent 6-phosphofructokinase, platelet type (ATP-PFK) (EC 2.7.1.11) (6-phosphofructokinase type C) (Phosphohexokinase)	0.120901	0.211992	0.0910908
Q9CY58	PAIRB	Plasminogen activator inhibitor 1 RNA-binding protein (PAI1 RNA-binding protein 1) (PAI-RBP1) (SERPINE1 mRNA-binding protein 1)	0.119525	-0.0672535	-0.186779
P62858	RS28	40S ribosomal protein S28	0.117415	0.336145	0.213021
Q9Z0P5	TWF2	Twinfilin-2 (A6-related protein) (mA6RP) (Twinfilin-1-like protein)	0.115731	0.173555	0.0578235
Q9DCN2	NB5R3	NADH-cytochrome b5 reductase 3 (B5R) (Cytochrome b5 reductase) (EC 1.6.2.2) (Diaphorase-1)	0.11532	0.0825645	-0.0370285
O89086	RBM3	RNA-binding protein 3 (RNA-binding motif protein 3)	0.114658	0.0442011	-0.0835664

Q9CQM5	TXD17	Thioredoxin domain-containing protein 17 (14 kDa thioredoxin-related protein) (TRP14) (Protein 42-9-9) (Thioredoxin-like protein 5)	0.114445	0.250851	0.136406
Q921F2	TADBP	TAR DNA-binding protein 43 (TDP-43)	0.113164	0.109261	-0.00390279
P10639	THIO	Thioredoxin (Trx) (ATL-derived factor) (ADF)	0.110516	0.261092	0.151427
Q9JMH6	TRXR1	Thioredoxin reductase 1, cytoplasmic (TR) (EC 1.8.1.9) (Thioredoxin reductase TR1)	0.113085	0.0397	-0.0733854
P16125	LDHB	L-lactate dehydrogenase B chain (LDH-B) (EC 1.1.1.27) (LDH heart subunit) (LDH-H)	0.110434	0.409705	0.299271
Q8R081	HNRPL	Heterogeneous nuclear ribonucleoprotein L (hnRNP L)	0.109368	0.161397	0.0521776
P47753	CAZA1	F-actin-capping protein subunit alpha-1 (CapZ alpha-1)	0.108059	0.181642	0.0730544
O88456	CPNS1	Calpain small subunit 1 (CSS1) (Calcium-activated neutral proteinase small subunit) (Calcium-dependent protease small subunit 1)	0.106521	0.163071	0.0540525
P31996	CD68	Macrosialin (CD antigen CD68)	0.105014	0.242079	0.137065
P61804	DAD1	Dolichyl-diphosphooligosaccharide--protein glycosyltransferase subunit DAD1 (Oligosaccharyl transferase subunit DAD1) (Defender against cell death 1) (DAD-1)	0.105558	0.0311866	-0.0743717
Q93092	TALDO	Transaldolase (EC 2.2.1.2)	0.10391	0.244167	0.13672
P01887	B2MG	Beta-2-microglobulin	0.103793	0.138551	0.0347586
Q9D8U8	SNX5	Sorting nexin-5	0.100504	0.178017	0.0606048
Q09014	NCF1	Neutrophil cytosol factor 1 (NCF-1) (47 kDa neutrophil oxidase factor) (NCF-47K) (Neutrophil NADPH oxidase factor 1) (p47-phox)	0.0990692	0.0088996 4	-0.0901758
Q99P72	RTN4	Reticulon-4 (Neurite outgrowth inhibitor) (Nogo protein)	0.0985335	0.22753	0.125767
P45376	ALDR	Aldose reductase (AR) (EC 1.1.1.21) (Aldehyde reductase)	0.0982459	0.166156	0.0608485
Q9R1P0	PSA4	Proteasome subunit alpha type-4 (EC 3.4.25.1) (Macropain subunit C9) (Proteasome component C9) (Proteasome subunit L)	0.0967051	0.123713	0.0269524
O35601	FYB1	FYN-binding protein 1 (Adhesion and degranulation promoting adaptor protein) (ADAP) (FYB-120/130)	0.0959781	0.328487	0.232509

		(p120/p130) (FYN-T-binding protein) (SLAP-130) (SLP-76-associated phosphoprotein)			
P35700	PRDX1	Peroxiredoxin-1 (EC 1.11.1.15) (Macrophage 23 kDa stress protein) (Osteoblast-specific factor 3) (OSF-3) (Thioredoxin peroxidase 2)	0.0954113	0.227553	0.133915
P52480	KPYM	Pyruvate kinase PKM (EC 2.7.1.40) (Pyruvate kinase muscle isozyme)	0.0925438	0.245323	0.150411
P50543	S10AB	Protein S100-A11 (Calgizzarin) (Endothelial monocyte-activating polypeptide) (EMAP) (Protein S100-C) (S100 calcium-binding protein)	0.092276	0.0279363	-0.0665623
Q9Z2U1	PSA5	Proteasome subunit alpha type-5 (EC 3.4.25.1) (Macropain zeta chain) (Multicatalytic endopeptidase complex zeta chain)	0.0921232	0.091452	-0.00324742
P09103	PDIA1	Protein disulfide-isomerase (PDI) (EC 5.3.4.1) (Cellular thyroid hormone-binding protein) (Endoplasmic reticulum resident protein 59) (ER protein 59)	0.0914343	0.212553	0.12235
Q99L47	F10A1	Hsc70-interacting protein (Hip) (Protein FAM10A1) (Protein ST13 homolog)	0.0913133	0.20054	0.112692
Q07813	BAX	Apoptosis regulator BAX	0.090431	-0.0098804	-0.100283
Q9Z204	HNRPC	Heterogeneous nuclear ribonucleoproteins C1/C2 (hnRNP C1/C2)	0.0893422	0.100303	0.013321
P23492	PNPH	Purine nucleoside phosphorylase (PNP) (EC 2.4.2.1) (Inosine phosphorylase) (Inosine-guanosine phosphorylase)	0.0882077	0.13205	0.0432648
Q9JLJ2	AL9A1	4-trimethylaminobutyraldehyde dehydrogenase (TMABADH) (EC 1.2.1.47) (Aldehyde dehydrogenase family 9 member A1)	0.0880616	0.246708	0.158118
Q9CR57	RL14	60S ribosomal protein L14	0.0869769	-0.166207	-0.228672
Q8VCT3	AMPB	Aminopeptidase B (AP-B) (EC 3.4.11.6) (Arginine aminopeptidase) (Arginyl aminopeptidase) (Cytosol aminopeptidase IV)	0.0895526	0.226823	0.13727
Q3THE2	ML12B	Myosin regulatory light chain 12B (Myosin regulatory light chain 2-B, smooth muscle isoform) (Myosin regulatory light chain 20 kDa)	0.0871667	0.0671633	-0.0200035

P61255	RL26	60S ribosomal protein L26 (Silica-induced gene 20 protein) (SIG-20)	0.0836063	0.0691709	-0.0107033
Q80UG5	9-Sep	Septin-9 (SL3-3 integration site 1 protein)	0.0824296	0.247112	0.164683
P62911	RL32	60S ribosomal protein L32	0.0816107	0.0560533	-0.0136549
Q9QUI0	RHOA	Transforming protein RhoA	0.0815879	0.120164	0.0377873
P62259	1433E	14-3-3 protein epsilon (14-3-3E)	0.0814298	0.298588	0.186521
Q9D1G1	RAB1B	Ras-related protein Rab-1B	0.0792534	-0.0694147	-0.148668
Q99KC8	VMA5A	von Willebrand factor A domain-containing protein 5A (Loss of heterozygosity 11 chromosomal region 2 gene A protein homolog)	0.0781739	0.200521	0.12188
Q9DCD0	6PGD	6-phosphogluconate dehydrogenase, decarboxylating (EC 1.1.1.44)	0.0762118	0.25978	0.180208
Q9R1P3	PSB2	Proteasome subunit beta type-2 (EC 3.4.25.1) (Macropain subunit C7-I) (Proteasome component C7-I)	0.0738221	0.168058	0.094236
Q8QZT1	THIL	Acetyl-CoA acetyltransferase, mitochondrial (EC 2.3.1.9) (Acetoacetyl-CoA thiolase)	0.0735143	0.180526	0.107012
P05555	ITAM	Integrin alpha-M (CD11 antigen-like family member B) (CR-3 alpha chain) (Cell surface glycoprotein MAC-1 subunit alpha) (Leukocyte adhesion receptor MO1) (CD antigen CD11b)	0.0727001	0.205852	0.133501
P23116	EIF3A	Eukaryotic translation initiation factor 3 subunit A (eIF3a) (Centrosomin) (Eukaryotic translation initiation factor 3 subunit 10) (eIF-3-theta) (eIF3 p167) (eIF3 p180) (eIF3 p185) (p162)	0.0724149	0.0958395	0.0234246
P57780	ACTN4	Alpha-actinin-4 (Non-muscle alpha-actinin 4)	0.0720584	0.218176	0.135612
P97429	ANXA4	Annexin A4 (Annexin IV) (Annexin-4)	0.071741	0.207221	0.134202
P97449	AMPN	Aminopeptidase N (AP-N) (mAPN) (EC 3.4.11.2) (Alanyl aminopeptidase) (Aminopeptidase M) (AP-M) (Membrane protein p161) (Microsomal aminopeptidase) (CD antigen CD13)	0.0712987	0.170852	0.100095
O08709	PRDX6	Peroxiredoxin-6 (EC 1.11.1.15) (1-Cys peroxiredoxin) (1-Cys PRX) (Acidic calcium-independent phospholipase A2) (aiPLA2) (Antioxidant protein 2)	0.0712057	0.205	0.134233

P62806	H4	Histone H4	0.0708967	-0.0536053	-0.116702
P13020	GELS	Gelsolin (Actin-depolymerizing factor) (ADF) (Brevin)	0.0696996	0.203242	0.133061
Q60931	VDAC3	Voltage-dependent anion-selective channel protein 3 (VDAC-3) (mVDAC3) (Outer mitochondrial membrane protein porin 3)	0.0692604	0.243469	0.174209
Q99PL5	RRBP1	Ribosome-binding protein 1 (Ribosome receptor protein) (RRp) (mRRp)	0.0663269	0.0488739	-0.0163784
Q62422	OSTF1	Osteoclast-stimulating factor 1 (SH3 domain protein 3)	0.0657962	0.0708062	0.00603966
P63260	ACTG	Actin, cytoplasmic 2 (Gamma-actin) [Cleaved into: Actin, cytoplasmic 2, N-terminally processed]	0.0643597	0.15075	0.0863901
P53026	RL10A	60S ribosomal protein L10a (CSA-19) (Neural precursor cell expressed developmentally down-regulated protein 6) (NEDD-6)	0.0618514	0.0780982	0.0147209
P50518	VATE1	V-type proton ATPase subunit E 1 (V-ATPase subunit E 1) (V-ATPase 31 kDa subunit) (p31) (Vacuolar proton pump subunit E 1)	0.0605638	0.200001	0.150037
P97369	NCF4	Neutrophil cytosol factor 4 (NCF-4) (Neutrophil NADPH oxidase factor 4) (p40-phox) (p40phox)	0.0603594	0.133646	0.0732866
P08030	APT	Adenine phosphoribosyltransferase (APRT) (EC 2.4.2.7)	0.0650623	0.277355	0.212292
P10852	4F2	4F2 cell-surface antigen heavy chain (4F2hc) (Solute carrier family 3 member 2) (CD antigen CD98)	0.0620274	-0.164874	-0.231124
Q61598	GDIB	Rab GDP dissociation inhibitor beta (Rab GDI beta) (GDI-3) (Guanosine diphosphate dissociation inhibitor 2) (GDI-2)	0.0614171	0.201204	0.140002
P70296	PEBP1	Phosphatidylethanolamine-binding protein 1 (PEBP-1) (HCNPpp) [Cleaved into: Hippocampal cholinergic neurostimulating peptide]	0.0583433	0.217697	0.155842
Q8BP47	SYNC	Asparagine--tRNA ligase, cytoplasmic (EC 6.1.1.22) (Asparaginyl-tRNA synthetase) (AsnRS)	0.0576777	0.229686	0.173429
Q9DBG6	RPN2	Dolichyl-diphosphooligosaccharide--protein glycosyltransferase subunit 2 (glycosyltransferase 63 kDa subunit) (Ribophorin II)	0.0571009	0.0505783	-0.00443793
Q923D2	BLVRB	Flavin reductase (NADPH) (FR) (EC 1.5.1.30) (Biliverdin reductase B) (BVR-B) (EC 1.3.1.24)	0.0559971	0.160811	0.103277

		(Biliverdin-IX beta-reductase) (NADPH-dependent diaphorase) (NADPH-flavin reductase) (FLR)			
P21107	TPM3	Tropomyosin alpha-3 chain (Gamma-tropomyosin) (Tropomyosin-3)	0.0540047	0.238813	0.187687
P63101	1433Z	14-3-3 protein zeta/delta (Protein kinase C inhibitor protein 1) (KCIP-1) (SEZ-2)	0.052786	0.299326	0.246546
Q9Z1G3	VATC1	V-type proton ATPase subunit C 1 (V-ATPase subunit C 1) (Vacuolar proton pump subunit C 1)	0.0533165	0.102781	0.0492339
Q9JJU8	SH3L1	SH3 domain-binding glutamic acid-rich-like protein	0.0519056	0.173949	0.124808
Q9QZ88	VPS29	Vacuolar protein sorting-associated protein 29 (Vesicle protein sorting 29)	0.0517883	0.194664	0.142876
Q9WUM5	SUCA	Succinate--CoA ligase [ADP/GDP-forming] subunit alpha, mitochondrial (EC 6.2.1.4) (EC 6.2.1.5) (Succinyl-CoA synthetase subunit alpha)	0.0516102	0.0841806	0.0325704
Q6P069	SORCN	Sorcin	0.0505086	0.257595	0.207087
P63038	CH60	60 kDa heat shock protein, mitochondrial (EC 3.6.4.9) (60 kDa chaperonin) (Chaperonin 60) (CPN60) (HSP-65) (Heat shock protein 60) (HSP-60) (Hsp60) (Mitochondrial matrix protein P1)	0.049847	0.211633	0.164966
Q60605	MYL6	Myosin light polypeptide 6 (17 kDa myosin light chain) (LC17) (Myosin light chain 3) (MLC-3) (Myosin light chain alkali 3)	0.0495265	0.177531	0.121409
P29452	CASP1	Caspase-1 (CASP-1) (EC 3.4.22.36) (Interleukin-1 beta convertase) (IL-1BC) (Interleukin-1 beta-converting enzyme) (ICE) (IL-1 beta-converting enzyme) (p45)	0.049394	-0.22611	-0.275504
P51569	AGAL	Alpha-galactosidase A (EC 3.2.1.22) (Alpha-D-galactosidase A) (Alpha-D-galactoside galactohydrolase) (Melibiase)	0.0484236	0.0903906	0.0419669
P35564	CALX	Calnexin	0.0489679	-0.0659641	-0.120411
Q9CQ22	LTOR1	Ragulator complex protein LAMTOR1 (Late endosomal/lysosomal adaptor and MAPK and MTOR activator 1) (Lipid raft adaptor protein p18)	0.0482452	0.186246	0.138
P23198	CBX3	Chromobox protein homolog 3 (Heterochromatin protein 1 homolog gamma) (HP1 gamma) (M32) (Modifier 2 protein)	0.0467572	0.14328	0.0965226

Q3U7R1	ESYT1	Extended synaptotagmin-1 (E-Syt1) (Membrane-bound C2 domain-containing protein)	0.046309	0.110078	0.0766477
Q9CPR4	RL17	60S ribosomal protein L17	0.0432682	0.044729	0.000309058
P49722	PSA2	Proteasome subunit alpha type-2 (EC 3.4.25.1) (Multicatalytic endopeptidase complex subunit C3) (Proteasome component C3)	0.0430896	-0.0661749	-0.109264
Q8K1X4	NCKPL	Nck-associated protein 1-like (Hematopoietic protein 1)	0.0384984	0.236622	0.196698
P14152	MDHC	Malate dehydrogenase, cytoplasmic (EC 1.1.1.37) (Cytosolic malate dehydrogenase)	0.0378427	0.183291	0.137749
P99029	PRDX5	Peroxiredoxin-5, mitochondrial (EC 1.11.1.15) (Antioxidant enzyme B166) (AOEB166) (Liver tissue 2D-page spot 2D-0014IV) (PLP) (Peroxiredoxin V)	0.0378868	0.194749	0.156446
P20491	FCERG	High affinity immunoglobulin epsilon receptor subunit gamma (Fc receptor gamma-chain) (IgE Fc receptor subunit gamma) (FceRI gamma)	0.0367334	0.0524037	0.0156703
Q9CWK8	SNX2	Sorting nexin-2	0.0357579	0.165495	0.129679
P29351	PTN6	Tyrosine-protein phosphatase non-receptor type 6 (EC 3.1.3.48) (70Z-SHP) (Hematopoietic cell protein-tyrosine phosphatase) (PTPTY-42) (Protein-tyrosine phosphatase 1C) (PTP-1C) (SH-PTP1) (SHP-1)	0.0339236	0.156253	0.12233
Q8R1F1	NIBL1	Niban-like protein 1 (Protein FAM129B)	0.0338941	0.234289	0.200395
P62874	GBB1	Guanine nucleotide-binding protein G(I)/G(S)/G(T) subunit beta-1 (Transducin beta chain 1)	0.0324136	0.254163	0.225669
Q9D0J8	PTMS	Parathymosin	0.0309817	0.192448	0.16393
Q04447	KCRB	Creatine kinase B-type (EC 2.7.3.2) (B-CK) (Creatine kinase B chain) (Creatine phosphokinase B-type) (CPK-B)	0.0296059	0.225282	0.198388
P40336	VP26A	Vacuolar protein sorting-associated protein 26A (H<beta>58 protein) (H beta 58) (Vesicle protein sorting 26A) (mVPS26)	0.029283	0.25213	0.222847
P06797	CATL1	Cathepsin L1 (EC 3.4.22.15) (Cathepsin L) (Major excreted protein) (MEP) (p39 cysteine proteinase)	0.029281	0.217746	0.188465

P62814	VATB2	V-type proton ATPase subunit B, brain isoform (V-ATPase subunit B 2) (Endomembrane proton pump 58 kDa subunit)	0.0288344	0.155498	0.127502
P97352	S10AD	Protein S100-A13 (S100 calcium-binding protein A13)	0.0286218	0.223658	0.195036
P05202	AATM	Aspartate aminotransferase, mitochondrial (mAspAT) (EC 2.6.1.1) (EC 2.6.1.7) (Fatty acid-binding protein) (FABP-1) (Glutamate oxaloacetate transaminase 2) (Kynurenine aminotransferase 4)	0.028306	0.22099	0.192298
Q3UIA2	RHG17	Rho GTPase-activating protein 17 (Neuron-associated developmentally-regulated protein) (Nadrin) (Rho-type GTPase-activating protein 17)	0.0281931	0.179787	0.151593
Q8BK64	AHSA1	Activator of 90 kDa heat shock protein ATPase homolog 1 (AHA1)	0.0264092	0.124061	0.0994069
Q4KML4	ABRAL	Costars family protein ABRACL (ABRA C-terminal-like protein)	0.026334	0.0533511	0.0304189
P40124	CAP1	Adenylyl cyclase-associated protein 1 (CAP 1)	0.0260383	0.268571	0.243688
Q60597	ODO1	2-oxoglutarate dehydrogenase, mitochondrial (EC 1.2.4.2) (2-oxoglutarate dehydrogenase complex component E1) (OGDC-E1) (Alpha-ketoglutarate dehydrogenase)	0.0258826	0.179463	0.153581
Q9WU81	G6PT3	Glucose-6-phosphate exchanger SLC37A2 (Solute carrier family 37 member 2) (cAMP-inducible protein 2)	0.0256075	0.252961	0.225971
P47754	CAZA2	F-actin-capping protein subunit alpha-2 (CapZ alpha-2)	0.0247055	0.212623	0.204618
Q08857	CD36	Platelet glycoprotein 4 (Glycoprotein IIIb) (GPIIIB) (PAS IV) (PAS-4) (Platelet glycoprotein IV) (GPIV) (CD antigen CD36)	0.0237933	0.281404	0.257611
O54734	OST48	Dolichyl-diphosphooligosaccharide--protein glycosyltransferase 48 kDa subunit (DDOST 48 kDa subunit) (Oligosaccharyl transferase)	0.0229596	0.0061066 5	-0.0144212
P24369	PPIB	Peptidyl-prolyl cis-trans isomerase B (PPIase B) (EC 5.2.1.8) (CYP-S1) (Cyclophilin B) (Rotamase B) (S-cyclophilin) (SCYLP)	0.0221052	0.124973	0.103682
P63001	RAC1	Ras-related C3 botulinum toxin substrate 1 (p21-Rac1)	0.0210038	0.140094	0.11889
P51863	VA0D1	V-type proton ATPase subunit d 1 (V-ATPase subunit d 1) (P39) (Physophilin) (V-ATPase 40 kDa accessory	0.0199438	0.0537059	0.0683284

		protein) (V-ATPase AC39 subunit) (Vacuolar proton pump subunit d 1)			
Q8BHN3	GANAB	Neutral alpha-glucosidase AB (EC 3.2.1.84) (Alpha-glucosidase 2) (Glucosidase II subunit alpha)	0.0184569	-0.0431266	-0.0615835
P54227	STMN1	Stathmin (Leukemia-associated gene protein) (Leukemia-associated phosphoprotein p18) (Metablastin) (Oncoprotein 18) (Op18) (Phosphoprotein p19) (pp19) (Prosolin) (Protein Pr22) (pp17)	0.0180933	0.0106668	-0.00781598
P62897	CYC	Cytochrome c, somatic	0.0165961	0.211755	0.195159
Q07076	ANXA7	Annexin A7 (Annexin VII) (Annexin-7) (Synexin)	0.0158392	0.172555	0.156801
Q60710	SAMH1	Deoxynucleoside triphosphate triphosphohydrolase SAMHD1 (dNTPase) (EC 3.1.5.-) (Interferon-gamma-inducible protein Mg11) (SAM domain and HD domain-containing protein 1) (mSAMHD1)	0.0115837	0.12792	0.115728
O88342	WDR1	WD repeat-containing protein 1 (Actin-interacting protein 1) (AIP1)	0.0103402	0.216694	0.205321
O08997	ATOX1	Copper transport protein ATOX1 (Metal transport protein ATX1)	0.00963124	0.262946	0.24642
Q9CZ13	QCR1	Cytochrome b-c1 complex subunit 1, mitochondrial (Complex III subunit 1) (Core protein I) (Ubiquinol-cytochrome-c reductase complex core protein 1)	0.00949902	0.107124	0.0976247
P99028	QCR6	Cytochrome b-c1 complex subunit 6, mitochondrial (Complex III subunit VIII) (Cytochrome c1 non-heme 11 kDa protein) (Mitochondrial hinge protein) (Ubiquinol-cytochrome c reductase complex 11 kDa protein)	0.00805709	0.128121	0.120064
P40240	CD9	CD9 antigen (CD antigen CD9)	0.00790572	0.0658323	0.0579266
Q9WV32	ARC1B	Actin-related protein 2/3 complex subunit 1B (Arp2/3 complex 41 kDa subunit) (p41-ARC)	0.00710387	0.216959	0.209265
P43274	H14	Histone H1.4 (H1 VAR.2) (H1e)	0.00774602	0.0767774	0.0690891
P84096	RHOG	Rho-related GTP-binding protein RhoG (Sid 10750)	0.00786866	0.324449	0.316581
Q99JI6	RAP1B	Ras-related protein Rap-1b (GTP-binding protein smg p21B)	0.00668076	0.0189608	0.0127362
Q8K124	PKHO2	Pleckstrin homology domain-containing family O member 2 (PH domain-containing family O member 2)	0.00313882	0.30498	0.305725

Q64433	CH10	10 kDa heat shock protein, mitochondrial (Hsp10) (10 kDa chaperonin) (Chaperonin 10) (CPN10)	0.00270922	0.190419	0.18771
P19783	COX41	Cytochrome c oxidase subunit 4 isoform 1, mitochondrial (Cytochrome c oxidase polypeptide IV) (COX IV-1)	0.00295165	0.146927	0.145541
P24270	CATA	Catalase (EC 1.11.1.6)	0.00287747	0.0149659	0.0120884
P67778	PHB	Prohibitin (B-cell receptor-associated protein 32) (BAP 32)	0.00172546	0.198774	0.195935
Q9DBG5	PLIN3	Perilipin-3 (Cargo selection protein TIP47) (Mannose-6-phosphate receptor-binding protein 1)	0.00114464	0.13024	0.129095
Q9D3D9	ATPD	ATP synthase subunit delta, mitochondrial (ATP synthase F1 subunit delta) (F-ATPase delta subunit)	0.00110378	0.38336	0.382256
Q9CY64	BIEA	Biliverdin reductase A (BVR A) (EC 1.3.1.24) (Biliverdin-IX alpha-reductase)	0.00119244	0.129658	0.129012
P62962	PROF1	Profilin-1 (Profilin I)	0.000831348	0.278876	0.277361
O89053	COR1A	Coronin-1A (Coronin-like protein A) (Clipin-A) (Coronin-like protein p57) (Tryptophan aspartate-containing coat protein) (TACO)	0.000690606	0.227185	0.224742
P27546	MAP4	Microtubule-associated protein 4 (MAP-4)	Not in GA	Not in GA	0.308308
Q9CQV8	1433B	14-3-3 protein beta/alpha (Protein kinase C inhibitor protein 1) (KCIP-1)	Not in GA	Not in GA	-0.101176
Q9JM76	ARPC3	Actin-related protein 2/3 complex subunit 3 (Arp2/3 complex 21 kDa subunit) (p21-ARC)	-0.00760276	0.247807	0.255385
P16045	LEG1	Galectin-1 (Gal-1) (14 kDa lectin) (Beta-galactoside-binding lectin L-14-I) (Galaptin) (Lactose-binding lectin 1) (Lectin galactoside-binding soluble 1) (S-Lac lectin 1)	-0.0260209	0.305302	0.330991
P08249	MDHM	Malate dehydrogenase, mitochondrial (EC 1.1.1.37)	-0.0308097	0.216411	0.249557
Q61233	PLSL	Plastin-2 (65 kDa macrophage protein) (L-plastin) (Lymphocyte cytosolic protein 1) (LCP-1) (pp65)	-0.0381093	0.210035	0.24787
P48036	ANXA5	Annexin A5 (Anchorin CII) (Annexin V) (Annexin-5) (Calphobindin I) (CBP-I) (Endonexin II) (Lipocortin V) (Placental anticoagulant protein 4) (PP4)	-0.00447814	0.225801	0.226325
P26443	DHE3	Glutamate dehydrogenase 1, mitochondrial (GDH 1) (EC 1.4.1.3)	-0.0108467	0.211602	0.225179

Q68FD5	CLH1	Clathrin heavy chain 1	-0.0181712	0.222248	0.241465
P61161	ARP2	Actin-related protein 2 (Actin-like protein 2)	-0.0371429	0.252718	0.291844
Q99JY9	ARP3	Actin-related protein 3 (Actin-like protein 3)	-0.0126309	0.186841	0.19961
Q8BFR5	EFTU	Elongation factor Tu, mitochondrial	-0.0490606	0.255545	0.304171
P50516	VATA	V-type proton ATPase catalytic subunit A (V-ATPase subunit A) (EC 3.6.3.14) (V-ATPase 69 kDa subunit) (Vacuolar proton pump subunit alpha)	-0.0280875	0.158365	0.184593
P56480	ATPB	ATP synthase subunit beta, mitochondrial (EC 3.6.3.14) (ATP synthase F1 subunit beta)	-0.0839579	0.134661	0.217569
Q9DCW4	ETFB	Electron transfer flavoprotein subunit beta (Beta-ETF)	-0.0614758	0.213166	0.274355
Q9DB20	ATPO	ATP synthase subunit O, mitochondrial (Oligomycin sensitivity conferral protein) (OSCP)	-0.05113	0.200865	0.240304
Q62465	VAT1	Synaptic vesicle membrane protein VAT-1 homolog (EC 1.-.-.-)	-0.0392401	0.170106	0.205155
P54071	IDHP	Isocitrate dehydrogenase [NADP], mitochondrial (IDH) (EC 1.1.1.42) (ICD-M) (IDP) (NADP(+)-specific ICDH) (Oxalosuccinate decarboxylase)	-0.0951424	0.275896	0.369896
P00493	HPRT	Hypoxanthine-guanine phosphoribosyltransferase (HGPRT) (HGPRTase) (EC 2.4.2.8) (HPRT B)	-0.0673715	0.226735	0.288216
P47757	CAPZB	F-actin-capping protein subunit beta (CapZ beta)	-0.0212923	0.268008	0.295916
Q9CPW4	ARPC5	Actin-related protein 2/3 complex subunit 5 (Arp2/3 complex 16 kDa subunit) (p16-ARC)	-0.042695	0.248302	0.26029
P59999	ARPC4	Actin-related protein 2/3 complex subunit 4 (Arp2/3 complex 20 kDa subunit) (p20-ARC)	-0.0378844	0.198655	0.237854
Q00612	G6PD1	Glucose-6-phosphate 1-dehydrogenase X (G6PD) (EC 1.1.1.49)	-0.0282586	0.254274	0.285463
Q63844	MK03	Mitogen-activated protein kinase 3 (MAP kinase 3) (MAPK 3) (EC 2.7.11.24) (ERT2) (Extracellular signal-regulated kinase 1) (ERK-1) (Insulin-stimulated MAP2 kinase) (p44-MAPK) (MNK1) (Microtubule-associated protein 2 kinase) (p44-ERK1)	-0.086459	0.263567	0.350026
Q9CZU6	CISY	Citrate synthase, mitochondrial (EC 2.3.3.1) (Citrate (Si)-synthase)	-0.0796658	0.120336	0.201803
P62331	ARF6	ADP-ribosylation factor 6	-0.0930375	0.293427	0.386464

Q9D1A2	CNDP2	Cytosolic non-specific dipeptidase (EC 3.4.13.18) (CNDP dipeptidase 2) (Glutamate carboxypeptidase-like protein 1)	-0.0132527	0.171576	0.186728
Q9CVB6	ARPC2	Actin-related protein 2/3 complex subunit 2 (Arp2/3 complex 34 kDa subunit) (p34-ARC)	-0.0432953	0.199402	0.240714
P97807	FUMH	Fumarate hydratase, mitochondrial (Fumarase) (EC 4.2.1.2) (EF-3)	-0.0569615	0.261032	0.316548
Q91YR9	PTGR1	Prostaglandin reductase 1 (PRG-1) (EC 1.3.1.-) (15-oxoprostaglandin 13-reductase) (EC 1.3.1.48) (NADP-dependent leukotriene B4 12-hydroxydehydrogenase) (EC 1.3.1.74)	-0.0712788	0.251623	0.32237
P08752	GNAI2	Guanine nucleotide-binding protein G(i) subunit alpha-2 (Adenylate cyclase-inhibiting G alpha protein)	-0.0935772	0.130592	0.220402
Q8VEM8	MPCP	Phosphate carrier protein, mitochondrial (Phosphate transport protein) (PTP) (Solute carrier family 25 member 3)	-0.0194466	0.131246	0.155672
Q9Z1G4	VPP1	V-type proton ATPase 116 kDa subunit a isoform 1 (V-ATPase 116 kDa isoform a1) (Clathrin-coated vesicle/synaptic vesicle proton pump 116 kDa subunit) (Vacuolar proton pump subunit 1)	-0.0561002	0.211588	0.267022
Q8BG05	ROA3	Heterogeneous nuclear ribonucleoprotein A3 (hnRNP A3)	-0.0596435	0.25485	0.312662
Q9WUM3	COR1B	Coronin-1B (Coronin-2)	-0.0273362	0.181984	0.20932
Q91XV3	BASP1	Brain acid soluble protein 1 (22 kDa neuronal tissue-enriched acidic protein) (Neuronal axonal membrane protein NAP-22)	-0.0870958	0.216259	0.302233
Q8K1B8	URP2	Fermitin family homolog 3 (Kindlin-3) (Unc-112-related protein 2)	-0.00128139	0.206218	0.209018
P97821	CATC	Dipeptidyl peptidase 1 (EC 3.4.14.1) (Cathepsin C) (Cathepsin J) (Dipeptidyl peptidase I) (DPP-I)	-0.0106422	0.180373	0.191015
O08756	HCD2	3-hydroxyacyl-CoA dehydrogenase type-2 (EC 1.1.1.35) (17-beta-HSD 10) (Endoplasmic reticulum-associated amyloid beta-peptide-binding protein) (Mitochondrial ribonuclease P protein 2)	-0.00171827	0.20295	0.204668

Q62393	TPD52	Tumor protein D52 (mD52)	-0.0498516	0.170845	0.219927
Q9QZQ8	H2AY	Core histone macro-H2A.1 (Histone macroH2A1) (mH2A1) (H2A.y) (H2A/y)	-0.0482354	0.198462	0.246697
Q9CPQ8	ATP5L	ATP synthase subunit g, mitochondrial (ATPase subunit g)	-0.0207829	0.175187	0.195969
O35129	PHB2	Prohibitin-2 (B-cell receptor-associated protein BAP37) (Repressor of estrogen receptor activity)	-0.0747659	0.107253	0.181662
Q99J77	SIAS	Sialic acid synthase (N-acetylneuraminate-9-phosphate synthase) (EC 2.5.1.57) (N-acetylneuraminic acid phosphate synthase)	0.0497691	0.247016	0.197247
Q07417	ACADS	Short-chain specific acyl-CoA dehydrogenase, mitochondrial (SCAD) (EC 1.3.8.1) (Butyryl-CoA dehydrogenase)	-0.0370255	0.183036	0.220062
O35405	PLD3	Phospholipase D3 (PLD 3) (EC 3.1.4.4) (Choline phosphatase 3) (Phosphatidylcholine-hydrolyzing phospholipase D3) (Schwannoma-associated protein 9) (SAM-9)	-0.0597099	0.115451	0.17516
O70492	SNX3	Sorting nexin-3 (SDP3 protein)	-0.0209234	0.117392	0.138316
Q62425	NDUA4	Cytochrome c oxidase subunit NDUFA4	-0.0836632	0.171597	0.25526
P28650	PURA1	Adenylosuccinate synthetase isozyme 1 (AMPSase 1) (AdSS 1) (EC 6.3.4.4) (Adenylosuccinate synthetase, basic isozyme)	-0.0432272	0.115936	0.15927
P45952	ACADM	Medium-chain specific acyl-CoA dehydrogenase, mitochondrial (MCAD) (EC 1.3.8.7)	-0.00357593	0.212109	0.217693
Q7TMM9	TBB2A	Tubulin beta-2A chain	-0.00607515	-0.265898	-0.259823
Q3TRM8	HXK3	Hexokinase-3 (EC 2.7.1.1) (Hexokinase type III) (HK III)	-0.0689827	0.197272	0.26551
P20108	PRDX3	Thioredoxin-dependent peroxide reductase, mitochondrial (EC 1.11.1.15) (Antioxidant protein 1) (AOP-1) (PRX III) (Perioredoxin-3) (Protein MER5)	-0.0578994	0.0997267	0.157682
Q91V61	SFXN3	Sideroflexin-3	-0.0858821	0.132869	0.218751
Q921T2	TOIP1	Torsin-1A-interacting protein 1 (Lamina-associated polypeptide 1B) (LAP1B)	-0.0658782	-0.198752	-0.132874
O08749	DLDH	Dihydrolipoyl dehydrogenase, mitochondrial (EC 1.8.1.4) (Dihydrolipoamide dehydrogenase)	-0.0784357	0.147889	0.226076

Q9CR51	VATG1	V-type proton ATPase subunit G 1 (V-ATPase subunit G 1) (V-ATPase 13 kDa subunit 1) (Vacuolar proton pump subunit G 1)	-0.0335464	0.130256	0.163803
O09159	MA2B1	Lysosomal alpha-mannosidase (Laman) (EC 3.2.1.24) (Lysosomal acid alpha-mannosidase) (Mannosidase alpha class 2B member 1)	-0.0811354	0.122306	0.19276
Q62192	CD180	CD180 antigen (Lymphocyte antigen 78) (Ly-78) (Radioprotective 105 kDa protein) (CD antigen CD180)	-0.0590868	0.159046	0.186224
P97371	PSME1	Proteasome activator complex subunit 1 (11S regulator complex subunit alpha) (REG-alpha) (Proteasome activator 28 subunit alpha)	-0.0244065	0.156131	0.180845
Q8BFZ3	ACTBL	Beta-actin-like protein 2 (Kappa-actin)	-0.000695825	0.0968645	0.0975603
Q99KQ4	NAMPT	Nicotinamide phosphoribosyltransferase (NAmPRTase) (Nampt) (EC 2.4.2.12) (Pre-B-cell colony-enhancing factor 1 homolog) (PBEF) (Visfatin)	-0.0521657	0.112394	0.16456
P14069	S10A6	Protein S100-A6 (5B10) (Calcyclin) (Prolactin receptor-associated protein) (S100 calcium-binding protein A6)	-0.0175891	0.0635069	0.0810959
Q8BG07	PLD4	Phospholipase D4 (PLD 4) (EC 3.1.4.4) (Choline phosphatase 4) (Phosphatidylcholine-hydrolyzing phospholipase D4)	-0.0728758	0.0511171	0.123993
Q5SUA5	MYO1G	Unconventional myosin-Ig	-0.00481071	0.0938804	0.0986911
P50752	TNNT2	Troponin T, cardiac muscle (TnTc) (Cardiac muscle troponin T) (cTnT)	-0.0121968	-0.370496	-0.358299
Q05144	RAC2	Ras-related C3 botulinum toxin substrate 2 (Protein EN-7) (p21-Rac2)	-0.0143211	0.0827591	0.0968894
Q8BYW1	RHG25	Rho GTPase-activating protein 25 (Rho-type GTPase-activating protein 25)	-0.00850329	0.176731	0.187298
Q60631	GRB2	Growth factor receptor-bound protein 2 (Adapter protein GRB2) (SH2/SH3 adapter GRB2)	-0.0425825	0.00922822	0.0518107
Q78XF5	OSTC	Oligosaccharyltransferase complex subunit OSTC	-0.0547099	-0.107153	-0.0524433
Q64310	SURF4	Surfeit locus protein 4	-0.0100346	-0.0514255	-0.0413909
Q9JIF7	COPB	Coatomer subunit beta (Beta-coat protein) (Beta-COP)	-0.0626974	-0.0090473	0.0536501

P51174	ACADL	Long-chain specific acyl-CoA dehydrogenase, mitochondrial (LCAD) (EC 1.3.8.8)	-0.06433	-0.0201506	0.0441794
P46460	NSF	Vesicle-fusing ATPase (EC 3.6.4.6) (N-ethylmaleimide-sensitive fusion protein) (NEM-sensitive fusion protein) (Suppressor of K(+) transport growth defect 2) (Protein SKD2) (Vesicular-fusion protein NSF)	-0.0718416	-0.0496143	0.0222273
P84091	AP2M1	AP-2 complex subunit mu (AP-2 mu chain) (Adaptor protein complex AP-2 subunit mu) (Clathrin coat-associated protein AP50) (Mu2-adaptin)	-0.00088775	-0.0219801	-0.0210924
P61028	RAB8B	Ras-related protein Rab-8B	-0.0216104	0.0127113	0.0371045
P62880	GBB2	Guanine nucleotide-binding protein G(I)/G(S)/G(T) subunit beta-2 (G protein subunit beta-2) (Transducin beta chain 2)	-0.0396587	0.0694365	0.109095
Q922Q8	LRC59	Leucine-rich repeat-containing protein 59 [Cleaved into: Leucine-rich repeat-containing protein 59, N-terminally processed]	-0.00930508	-0.0072272	0.00207783
P08207	S10AA	Protein S100-A10 (Calpactin I light chain) (Calpactin-1 light chain) (Cellular ligand of annexin II) (S100 calcium-binding protein A10) (p10 protein) (p11)	-0.0130569	0.0435486	0.0560264
P35278	RAB5C	Ras-related protein Rab-5C	-0.0667832	0.0480741	0.111115
Q9EQH3	VPS35	Vacuolar protein sorting-associated protein 35 (Maternal-embryonic 3) (Vesicle protein sorting 35)	-0.00816746	0.188117	0.194779
Q9D1D4	TMEDA	Transmembrane emp24 domain-containing protein 10 (21 kDa transmembrane-trafficking protein) (p24 family protein delta-1) (p24delta1)	-0.0353602	0.0586409	0.0937825
P97384	ANX11	Annexin A11 (Annexin XI) (Annexin-11) (Calcyclin-associated annexin 50)	-0.0596346	0.0781776	0.136464
Q9D6R2	IDH3A	Isocitrate dehydrogenase [NAD] subunit alpha, mitochondrial (EC 1.1.1.41) (Isocitric dehydrogenase subunit alpha) (NAD (+)-specific ICDH subunit alpha)	-0.0465245	0.0654432	0.111965
Q8BVE3	VATH	V-type proton ATPase subunit H (V-ATPase subunit H) (Vacuolar proton pump subunit H)	-0.00100756	0.130275	0.131382
B9EJ86	OSBL8	Oxysterol-binding protein-related protein 8 (ORP-8) (OSBP-related protein 8)	-0.00038479	0.0092604	0.0130623

Q61543	GSLG1	Golgi apparatus protein 1 (E-selectin ligand 1) (ESL-1) (Selel) (Golgi sialoglycoprotein MG-160)	-0.00612972	-0.0861204	-0.0822923
Q9DCH4	EIF3F	Eukaryotic translation initiation factor 3 subunit F (eIF3f) (Deubiquitinating enzyme eIF3f) (EC 3.4.19.12) (eIF-3-epsilon) (eIF3 p47)	-0.0302919	-0.170091	-0.13734
Q8R5A3	AB1IP	Amyloid beta A4 precursor protein-binding family B member 1-interacting protein (APBB1-interacting protein 1) (PREL-1) (Proline-rich protein 48)	-0.0129717	-0.0311458	-0.0432425
P32067	LA	Lupus La protein homolog (La autoantigen homolog) (La ribonucleoprotein)	-0.0224539	-0.065758	-0.0432442
P61027	RAB10	Ras-related protein Rab-10	-0.0503281	-0.0576519	-0.00461507
Q8BLF1	NCEH1	Neutral cholesterol ester hydrolase 1 (NCEH) (EC 3.1.1.-) (Arylacetamide deacetylase-like 1) (Chlorpyrifos oxon-binding protein) (CPO-BP)	-0.0456291	0.0545997	0.096008
P49312	ROA1	Heterogeneous nuclear ribonucleoprotein A1 (hnRNP A1) (HDP-1) (Helix-destabilizing protein) (hnRNP core protein A1)	-0.0608079	0.0482002	0.106783
P62245	RS15A	40S ribosomal protein S15a	-0.0262766	0.0241516	0.0419196
P62821	RAB1A	Ras-related protein Rab-1A (YPT1-related protein)	-0.00952174	0.128809	0.138257
Q8R0X7	SGPL1	Sphingosine-1-phosphate lyase 1 (S1PL) (SP-lyase 1) (SPL 1) (mSPL) (EC 4.1.2.27) (Sphingosine-1-phosphate aldolase)	-0.0420749	-0.0321303	0.00419521
Q9CQ60	6PGL	6-phosphogluconolactonase (6PGL) (EC 3.1.1.31)	-0.0627576	0.0107086	0.0771043
P60766	CDC42	Cell division control protein 42 homolog (G25K GTP-binding protein)	-0.015876	0.0428596	0.0654459
P43276	H15	Histone H1.5 (H1 VAR.5) (H1b)	-0.0836967	-0.032158	0.0505255
Q6IRU2	TPM4	Tropomyosin alpha-4 chain (Tropomyosin-4)	-0.0521912	-0.0386594	0.0167258
P98078	DAB2	Disabled homolog 2 (Adaptor molecule disabled-2) (Differentially expressed in ovarian carcinoma 2) (DOC-2) (Mitogen-responsive phosphoprotein)	-0.0108831	0.169434	0.180106
Q9WUM4	COR1C	Coronin-1C (Coronin-3)	-0.0154269	0.0695115	0.0844076
Q9D8Y0	EFHD2	EF-hand domain-containing protein D2 (Swiprosin-1)	0.0410008	-0.0270247	-0.0672558

O70503	DHB12	Very-long-chain 3-oxoacyl-CoA reductase (EC 1.1.1.330) (17-beta-hydroxysteroid dehydrogenase 12)	-0.0216582	-0.0304936	-0.00686541
P51150	RAB7A	Ras-related protein Rab-7a	-0.00043919	0.026499	0.0238295
P37040	NCPR	NADPH--cytochrome P450 reductase (CPR) (P450R) (EC 1.6.2.4)	-0.0191493	-0.0230485	-0.00343937
Q9JKF1	IQGA1	Ras GTPase-activating-like protein IQGAP1	-0.0270224	0.0834444	0.107297
P30681	HMGB2	High mobility group protein B2 (High mobility group protein 2) (HMG-2)	-0.0992796	-0.0792947	0.0199848
Q91YQ5	RPN1	Dolichyl-diphosphooligosaccharide--protein glycosyltransferase subunit 1 (glycosyltransferase 67 kDa subunit) (Ribophorin I) (RPN-I)	-0.0964811	-0.0377321	0.0607573
Q8K183	PDXK	Pyridoxal kinase (EC 2.7.1.35) (Pyridoxine kinase)	-0.103731	-0.0944414	0.00928998
Q62048	PEA15	Astrocytic phosphoprotein PEA-15 (15 kDa phosphoprotein enriched in astrocytes)	-0.156111	-0.17595	-0.0198388
O08585	CLCA	Clathrin light chain A (Lca)	-0.111195	0.0287431	0.145594
P30993	C5AR1	C5a anaphylatoxin chemotactic receptor 1 (C5a anaphylatoxin chemotactic receptor) (C5a-R) (C5aR) (CD antigen CD88)	-0.103305	-0.0061851	0.0971195
P56395	CYB5	Cytochrome b5	-0.106117	-0.0055841	0.100533
P42208	2-Sep	Septin-2 (Neural precursor cell expressed developmentally down-regulated protein 5) (NEDD-5)	-0.123131	-0.0457554	0.0773757
O54962	BAF	Barrier-to-autointegration factor (Breakpoint cluster region protein 1) (LAP2-binding protein 1)	-0.11411	0.0374754	0.151585
Q8R5J9	PRAF3	PRA1 family protein 3 (ADP-ribosylation factor-like protein 6-interacting protein 5) (ARL-6-interacting protein 5) (Aip-5) (Addicsin) (GTRAP3-18) (Prenylated Rab acceptor protein 2) (Protein JWa)	-0.107433	0.17401	0.281443
P03930	ATP8	ATP synthase protein 8 (A6L) (F-ATPase subunit 8)	-0.112887	0.102347	0.215234
P53994	RAB2A	Ras-related protein Rab-2A	-0.10058	0.159145	0.259724
Q9Z0M5	LICH	Lysosomal acid lipase/cholesteryl ester hydrolase (Acid cholesteryl ester hydrolase) (LAL) (EC 3.1.1.13) (Cholesteryl esterase) (Lipase A)	-0.104126	0.196387	0.300813
Q91YR1	TWF1	Twinfilin-1 (Protein A6)	-0.107713	0.320817	0.42853

Q03265	ATPA	ATP synthase subunit alpha, mitochondrial (ATP synthase F1 subunit alpha)	-0.103564	0.160858	0.264265
Q60854	SPB6	Serpin B6 (Placental thrombin inhibitor) (Proteinase inhibitor 6)	-0.100942	0.251366	0.328891
P20060	HEXB	Beta-hexosaminidase subunit beta (EC 3.2.1.52) (Beta-N-acetylhexosaminidase subunit beta) (Hexosaminidase subunit B)	-0.120915	0.175551	0.296111
Q8BH95	ECHM	Enoyl-CoA hydratase, mitochondrial (EC 4.2.1.17) (Enoyl-CoA hydratase 1) (Short-chain enoyl-CoA hydratase) (SCEH)	-0.121265	0.247349	0.368613
P12787	COX5A	Cytochrome c oxidase subunit 5A, mitochondrial (Cytochrome c oxidase polypeptide Va)	-0.112621	0.125301	0.237345
Q9DCX2	ATP5H	ATP synthase subunit d, mitochondrial (ATPase subunit d)	-0.114245	0.127525	0.238576
Q61425	HCDH	Hydroxyacyl-coenzyme A dehydrogenase, mitochondrial (HCDH) (EC 1.1.1.35) (Medium and short-chain L-3-hydroxyacyl-coenzyme A dehydrogenase) (Short-chain 3-hydroxyacyl-CoA dehydrogenase)	-0.112582	0.117272	0.229546
O70252	HMOX2	Heme oxygenase 2 (HO-2) (EC 1.14.14.18)	-0.121913	0.0761127	0.198025
P11438	LAMP1	Lysosome-associated membrane glycoprotein 1 (LAMP-1) (Lysosome-associated membrane protein 1) (120 kDa)	-0.126254	0.232383	0.353287
P56135	ATPK	ATP synthase subunit f, mitochondrial	-0.138315	0.071525	0.210802
Q8BFR4	GNS	N-acetylglucosamine-6-sulfatase (Glucosamine-6-sulfatase) (G6S)	-0.136579	0.149736	0.278015
O89023	TPP1	Tripeptidyl-peptidase 1 (TPP-1) (EC 3.4.14.9) (Lysosomal pepstatin-insensitive protease) (LPIC) (Tripeptidyl aminopeptidase) (Tripeptidyl-peptidase I) (TPP-I)	-0.136716	0.356127	0.497643
Q99JY0	ECHB	Trifunctional enzyme subunit beta, mitochondrial (TP-beta) [Includes: 3-ketoacyl-CoA thiolase (EC 2.3.1.16) (Acetyl-CoA acyltransferase) (Beta-ketothiolase)]	-0.135582	0.21306	0.352806
Q9QWR8	NAGAB	Alpha-N-acetylgalactosaminidase (EC 3.2.1.49; Alpha-galactosidase)	-0.146198	0.0961624	0.242361

P06800	PTPRC	Receptor-type tyrosine-protein phosphatase C (EC 3.1.3.48) (Leukocyte common antigen) (L-CA) (Lymphocyte antigen 5) (Ly-5) (CD antigen CD45)	-0.149285	0.0051298 3	0.159758
O35639	ANXA3	Annexin A3 (35-alpha calcimedin) (Annexin III) (Annexin-3) (Lipocortin III) (Placental anticoagulant protein III) (PAP-III)	-0.141891	0.148189	0.28962
Q99KI0	ACON	Aconitate hydratase, mitochondrial (EC 4.2.1.3) (Citrate hydro-lyase)	-0.14055	0.162807	0.316751
Q9WUU7	CATZ	Cathepsin Z (EC 3.4.18.1)	-0.148246	0.203295	0.349166
Q61335	BAP31	B-cell receptor-associated protein 31 (BCR-associated protein 31) (Bap31) (p28)	-0.152666	-0.0380024	0.114663
P70290	EM55	55 kDa erythrocyte membrane protein (p55) (Membrane protein, palmitoylated 1)	-0.168125	-0.0511253	0.121484
Q7TMB8	CYFP1	Cytoplasmic FMR1-interacting protein 1 (Specifically Rac1-associated protein 1) (Sra-1)	-0.164835	0.093373	0.258208
Q9JHS3	LTOR2	Ragulator complex protein LAMTOR2 (Endosomal adaptor protein p14) (Late endosomal/lysosomal adaptor and MAPK and MTOR activator 2)	-0.158432	0.013628	0.17206
P18760	COF1	Cofilin-1 (Cofilin, non-muscle isoform)	-0.150475	0.175361	0.3268
Q9EQP2	EHD4	EH domain-containing protein 4 (PAST homolog 2) (mPAST2)	-0.151597	0.174977	0.327107
P51881	ADT2	ADP/ATP translocase 2 (ADP, ATP carrier protein 2) (Adenine nucleotide translocator 2) (Solute carrier family 25 member 5)	-0.166393	0.127671	0.294064
Q9D051	ODPB	Pyruvate dehydrogenase E1 component subunit beta, mitochondrial (PDHE1-B) (EC 1.2.4.1)	-0.164258	0.232303	0.398239
Q99LC5	ETFA	Electron transfer flavoprotein subunit alpha, mitochondrial (Alpha-ETF)	-0.167282	0.150643	0.315734
P23780	BGAL	Beta-galactosidase (EC 3.2.1.23) (Acid beta-galactosidase) (Lactase)	-0.170664	0.134546	0.308309
O70370	CATS	Cathepsin S (EC 3.4.22.27)	-0.180126	0.112507	0.294285
P62774	MTPN	Myotrophin (Granule cell differentiation protein) (Protein V-1)	-0.174991	0.147844	0.322835
P62137	PP1A	Serine/threonine-protein phosphatase PP1-alpha catalytic subunit (PP-1A) (EC 3.1.3.16)	-0.181273	-0.0603676	0.120905

Q60932	VDAC1	Voltage-dependent anion-selective channel protein 1 (VDAC-1) (mVDAC1) (Outer mitochondrial membrane protein porin 1)	-0.173448	0.153129	0.329079
Q9D0K2	SCOT1	Succinyl-CoA:3-ketoacid coenzyme A transferase 1, mitochondrial (EC 2.8.3.5) (3-oxoacid CoA-transferase 1) (SCOT-s)	-0.177464	0.0826575	0.260121
P10649	GSTM1	Glutathione S-transferase Mu 1 (EC 2.5.1.18) (GST 1-1) (GST class-mu 1) (Glutathione S-transferase GT8.7) (pmGT10)	-0.18271	0.212381	0.394577
Q60930	VDAC2	Voltage-dependent anion-selective channel protein 2 (VDAC-2) (mVDAC2) (Outer mitochondrial membrane protein porin 2)	-0.188739	0.0876067	0.275749
Q91VR2	ATPG	ATP synthase subunit gamma, mitochondrial (ATP synthase F1 subunit gamma) (F-ATPase gamma subunit)	-0.190823	0.0866486	0.278143
Q6P5E4	UGGG1	UDP-glucose:glycoprotein glucosyltransferase 1 (UGT1) (EC 2.4.1.-) (UDP--Glc:glycoprotein glucosyltransferase)	-0.195269	0.0730804	0.26835
Q91ZX7	LRP1	Prolow-density lipoprotein receptor-related protein 1 (LRP-1) (Alpha-2-macroglobulin receptor) (A2MR) (CD antigen CD91)	-0.190152	-0.259309	-0.0835819
P17047	LAMP2	Lysosome-associated membrane glycoprotein 2 (LAMP-2) (Lysosome-associated membrane protein 2) (CD107 antigen-like family member B)	-0.197692	0.111676	0.314944
Q9ET22	DPP2	Dipeptidyl peptidase 2 (EC 3.4.14.2) (Dipeptidyl aminopeptidase II) (Dipeptidyl peptidase 7) (Quiescent cell proline dipeptidase)	-0.198085	0.188955	0.38704
P48962	ADT1	ADP/ATP translocase 1 (ADP, ATP carrier protein 1) (Adenine nucleotide translocator 1) (ANT 1) (Solute carrier family 25 member 4) (mANC1)	-0.198179	0.142368	0.345065
P47738	ALDH2	Aldehyde dehydrogenase, mitochondrial (EC 1.2.1.3) (AHD-M1) (ALDH class 2) (ALDH-E2) (ALDHI)	-0.201173	0.133392	0.330079
P20065	TYB4	Thymosin beta-4 (T beta 4) [Cleaved into: Hematopoietic system regulatory peptide (Seraspenide)]	-0.202173	0.290976	0.495344

P70248	MYO1F	Unconventional myosin-If	-0.210414	-0.0727966	0.137345
Q3TCN2	PLBL2	Putative phospholipase B-like 2 (EC 3.1.1.-) (66.3 kDa protein) (76 kDa protein) (Lamina ancestor homolog 2)	-0.213556	0.117174	0.330729
P00405	COX2	Cytochrome c oxidase subunit 2 (Cytochrome c oxidase II)	-0.216508	0.150819	0.36044
O88844	IDHC	Isocitrate dehydrogenase [NADP] cytoplasmic (IDH) (EC 1.1.1.42) (Cytosolic NADP-isocitrate dehydrogenase) (IDP) (NADP(+)-specific ICDH)	-0.222309	0.162965	0.38467
P12265	BGLR	Beta-glucuronidase (EC 3.2.1.31)	-0.23876	-0.0984417	0.138762
O88668	CREG1	Protein CREG1 (Cellular repressor of E1A-stimulated genes 1)	-0.236538	0.11247	0.348828
P29416	HEXA	Beta-hexosaminidase subunit alpha (EC 3.2.1.52) (Beta-N-acetylhexosaminidase subunit alpha) (Hexosaminidase subunit A)	-0.235797	0.0954595	0.329884
Q8BMS1	ECHA	Trifunctional enzyme subunit alpha, mitochondrial (TP-alpha) [Includes: Long-chain enoyl-CoA hydratase (EC 4.2.1.17);	-0.235081	0.0974473	0.326422
P56391	CX6B1	Cytochrome c oxidase subunit 6B1 (Cytochrome c oxidase subunit VIb isoform 1) (COX VIb-1)	-0.236948	0.14104	0.378483
P43275	H11	Histone H1.1 (H1 VAR.3) (Histone H1a) (H1a)	-0.242588	-0.164063	0.0791846
Q99L13	3HIDH	3-hydroxyisobutyrate dehydrogenase, mitochondrial (HIBADH) (EC 1.1.1.31)	-0.258916	0.0757029	0.334619
Q99L45	IF2B	Eukaryotic translation initiation factor 2 subunit 2 (Eukaryotic translation initiation factor 2 subunit beta) (eIF-2-beta)	-0.24872	-0.113576	0.0883394
Q07797	LG3BP	Galectin-3-binding protein (Cyp-C-associated protein) (CyCAP) (Lectin galactoside-binding soluble 3-binding protein) (Protein MAMA)	-0.251218	-0.069972	0.273462
P10605	CATB	Cathepsin B (EC 3.4.22.1) (Cathepsin B1)	-0.255411	0.196755	0.441995
P18242	CATD	Cathepsin D (EC 3.4.23.5)	-0.264822	0.0221245	0.285192
P16675	PPGB	Lysosomal protective protein (EC 3.4.16.5) (Carboxypeptidase C) (Carboxypeptidase L) (Cathepsin A) (Protective protein cathepsin A) (PPCA)	-0.256284	0.0729413	0.327457

P97450	ATP5J	ATP synthase-coupling factor 6, mitochondrial (ATPase subunit F6)	-0.240602	0.135098	0.375701
P14824	ANXA6	Annexin A6 (67 kDa calelectrin) (Annexin VI) (Annexin-6) (Calphobindin-II) (CPB-II) (Chromobindin-20) (Lipocortin VI) (Protein III) (p68) (p70)	-0.256353	0.0073422	0.265582
Q571E4	GALNS	N-acetylgalactosamine-6-sulfatase (EC 3.1.6.4) (Chondroitinsulfatase)	-0.272765	0.310709	0.583473
Q9EQ06	DHB11	Estradiol 17-beta-dehydrogenase 11 (EC 1.1.1.62) (17-beta-hydroxysteroid dehydrogenase 11) (17-beta-HSD 11) (17bHSD11)	-0.268652	-0.0670704	0.202426
P31786	ACBP	Acyl-CoA-binding protein (ACBP) (Diazepam-binding inhibitor) (DBI)	-0.281756	-0.277861	0.00389511
Q8VCW4	UN93B	Protein unc-93 homolog B1 (Unc-93B1)	-0.290456	-0.0306121	0.259844
Q9Z1N5	DX39B	Spliceosome RNA helicase Ddx39b (EC 3.6.4.13) (56 kDa U2AF65-associated protein) (DEAD box protein UAP56) (	-0.317103	0.0240314	0.341135
P54116	STOM	Erythrocyte band 7 integral membrane protein (Protein 7.2b) (Stomatin)	-0.321822	-0.0659425	0.25588
Q61207	SAP	Prosaposin (Sulfated glycoprotein 1) (SGP-1)	-0.323744	0.223145	0.547828
Q99P91	GPNMB	Transmembrane glycoprotein NMB (DC-HIL) (Dendritic cell-associated transmembrane protein) (Osteoactivin)	-0.337697	0.103683	0.441591
O88531	PPT1	Palmitoyl-protein thioesterase 1 (PPT-1) (EC 3.1.2.22)	-0.342176	0.305197	0.647373
Q9WV54	ASAH1	Acid ceramidase (AC) (ACDase) (Acid CDase) (EC 3.5.1.23)	-0.331208	-3.141E-06	0.338608
Q61830	MRC1	Macrophage mannose receptor 1 (MMR) (CD antigen CD206)	-0.30509	-0.0598151	0.244222
P68433	H31	Histone H3.1	-0.336502	-0.611689	-0.275187
Q9CQI6	COTL1	Coactosin-like protein	-0.345645	-0.0408737	0.287195
P28798	GRN	Granulins (PC cell-derived growth factor) (PCDGF) (Proepithelin) (PEPI) [Cleaved into: Acrogranin (Progranulin); Granulin-1;2;3;4;5;6;7;]	-0.381102	0.0690527	0.451281
Q9CQW2	ARL8B	ADP-ribosylation factor-like protein 8B (10C) (Novel small G protein indispensable for equal chromosome segregation 1)	-0.352428	-0.0944265	0.258001

Q9JHK5	PLEK	Pleckstrin	-0.311273	-0.149665	0.161607
Q9CQQ7	AT5F1	ATP synthase F (0) complex subunit B1, mitochondrial (ATP synthase subunit b) (ATPase subunit b)	-0.327713	-0.16907	0.158644
P97315	CSRP1	Cysteine and glycine-rich protein 1 (Cysteine-rich protein 1) (CRP) (CRP1)	-0.361181	-0.576121	-0.0188127
Q9CR62	M2OM	Mitochondrial 2-oxoglutarate/malate carrier protein (OGCP) (Solute carrier family 25 member 11)	-0.426687	-0.0115605	0.415127
P97797	SHPS1	Tyrosine-protein phosphatase non-receptor type substrate 1<br>(SHP substrate 1) (SHPS-1)	-0.444089	-0.106361	0.337728
Q05816	FABP5	Fatty acid-binding protein 5 (Epidermal-type fatty acid-binding protein) (E-FABP) (Keratinocyte lipid-binding protein)	-0.444619	0.0945002	0.532261
Q9ESY9	GILT	Gamma-interferon-inducible lysosomal thiol reductase (EC 1.8.) (Gamma-interferon-inducible protein IP-30) (Lysosomal thiol reductase IP30)	-0.444789	0.107366	0.552154
P17710	HXK1	Hexokinase-1 (EC 2.7.1.1) (Hexokinase type I) (HK I) (Hexokinase, tumor isozyme)	-0.487171	-0.307788	0.173029
Q9WTI7	MYO1C	Unconventional myosin-Ic (Myosin I beta) (MMI-beta) (MMIb)	-0.545102	-0.470397	0.0747043
Q8K2B3	SDHA	Succinate dehydrogenase [ubiquinone] flavoprotein subunit, mitochondrial (EC 1.3.5.1) (Flavoprotein subunit of complex II) (Fp)	-0.511204	-0.333386	0.15418
Q61878	PRG2	Bone marrow proteoglycan (BMPG) (Proteoglycan 2) [Cleaved into: Eosinophil granule major basic protein (EMBP) (MBP)]	-0.555343	0.0831049	0.635866
P08905	LYZ2	Lysozyme C-2 (EC 3.2.1.17) (1,4-beta-N-acetylmuramidase C) (Lysozyme C type M)	-0.578146	-0.117529	0.467348
Q60864	STIP1	Stress-induced-phosphoprotein 1 (STI1) (mSTI1) (Hsc70/Hsp90-organizing protein) (Hop)	-0.741401	0.281114	0.808997
P84228	H32	Histone H3.2	-0.946838	-1.12102	-0.174183

FC, Fold change
